# The landscape of *BRAF* transcript and protein variants in human cancer

**DOI:** 10.1186/s12943-017-0645-4

**Published:** 2017-04-28

**Authors:** Andrea Marranci, Zhijie Jiang, Marianna Vitiello, Elena Guzzolino, Laura Comelli, Samanta Sarti, Simone Lubrano, Cinzia Franchin, Ileabett Echevarría-Vargas, Andrea Tuccoli, Alberto Mercatanti, Monica Evangelista, Paolo Sportoletti, Giorgio Cozza, Ettore Luzi, Enrico Capobianco, Jessie Villanueva, Giorgio Arrigoni, Giovanni Signore, Silvia Rocchiccioli, Letizia Pitto, Nicholas Tsinoremas, Laura Poliseno

**Affiliations:** 1 Oncogenomics Unit, Core Research Laboratory, Istituto Toscano Tumori (ITT), AOUP, CNR-IFC, Via Moruzzi 1, 56124 Pisa, Italy; 2 University of Siena, Siena, Italy; 3 Center for Computational Science, University of Miami, Gables One Tower, Room 600 N, 1320 S. Dixie Highway, Coral Gables, FL 33146-2926 USA; 4 Scuola Superiore Sant’Anna, Pisa, Italy; 5 Institute of Clinical Physiology (IFC), CNR, Via Moruzzi 1, 56124 Pisa, Italy; 6 Department of Biomedical Sciences, University of Padova, Padova, Italy; 7 Proteomics Center, University of Padova and Azienda Ospedaliera di Padova, Padova, Italy; 8 Molecular and Cellular Oncogenesis Program & Melanoma Research Center, The Wistar Institute, Philadelphia, USA; 9 Institute of Hematology, University of Perugia, Perugia, Italy; 10 Department of Molecular Medicine, University of Padova, Padova, Italy; 11 Department of Surgery and Translational Medicine, University of Firenze, Firenze, Italy; 12 IIT@NEST, Center for Nanotechnology Innovation, Pisa, Italy

**Keywords:** *BRAF*, RNA-sequencing, Exon-spanning reads, Melanoma, Transcript variants, Protein variants

## Abstract

**Background:**

The BRAF protein kinase is widely studied as a cancer driver and therapeutic target. However, the regulation of its expression is not completely understood.

**Results:**

Taking advantage of the RNA-seq data of more than 4800 patients belonging to 9 different cancer types, we show that *BRAF* mRNA exists as a pool of 3 isoforms (reference *BRAF*, *BRAF*-X1, and *BRAF*-X2) that differ in the last part of their coding sequences, as well as in the length (*BRAF*-ref: 76 nt; *BRAF*-X1 and *BRAF*-X2: up to 7 kb) and in the sequence of their 3’UTRs. The expression levels of *BRAF*-ref and *BRAF*-X1/X2 are inversely correlated, while the most prevalent among the three isoforms varies from cancer type to cancer type. In melanoma cells, the X1 isoform is expressed at the highest level in both therapy-naïve cells and cells with acquired resistance to vemurafenib driven by *BRAF* gene amplification or expression of the Δ[3–10] splicing variant. In addition to the BRAF-ref protein, the BRAF-X1 protein (the full length as well as the Δ[3–10] variant) is also translated. The expression levels of the BRAF-ref and BRAF-X1 proteins are similar, and together they account for *BRAF* functional activities. In contrast, the endogenous BRAF-X2 protein is hard to detect because the C-terminal domain is selectively recognized by the ubiquitin-proteasome pathway and targeted for degradation.

**Conclusions:**

By shedding light on the repertoire of *BRAF* mRNA and protein variants, and on the complex regulation of their expression, our work paves the way to a deeper understanding of a crucially important player in human cancer and to a more informed development of new therapeutic strategies.

**Electronic supplementary material:**

The online version of this article (doi:10.1186/s12943-017-0645-4) contains supplementary material, which is available to authorized users.

## Background

*BRAF* is a Ser-Thr protein kinase that belongs to the highly oncogenic RAS/RAF/MEK/ERK signaling pathway. A direct effector of RAS, which induces its activation by dimerization, BRAF phosphorylates and activates MEK, which in turn phosphorylates and activates ERK [1]. Downstream of ERK, the known effectors of BRAF are mainly cytoplasmic proteins and transcription factors that promote cell survival, proliferation, and motility while inhibiting differentiation. Recently, non-coding effectors such as microRNAs and long non-coding RNAs have also been discovered [2–5].

*BRAF* plays a crucial role in human cancer. About 7% of all cancer cases carry a *BRAF* mutation, including 100% of hairy cell leukemia (HCL) cases, 50–60% of melanomas, 30–50% of papillary thyroid carcinomas, 10–20% of colorectal cancers, and 3–5% of non-small cell lung cancers. The most common mutation (accounting for up to 98% of all *BRAF* mutations) is a nucleotide substitution that transforms the Val at position 600 into Glu (V600E, 98% of cases), Lys (V600K, 5–10% of cases), or other amino acids (V600A/D/G/L/M/Q/R, up to 5% of cases). This mutation renders BRAF independent of RAS activation and constitutively active as a monomer [1, 6].

Furthermore, the causal link between mutant BRAFV600E and cancer has been shown in animal models of melanoma [7], colorectal cancer [8], lung cancer [9], and thyroid cancer [10].

Finally, thanks to the development of first- and second-generation selective inhibitors (BRAFi), mutant BRAFV600E has become a valuable therapeutic target in melanoma [11–13], and it holds promise for lung adenocarcinoma [14] and HCL [15].

The regulation of *BRAF* gene expression remains a rather unexplored field of investigation. This knowledge can contribute to a deeper understanding of the functioning and deregulation of such an important gene, in addition to more effective forms of targeted therapy.

Prompted by our recent study, in which we showed that *BRAF* mRNA exists in at least two transcript variants that differ in the very last part of their coding sequence (CDS) and in their 3’UTRs [16, 17], we undertook a comprehensive analysis of all the *BRAF* transcript variants that are expressed in 9 human cancer types. In our results, we confirm that *BRAF* mRNA is indeed a pool of 3 transcript variants, including the two on which we previously reported. We establish the existence of distinct BRAF protein variants that differ in their C-terminal domains. Finally, we provide insights into the mechanisms at the basis of the different expression levels displayed by the *BRAF* transcript and protein variants, and into their functional activities.

## Results and discussion

### Reference *BRAF* has a short 3’UTR

The sequences of human *BRAF* were retrieved from Ensembl Genome Browser (http://www.ensembl.org/index.html) and NCBI (http://www.ncbi.nlm.nih.gov/nucleotide/). As shown in Additional file 1: Figure S1, in the fall of 2015 Ensembl reported 5 *BRAF* transcript variants, the reference (*BRAF*-001) and 4 more (*BRAF*-002 to 005). Analogously, NCBI (GRCh38.p1) reported 10 *BRAF* transcript variants, the reference (NM_004333.4) and 9 more (*BRAF*-X1 to X9). *BRAF*-001 and NM_004333.4, the two reference sequences, are indistinguishable except for the length of their 18^th^ and last exon. In NM_004333.4, the 18^th^ exon (from now on E18.1) is 759 bp long. In *BRAF*-001, the 18^th^ exon (from now on E18.4) has the same starting point as E18.1, but is shorter (292 bp). This implies that NM_004333.4 and *BRAF*-001 transcripts differ in the length of their 3’UTRs (590 and 121 nt, respectively). Compared to the two reference sequences, the remaining variants display different alterations such as truncations (*BRAF*-003, 004, 005), the presence of additional exons (E19 and/or new exons 1–6 (NE1-6) in *BRAF*-002, 003, 004, 005, X1, X2, X3, X4, X5, X6, X8, X9), and the absence of reference exons (*BRAF*-X2, X5, X7, X8, X9).

**Fig. 1:**
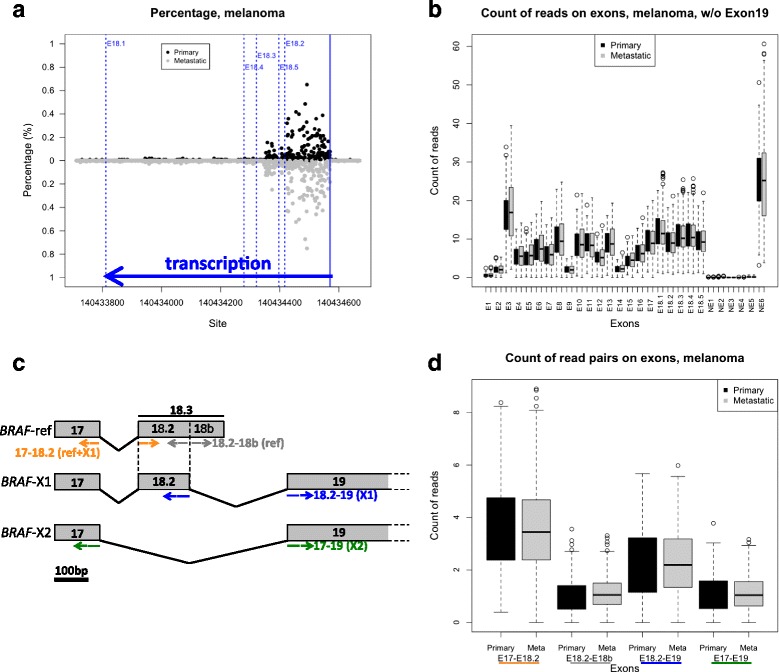
Expression of *BRAF* transcript variants in melanoma. **a** Analysis of the length of *BRAF* 3’UTR by counting the reads mapped to E18.1,2,3,4, and 5. **b** Count of the reads mapped to all *BRAF* exons, except E19. **c** Cartoon depicting the strategy used to measure the relative expression levels of *BRAF*-ref, *BRAF*-X1, and *BRAF*-X2. Paired reads spanning: exon 17 and exon 18.2 were counted as a measure of the cumulative levels of *BRAF*-ref and *BRAF*-X1 (*yellow*); exon 18.2 and exon 18b were counted as a measure of *BRAF*-ref levels (*grey*); exon 18.2 and exon 19 were counted as a measure of *BRAF*-X1 levels (*blue*); and exon 17 and exon 19 were counted as measure of *BRAF*-X2 (*green*). **d** Box plot of the reads that span E17-E18.2, E18.2-E18b, E18.2-E19, and E17-E19 in primary (*black boxes*) and metastatic (*grey boxes*) melanoma samples

To establish the actual length of the 3’UTR of reference *BRAF* (*BRAF*-ref), we considered all five variants of exon 18 retrieved from Ensembl and NCBI. We named them E18.1-5 and noticed that, though they all share the same starting point, they have different end points and thus different lengths: E18.1, reported in NM_004333.4, is 759 bp long; E18.2, reported in *BRAF*-003 and *BRAF*-X1, is 154 bp long; E18.3, reported in *BRAF*-005, *BRAF*-X3, *BRAF*-X4, *BRAF*-X6, *BRAF*-X7, *BRAF*-X8, is 250 bp long; E18.4, reported in *BRAF*-001, is 292 bp long; E18.5, reported in *BRAF*-002, is 174 bp long (Additional file 2: Table S1 for exon coordinates and length). The reads mapped to each E18 variant were counted on RNA-sequencing (RNA-seq) raw reads, which were downloaded from The Cancer Genome Atlas (TCGA). These reads belong to 4807 samples and 9 cancer types, including those in which *BRAF* mutations are frequently observed (melanoma, colon cancer, lung adenocarcinoma, and thyroid carcinoma) and others in which *BRAF* mutations are rare (breast cancer, head and neck cancer, lung squamous cell carcinoma (SCC), acute myeloid leukemia (AML), and diffuse large B-cell lymphoma (DLBCL)) (Additional file 2: Table S2). As shown in Fig. 1a for melanoma and in Additional file 1: Figure S2 for the other 8 cancer types, we observed a net drop of mapped reads at the end of E18.3. Therefore, we concluded that the last exon of *BRAF*-ref is in fact E18.3. This means that its 3’UTR is as short as 76 nt and rarely reaches the ~120 nt reported in *BRAF*-001, or the ~600 nt reported in NM_004333.4.

### The relative abundance of *BRAF*-ref, *BRAF*-X1, and *BRAF*-X2 varies from cancer type to cancer type

**Fig. 2:**
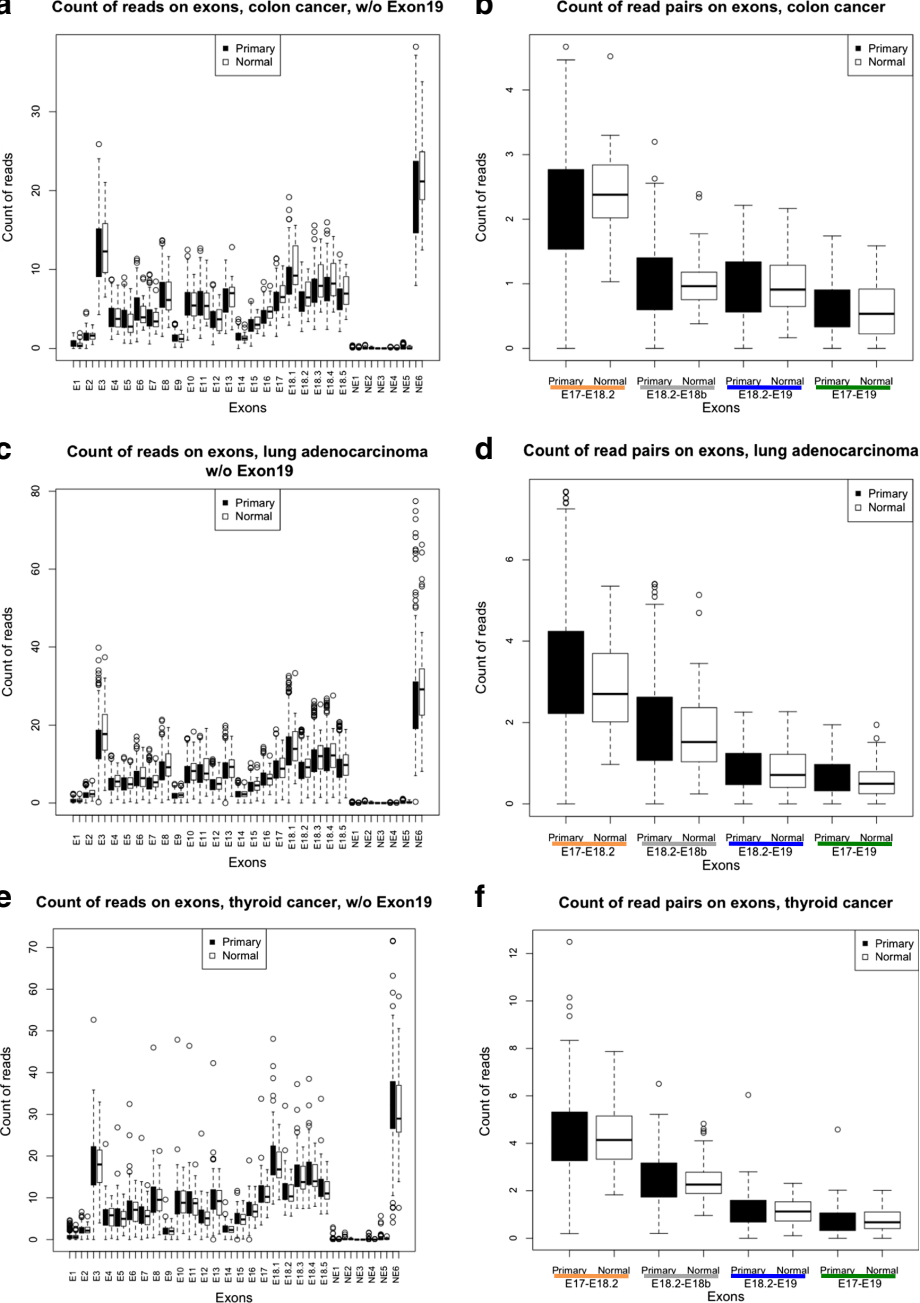
Expression of *BRAF* exons and transcript variants in colon cancer, lung adenocarcinoma and thyroid cancer. **a**, **c**, **e** Count of the reads mapped to all *BRAF* exons, except E19. (**b**, **d**, **f**) Box plot of the reads that span E17-E18.2 (*BRAF*-ref + *BRAF*-X1, *yellow*), E18.2-E18b (*BRAF*-ref, *grey*), E18.2-E19 (*BRAF*-X1, *blue*), and E17-E19 (*BRAF*-X2, *green*) in primary (*black boxes*) and normal (*white boxes*) samples

To identify transcribed variants other than *BRAF*-ref, we mapped raw reads on *BRAF* transcripts and counted mapped reads on each exon in all nine cancer types. As expected, reads that mapped to the reference exons (E1-18) were retrieved in all the cancer types we analyzed, though E1 (which contains the ATG) and E2 are mapped much less compared to the other exons (Figs. 1b and 2, left panels and Additional file 1: Figures S3 and S4). This is possibly due to a sequencing artifact. RNA-seq data obtained from polyA libraries are known to be biased against the 5’end exons [18].

For the “non-canonical” exons, we used the absence of mapped reads on NE1, NE2, NE3, and NE4 (Figs. 1b and 2, left panels and Additional file 1: Figures S3 and S4) to rule out the transcription of *BRAF*-005, and of the *BRAF*-X3, X4, X5, X6, X8, and X9 variants. Analogously, we used the absence of reads mapping to exon NE5 to rule out the transcription of *BRAF*-002.

To rule out the transcription of *BRAF*-X7 (which lacks exon 14 and 15), we used exon-spanning reads. In such reads, one of a read pair is mapped on an exon and the other is mapped on a different exon. Because of the nature of paired-end reads, exon-spanning reads can be used to assess whether two exons are expressed together. The absence of reads that span exon 13 and exon 16 (Additional file 1: Figure S5) indicates that *BRAF*-X7 is not transcribed and confirms the absence of *BRAF*-X8 and *BRAF*-X9 transcription.

For *BRAF*-003, the similar expression levels between exons 1–9 and exons 10–19 suggests it is transcribed at negligible levels.

*BRAF*-004 is reported as a truncated transcript variant in which the last exon is NE6, a 548 bp longer version of E3 (Additional file 1: Figure S6a, the NE6-specific region is named NE6p).

Since E3 and NE6 exons collect reads that belong to both full length transcripts and the *BRAF*-004 truncated transcript, we reasoned that the higher number of reads mapping to these two exons compared to the other exons (Figs. 1b and 2 left panels and Additional file 1: Figures S3 and S4) is consistent with *BRAF*-004 being transcribed. Sanger sequencing of the PCR band obtained from A375 cDNA using a forward primer located on E1/2 and a reverse primer located on NE6p confirms the transcription of NE6 (Additional file 1: Figure S6b). We also assessed the relative contribution of the *BRAF*-004 transcript variant to the pool of *BRAF* transcripts. As reported in Additional file 1: Figure S6c, we performed real-time PCR on melanoma cell lines using primers that span: the E2-E3 junction, as measure of the cumulative expression levels of full length *BRAF* transcripts and *BRAF*-004 (grey); the E3-E4 junction, as measure of the expression levels of full length *BRAF* transcripts (red); and the E3-NE6p junction, as measure of the expression levels of *BRAF*-004 (orange). We discovered that *BRAF*-004 accounts for up to 30% of the pool of *BRAF* transcripts.

Next, we assessed whether *BRAF*-004 is indeed truncated. The observation that NE6 and E4 cannot be amplified in the same PCR band (Additional file 1: Figure S6d) indicates *BRAF*-004 as truncated. This is further supported by the analysis of exon-spanning reads: as shown in Additional file 1: Figure S6e-m, NE6p-E4 spanning reads are undetectable. Due to its truncation, and thus inability to encode for a full-length protein, the *BRAF*-004 transcript variant is not analyzed further, even if it is transcribed.

The expression of the very long exon 19 (Additional file 1: Figures S3 and S4 right panels) is consistent with the transcription of the *BRAF*-X1 (XM_005250045.1) and *BRAF*-X2 (XM_005250046.1) variants. Compared to the reference sequence, in *BRAF*-X1 the shorter exon 18 (E18.2, 154 bp) does not contain the very last part of the CDS, the STOP codon and the 3’UTR. Such features are all located in the more downstream exon 19 and are therefore different [16, 17]. Compared to *BRAF*-X1, *BRAF*-X2 lacks exon 18.2 (exon 17 is spliced directly with exon 19) (Fig. 1c).

In June 2016, a new annotation (GRCh38.p8) of *BRAF* transcript variants was published in NCBI. The new *BRAF*-X3 (XM_005250045.2) is in fact indistinguishable from the old *BRAF*-X1. The new *BRAF*-X1 (XM_017012558.1) and *BRAF*-X2 (XM_017012559.1) are 120 nt longer than the corresponding old versions, due to the presence of an additional exon located between the 9^th^ and the 10^th^ (exon coordinates: 140785808–140785689). We remapped raw reads for all nine cancer types and discovered very few mapped reads on this novel exon. Therefore, we kept the annotation we were already using (GRCh38.p1).

In conclusion, this first round of analysis allowed us to establish that, in addition to the reference, two more transcript variants of *BRAF* exist, namely *BRAF*-X1 and *BRAF*-X2. To evaluate the relative contribution of the identified *BRAF* variants to the pool of *BRAF* transcripts, we again used exon-spanning reads, according to the strategy summarized in Fig. 1c. We counted the number of paired reads spanning on: exon 17 and exon 18.2, as a measure of the expression of *BRAF*-ref and *BRAF*-X1 (yellow); exon 18.2 and the *BRAF*-ref-specific part of exon 18.3 (from now on E18b), as a measure of the expression of *BRAF*-ref (grey); exon 18.2 and exon 19, as a measure of the expression of *BRAF*-X1 (blue); and exon 17 and exon 19, as a measure of the expression of *BRAF*-X2 (green).

For primary and metastatic melanoma samples, the results of this analysis are reported in Fig. 1d. We discovered that: 1, *BRAF*-X1 is expressed at higher levels than *BRAF*-ref (blue line vs grey line) and *BRAF*-X2 (blue line vs green line); and 2, *BRAF*-ref and *BRAF*-X2 are expressed at similar levels (grey line vs green line).

The RNA-seq data presented above are consistent with those we previously reported on melanoma cell lines. By performing real-time PCR detection of the reference 3’UTR and the non-canonical 3’UTR transcribed from exon 19, we obtained the ratio between them as ~1:3 [16]. To assess the relative abundance of *BRAF*-X1 and *BRAF*-X2 in the pool of exon 19-containing transcripts, we again performed real-time PCR on some of the same cell lines previously tested. However, this time we used not only the primers that detect *BRAF*-X1 and *BRAF*-X2 combined (*BRAF*-E19-1), but also those that detect the *BRAF*-X1 and *BRAF*-X2 isoforms distinctly (Additional file 1: Figure S7). As shown in Additional file 1: Figure S8a, we confirmed that irrespective of their mutational status, all the melanoma cell lines show that the expression of the exon 19-derived 3’UTR is higher than the expression of *BRAF*-ref (grey vs black). In addition, we found that the expression of the exon 19-derived 3’UTR is mostly accounted for by *BRAF*-X1 (black vs blue), while *BRAF*-X2 levels are similar to those of *BRAF*-ref (green vs grey).

From other tumors in which *BRAF* mutations are frequently observed, we obtained different results compared to melanoma: in colon cancer, the *BRAF*-ref and *BRAF*-X1 isoforms are expressed at similar levels (Fig. 2b); while in lung adenocarcinoma (Fig. 2d) and in thyroid cancer (Fig. 2f), *BRAF*-ref is in fact expressed at higher levels compared to the *BRAF*-X1 and *BRAF*-X2 isoforms. Among the other cancer types analyzed, we found that *BRAF*-ref is the most abundant isoform in breast cancer, head and neck cancer, lung SCC, and DLBCL, while *BRAF*-X1 is the most abundant isoform in AML (Additional file 1: Figure S9). Using the real-time primer pairs described above, we measured the relative expression levels of the *BRAF*-ref, *BRAF*-X1, and *BRAF*-X2 isoforms on cell lines derived from breast, cervix, colon, lung, and prostate cancer (Additional file 1: Figure S8b), as well as on leukemia/lymphoma cell lines and patient samples (Additional file 1: Figure S8c-d). Overall, we found that *BRAF*-X1 is the most expressed isoform. However, we did find cases, such as the T47D breast cancer cells and the CaCo2 colon cancer cells, in which *BRAF*-ref prevails compared to the X1 and X2 isoforms, in agreement with the RNA-seq data.

Finally, we assessed whether the difference in expression levels between *BRAF*-ref and *BRAF*-X1 is at least in part attributable to a differential stability of their RNA molecules. By treating A375 melanoma cells with actinomycin D and measuring the decay rate of the short reference 3’UTR compared to the long E19-derived 3’UTR, we discovered that the former undergoes a faster decay compared to the latter (Additional file 1: Figure S10), a finding that is consistent with the lower *BRAF*-ref vs *BRAF*-X1 levels observed in melanoma cells.

### The expression levels of *BRAF*-X1/X2 are inversely correlated with those of *BRAF*-ref

**Fig. 3:**
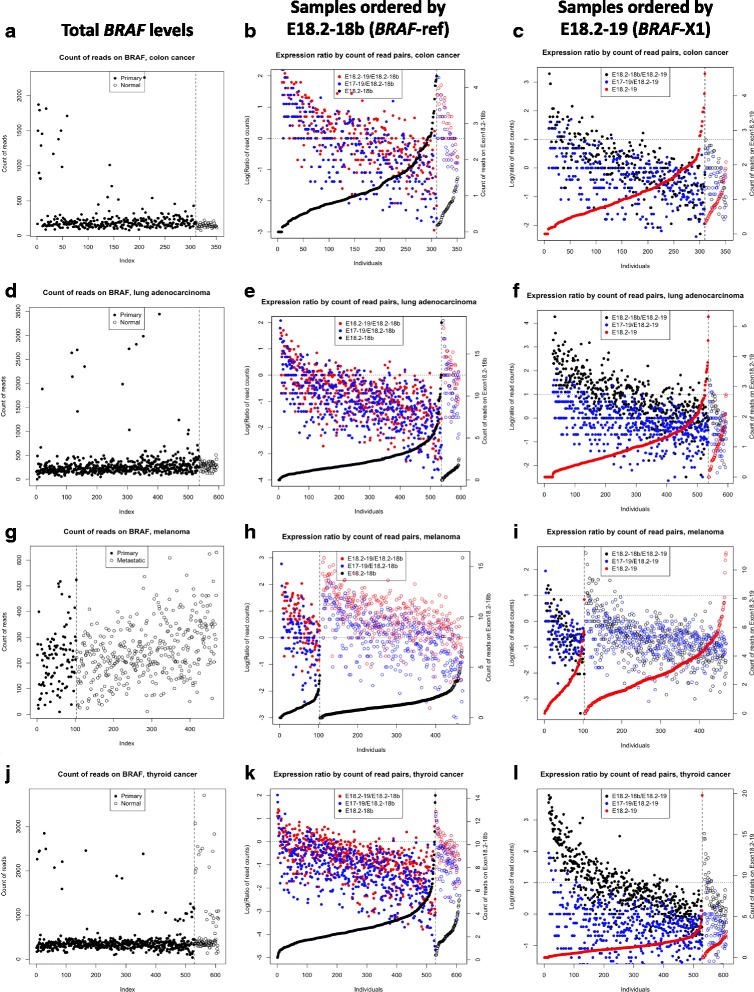
Correlation among the expression levels of the different *BRAF* isoforms in colon cancer, lung adenocarcinoma, melanoma and thyroid cancer. **a**, **d**, **g**, **j** Total number of *BRAF* reads across patients. **b**, **e**, **h**, **k** Expression ratios over the ref spanning reads. Samples were sorted by reads spanning E18.2-E18.b (*BRAF*-ref*,* in *black*). *Red dots* are E18.2-E19/E18.2-E18b ratios (which means the X1/ref ratio) and *blue dots* are E17-E19/E18.2-E18b ratios (which means the X2/ref ratio). The data points are log transformed and the *dotted line* marks the 0, which means X1/ref ratio = 1 and X2/ref ratio = 1. **c**, **f**, **i**, **l** Expression ratios over the X1 spanning reads. Samples were sorted by reads spanning E18.2-E19 (*BRAF*-X1, in *red*). *Black dots* are E18.2-E18b/E18.2-E19 ratios (which means the ref/X1 ratio) and *blue dots* are E17-E19/E18.2-E19 ratios (which means the X2/X1 ratio). The data points are log transformed and the *dotted line* marks the 0, which means ref/X1 ratio = 1 and X2/X1 ratio = 1. In the *left* and *middle* panels the samples are presented in the same order

We next assessed whether there are correlations among the expression levels of the different isoforms. First, by counting reads mapped on all *BRAF* transcripts, we found that total *BRAF* levels are similar across cancer patients belonging to the same group (Fig. 3, left panels and Additional file 1: Figure S11, left panels), though a few outliers were observed. We then sorted patients by the number of reads that span on E18.2-E18b (increasing expression level of the *BRAF*-ref variant). Finally, we calculated the E18.2-E19/E18.2-E18b ratio (the *BRAF*-X1/*BRAF*-ref ratio, in red), as well as the E17-E19/E18.2-E18b ratio (the *BRAF*-X2/*BRAF*-ref ratio, in blue) (Fig. 3, middle panels and Additional file 1: Figure S11, middle panels). The overall distribution of the data points suggest that the expression levels of *BRAF*-X1 and *BRAF*-X2 are highest where the expression level of *BRAF*-ref is lowest, and vice versa, which equals to say that the expression levels of *BRAF*-X1 and *BRAF*-X2 are inversely correlated with that of *BRAF*-ref. Furthermore, the distribution of the blue and red data points across the zero line (which in the log scale marks the *BRAF*-X1/*BRAF*-ref ratio = 1 and the *BRAF*-X2/*BRAF*-ref ratio = 1) indicates there are tumor types, such as melanoma, in which a substantial number of patients express more *BRAF*-X1 than *BRAF*-ref, and there are others, such as lung adenocarcinoma and thyroid cancer, in which a substantial number of patients express more *BRAF*-ref than the X1 isoform. These data confirm at the individual level those reported in Figs. 1d and 2b, d, f and Additional file 1: Figure S9.

We also sorted patients by the number of reads that span on E18.2-E19 (increasing expression of the *BRAF*-X1 variant, in red) and calculated the E18.2-E18b/E18.2-E19 ratio (the *BRAF*-ref/*BRAF*-X1 ratio, in black), as well as the E17-E19/E18.2-E19 ratio (the *BRAF*-X2/*BRAF*-X1 ratio, in blue) (Fig. 3, right panels and Additional file 1: Figure S11, right panels). The distribution of the black data points confirms that *BRAF*-X1 is prevalent where *BRAF*-ref is least expressed, and vice versa. Conversely, the distribution of the blue data points suggests that the expression of the X2 isoform, although always lower, follows the trend of that of the X1 isoform.

Next, we looked at melanoma samples to check whether the levels of *BRAF*-ref, *BRAF*-X1, and *BRAF*-X2 and/or their ratios are associated with clinical variables. As shown in Additional file 1: Figures S12-13, this does not seem to be the case, at least when the age, gender, and stage at diagnosis are considered.

### The 3’UTR of *BRAF*-X1 and *BRAF*-X2 is up to 7 kb long

**Fig. 4:**
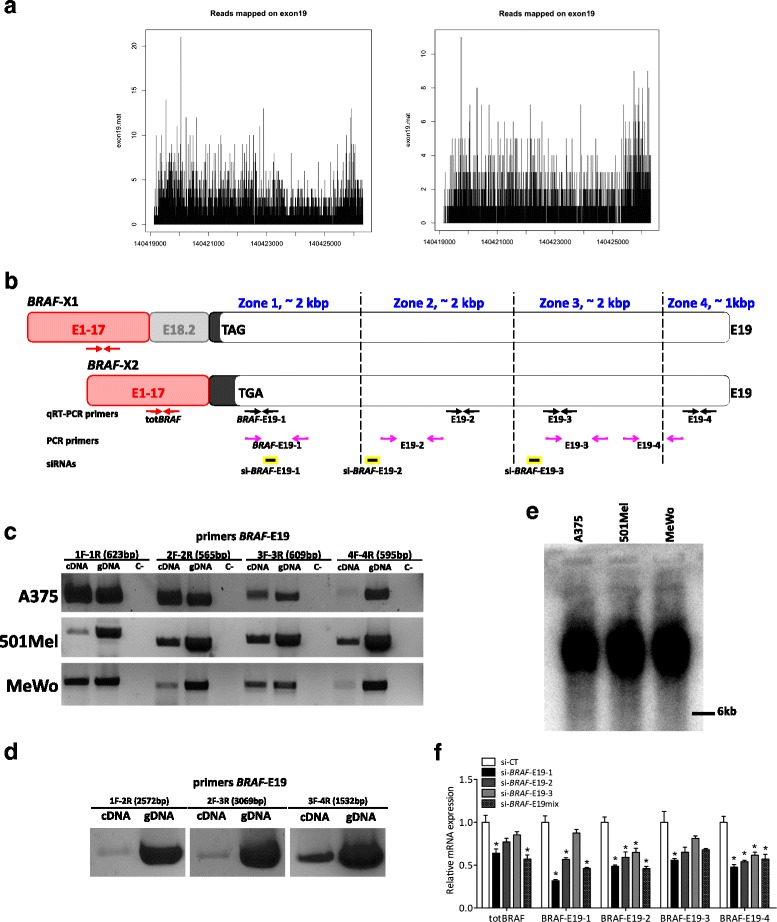
Length of *BRAF*-X1 and *BRAF*-X2 3’UTR in melanoma. **a** The analysis of the reads mapping to exon 19 across its entire length indicates that the 3’UTR of *BRAF*-X1 and *BRAF*-X2 is as long as 7 kb. A representative example of a primary (*left*) and a metastatic (*right*) melanoma case is reported. **b** Cartoon summarizing the position of the primers and the siRNAs used to determine the length of the 3’UTR of *BRAF*-X1 and *BRAF*-X2 in melanoma cell lines. The 4 primer pairs used for real-time PCR amplification of *BRAF*-X1 plus X2 (*BRAF*-E19-1/2/3/4 qRT-PCR F/R) are represented as *black arrows*. The 4 primer pairs used for PCR amplification of *BRAF*-X1 plus X2 (*BRAF*-E19-1/2/3/4 F/R) are represented as open *pink arrows. BRAF*-E19-1 qRT-PCR F and *BRAF*-E19-1 F have the same sequence. The siRNAs used to knock-down *BRAF*-X1 plus X2 (si-*BRAF*-E19-1/2/3) are represented as *yellow* and *black rectangles*. The primers used for real-time PCR amplification of all *BRAF* isoforms (tot*BRAF* qRT-PCR F/R) are represented as *red arrows*. **c** PCR performed on A375, 501Mel and MeWo melanoma cells using *BRAF*-E19-1/2/3/4 primer pairs. Genomic DNA (gDNA) is used as positive control. **d** Chained PCR performed on A375 melanoma cells using “hybrid” primer pairs: *BRAF*-E19-1 F with *BRAF*-E19-2 R, *BRAF*-E19-2 F with *BRAF*-E19-3 R, and *BRAF*-E19-3 F with *BRAF*-E19-4 R. Genomic DNA (gDNA) is used as positive control. **e** Northern blot of total RNA extracted from A375, 501Mel, and MeWo cells and hybridized with a labeled probe for *BRAF* CDS. Considering that the size of *BRAF* CDS is about 2.3 kb, the band detected in all cell lines is consistent with a 7 kb long 3’UTR. **f** Expression levels of *BRAF* CDS (detected using the tot*BRAF* qRT-PCR primers) and of different regions of the 3’UTR transcribed from E19 (detected using the *BRAF*-E19-1/2/3/4 qRT-PCR primer pairs) after the transfection of the indicated siRNAs. The pictures are taken from 1 out of 3 independent experiments performed, all with comparable outcome. The *graphs* represent the mean ± SEM of 3 independent experiments. *p < 0.05

We then investigated whether in melanoma cells the 3’UTR of *BRAF*-X1 and *BRAF*-X2, which is transcribed from exon 19, is indeed as long as 7 kb (Additional file 1: Figure S1). RNA-seq data suggested us that this is the case, since reads distribution is homogeneous across the entire length of E19 (Fig. 4a). Nevertheless, we verified this piece of evidence by performing PCR on A375 (homozygous for BRAFV600E), 501Mel (heterozygous for BRAFV600E), and MeWo (wtBRAF) melanoma cell lines, using multiple primer pairs that are located across the entire 3’UTR length (Fig. 4b). As shown in Fig. 4c, we detected a PCR product with each of the four primer pairs used. Furthermore, using the chained PCR approach reported in Fig. 4d we established that the amplified fragments are linked together, hence they belong to the same RNA molecule.

To prove that complete *BRAF* transcripts containing the CDS and the 7 kb long E19-derived 3’UTR do exist, we carried out a PCR reaction using 3 forward primers that are located within *BRAF* CDS and a reverse primer located in the E19 3’UTR. As shown in Additional file 1: Figure S14, we obtained a doublet of PCR bands, which are consistent with the amplification of the *BRAF*-X1 and *BRAF*-X2 isoforms. The transcription of *BRAF* CDS plus the ~7 kb long E19-derived 3’UTR was further confirmed by Northern Blot. Using a 2.3 kb long probe that recognizes *BRAF* CDS, we were able to detect a net band of ~10 kb (Fig. 4e). Finally, by transfecting A375 cells with siRNAs that recognize three different regions along the E19 3’UTR, we invariably found a downregulation of *BRAF* CDS (Fig. 4b and f). The comparison among the three *BRAF*-E19 siRNAs allowed us to establish that the most efficient is si-*BRAF*-E19-1, since it achieves a stronger *BRAF* downregulation than the other 2 siRNAs, and stronger even than the mix of the 3. Therefore we used si-*BRAF*-E19-1 only for the following studies. In Additional file 1: Figure S15, we report that the transfection of si-*BRAF*-E19-1 in 501Mel and MeWo cells produces similar results to those obtained in A375 cells.

### *BRAF* transcript variants together account for *BRAF* functions

**Fig. 5:**
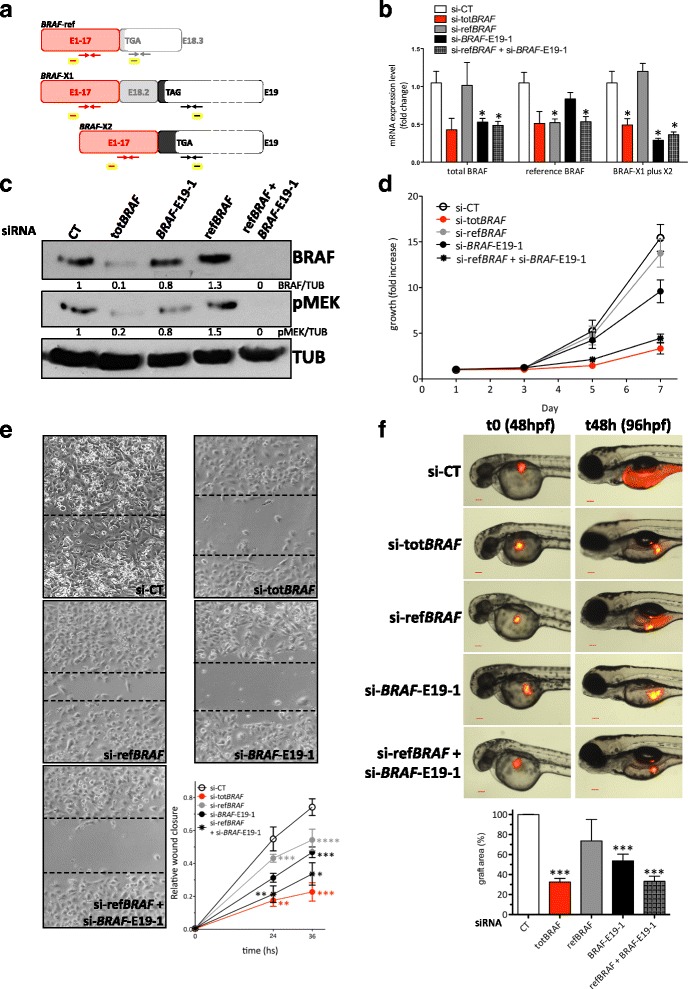
*BRAF* isoforms together account for BRAF functions in melanoma cells. **a** Cartoon summarizing the position of the primers and the siRNAs used to determine the contribution of reference, X1 and X2 isoforms to *BRAF* activities in melanoma cell lines. The primers used for real-time PCR amplification of all *BRAF* isoforms (tot*BRAF* qRT-PCR F/R), *BRAF*-ref (ref*BRAF* qRT-PCR F/R), and *BRAF*-X1 plus X2 (*BRAF*-E19-1 qRT-PCR F/R) are represented as *red*, *grey* and *black arrows*, respectively. The siRNAs used for the knock-down of the different *BRAF* isoforms are schematically represented as rectangles: *yellow* and *red*, for the knock-down of all *BRAF* isoforms (si-tot*BRAF*); *yellow* and *grey*, for the knock-down of *BRAF*-ref (si-ref*BRAF*); and *yellow* and *black* for the knock-down of *BRAF*-X1 plus X2 (si-*BRAF*-E19-1). **b** Real-time PCR detection of total *BRAF*, *BRAF*-ref, and *BRAF*-X1 plus X2 24 h after the transfection of the indicated siRNAs in A375 cells. **c** Western blot of BRAF and of its substrate pMEK 48 h after the transfection of the indicated siRNAs in A375 cells. **d** Growth curve of A375 cells after the transfection of the indicated siRNAs. **e** Wound healing assay performed using A375 cells transfected with the ndicated siRNAs. The pictures were taken 24 and 36 h after the removal of the silicone inserts. **f** Xenograft in zebrafish embryos of A375 cells stably expressing mCherry and transfected with the indicated siRNAs. The pictures are taken from 1 out of 3 independent experiments performed, all with comparable outcome. Hpf: hours post fertilization. Scale bar: 100 um. The *graphs* represent the mean ± SEM of 3 independent experiments. *p < 0.05; **p < 0.01; ***p < 0.001; ****p < 0.0001

To establish the relative contribution of the *BRAF*-ref*, BRAF*-X1, and *BRAF*-X2 isoforms to *BRAF* functions, we compared the effects obtained in A375 melanoma cells upon the knock-down of: the reference variant using si-ref*BRAF* (Fig. 5a, grey), the *BRAF-*X1 plus BRAF-X2 variants using si-*BRAF*-E19-1 (black), and all *BRAF* variants using the combo si-ref*BRAF* plus si-*BRAF*-E19-1 or si-tot*BRAF* (red). As reported in Fig. 5b, we confirmed that si-ref*BRAF* and si-*BRAF*-E19-1 are able to cause the specific knock-down of the isoform(s) they are designed to target. However, we observed that the effects of si-ref*BRAF* on total *BRAF* levels are negligible. The effects caused by si-*BRAF*-E19-1 are much stronger and comparable to those caused by si-tot*BRAF* (black bar vs red bar), while the combination with si-ref*BRAF* does not produce a further downregulation (black bar vs checked bar). These results are consistent with our findings about the higher expression levels of *BRAF*-X1 vs *BRAF*-ref mRNA, and were also confirmed in other melanoma cell lines such as 501Mel and MeWo cells (Additional file 1: Figure S16).

At the protein level we observed a consistent scenario, with BRAF levels going sensibly down only upon si-*BRAF*-E19-1 and not upon si-ref*BRAF* transfection. However, we observed that si-*BRAF*-E19-1 is not sufficient to recapitulate the effects obtained using si-tot*BRAF*, and the combination with si-ref*BRAF* is required (Fig. 5c).

Next, the consequences of the siRNA-mediated knock-down of the different variants were investigated at the functional level. To this end, we took A375 cells transfected with si-ref*BRAF*, si-*BRAF*-E19-1, si-tot*BRAF,* and si-ref*BRAF* + si-*BRAF*-E19-1 and performed three different cellular assays: two in vitro (growth curve (Fig. 5d), wound healing assay (Fig. 5e)), and one in vivo (xenograft in zebrafish embryos (Fig. 5f)). In agreement with the degree of BRAF protein downregulation observed by western blot, we discovered that the impairment in growth and motility caused by si-ref*BRAF* is very mild, while si-*BRAF*-E19-1 has much more pronounced effects. However, it is only the combination of si-ref*BRAF* and si-*BRAF*-E19-1 that can fully recapitulate the effects of si-tot*BRAF*. To explain the discrepancy between the results we obtained at the RNA and at the protein levels, we hypothesize that BRAF protein isoforms positively regulate each other, so that when they are concomitantly knocked-down the effects on total BRAF protein levels become more dramatic.

Based on these results, we conclude that *BRAF* activity in melanoma cells is fully accounted for by *BRAF*-ref and by the E19-containing *BRAF* isoforms, primarily *BRAF*-X1.

### The relative contribution of *BRAF*-ref, *BRAF*-X1, and *BRAF*-X2 transcript variants to total *BRAF* levels is maintained in the context of acquired resistance to BRAF and MEK inhibitors

We next studied whether the composition of the cocktail of *BRAF* transcript variants changes in different conditions. Specifically, we analyzed *BRAF* gene amplification [19] and *BRAF* splicing variants [20] in the context of acquired resistance to BRAF and MEKi in melanoma cells.

**Fig. 6:**
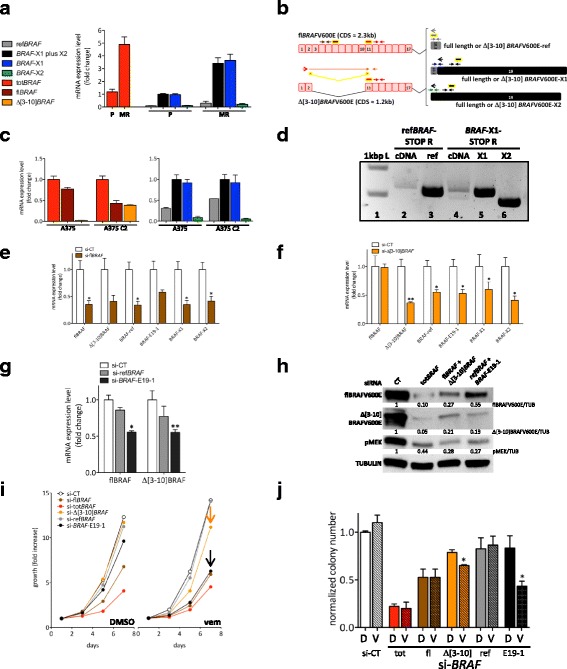
*BRAF* transcript variants in the context of acquired resistance to BRAF and MEK inhibitors. **a** Real-time PCR detection of total *BRAF* (*red*), *BRAF*-ref (*grey*), *BRAF*-X1 plus X2 (*black*), *BRAF*-X1 (*blue*), and *BRAF*-X2 (*green*) in 451Lu parental cells (P) and in 451Lu-MR resistant cells (MR). The latter show acquired resistance to BRAF and MEK inhibitors due to the focal amplification of the *BRAF* gene. **b** Cartoon summarizing the position of the primers and the siRNAs used to determine the presence and the level of the Δ[3–10] variant of *BRAF*. For details, please refer to Additional file 1: Figure S17. **c** Real-time PCR detection of total *BRAF* (*red*), full length *BRAF* (*brown*), Δ[3–10]*BRAF* (*orange*, *left panel*), *BRAF*-ref (*grey*), *BRAF*-X1 plus X2 (*black*), *BRAF*-X1 (*blue*), and *BRAF*-X2 (*green*, *right panel*) in A375 parental cells and in A375 C2 cells. The latter show acquired resistance to vemurafenib due to the presence of Δ[3–10]BRAFV600E splicing variant. **d** PCR amplification of the reference, X1, and X2 Δ[3–10]*BRAF* splicing variants from the cDNA of A375 C2 cells. Lane 1: 1 kbp ladder. Lane 2: Δ[3–10]*BRAF*-ref amplification was obtained using *BRAF*-E1/2 F primer and ref*BRAF*-STOP R primer (open *red* and *grey arrows* in **b**). Lane 3: Δ[3–10]*BRAF*-ref CDS was amplified from pMSCVHygro-Δ[3–10]BRAFV600E-ref plasmid and used as positive control. Lane 4: the amplification of Δ[3–10]*BRAF*-X1 (*upper band*) and Δ[3–10]*BRAF*-X2 (*lower band*) was obtained using *BRAF*-E1/2 F primer and *BRAF*-X1-STOP R primer (open *red* and *black arrows* in **b**). Lane 5: Δ[3–10]*BRAF*-X1 CDS was amplified from pMSCVHygro-Δ[3–10]BRAFV600E-X1 plasmid and used as positive control. Lane 6: Δ[3–10]*BRAF*-X2 CDS was amplified from pMSCVHygro-Δ[3–10]BRAFV600E-X2 plasmid and used as positive control. **e**-**f** Real-time PCR detection of full length *BRAF*, Δ[3–10]*BRAF*, *BRAF*-ref*, BRAF-*X1 plus X2, *BRAF*-X1, and *BRAF*-X2 24 h after the transfection of si-fl*BRAF* (**e**) and si-Δ[3–10]*BRAF* (**f**) in A375 C2 cells. **g** Real-time PCR detection of full length and Δ[3–10] *BRAF* 24 h after the transfection of si-ref*BRAF* and si-*BRAF*-E19-1 in A375 C2 cells. **h** Western blot of full length and Δ[3–10] BRAFV600E, as well as of pMEK 48 h after the transfection of the indicated siRNAs or siRNA mixes in A375 C2 cells. **i** Growth curve of A375 C2 cells after the transfection of the indicated siRNAs. Throughout the experiment, the cells were kept in DMSO (*left panel*) or in 2 uM vemurafenib (*right panel*). The *arrows* highlight the increased sensitivity displayed by A375 C2 cells to si-Δ[3–10]*BRAF* (*orange*) and si-*BRAF*-E19-1 (*black*), when grown in vemurafenib. The graph represents the mean only of 3 independent experiments. (**j**) Colony formation assay of A375 C2 cells after the transfection of the indicated siRNAs. Throughout the experiment, the cells were kept in DMSO (*clean bars*) or in 2 uM vemurafenib (*dashed bars*). The pictures are taken from 1 out of 3 independent experiments performed, all with comparable outcome. The *graphs* represent the mean ± SEM (or mean ± SD in **a** and **c**) of 3 independent experiments. *p < 0.05, **p < 0.01

451Lu-MR cells derive from the parental 451Lu melanoma line and show acquired resistance to both MEK and BRAF inhibitors, due to the focal amplification of *BRAF* gene [21]. As a consequence, total *BRAF* levels are increased (Fig. 6a, left). However, the composition of *BRAF* transcript variants remains quite similar (Fig. 6a, right).

A375 C2 cells derive from the parental A375 melanoma line and show acquired resistance to the BRAF inhibitor vemurafenib, due to the presence of a BRAF splicing variant that lacks exons 3 to 10 (Δ[3–10]BRAFV600E) [5]. Contrary to the parental A375 cells, where total *BRAF* levels are almost completely accounted for by full length *BRAF*, in A375 C2 cells the full length and the Δ[3–10] variants are expressed at similar levels (Fig. 6b, Additional file 1: Figure S17 and Fig. 6c, left). Nevertheless, the composition of *BRAF* transcripts remains the same: *BRAF*-X1 is more expressed than *BRAF*-ref and *BRAF*-X2, which in turn are expressed at similar levels (Fig. 6c, right). The analysis of additional vemurafenib-resistant clones and clonal populations obtained from A375 and 501Mel parental cells and carrying BRAF splicing variants produced comparable results (Additional file 1: Figure S18). These findings suggest that *BRAF* splicing variants are themselves a mixture of *BRAF*-ref, *BRAF*-X1, and *BRAF*-X2 isoforms. Indeed, when we used a *BRAF*-ref-specific reverse primer (ref*BRAF*-STOP R) to perform PCR amplification of A375 C2 cDNA, we obtained a band whose size is compatible with the reference Δ[3–10] splicing variant. However, when we used a *BRAF*-X1/X2-specific reverse primer (*BRAF*-X1-STOP R), we obtained a doublet of bands whose sizes are compatible with the X1 and X2 Δ[3–10] splicing variants (Fig. 6d). PCR results were confirmed using an siRNA that targets exon 6, causing the specific knock-down of full length *BRAF* (si-fl*BRAF*, brown in Fig. 6b) and an siRNA that spans the E2-E11 junction and therefore specifically targets the Δ[3–10] variant (si-Δ[3–10]*BRAF*, orange in Fig. 6b). As shown in Fig. 6e-f, we found that not only si-fl*BRAF*, but also si-Δ[3–10]*BRAF* can cause a decrease in the levels of all three *BRAF* isoforms (*BRAF*-ref, *BRAF*-X1, and *BRAF*-X2). Conversely, si-ref*BRAF,* and to a bigger extent si*-BRAF*-E19-1, can cause a decrease not only in full length *BRAF* levels but also in the levels of the Δ[3–10] splicing variant (Fig. 6g). In addition, we found that si-Δ[3–10]*BRAF* causes a decrease in the levels of the E19-derived 3’UTR across its entire length (Additional file 1: Figure S19a) and that, conversely, Δ[3–10]*BRAF* levels decrease upon the transfection not only of si-*BRAF*-E19-1 but also of more “downstream” siRNAs (Additional file 1: Figure S19b). We hypothesize that, analogously to full length *BRAF*, the Δ[3–10] *BRAF*-X1 and *BRAF*-X2 splicing variants have a 3’UTR that is up to 7 kb long.

Real-time results were further confirmed at the protein level: the strong decrease in both full length and Δ[3–10] BRAF protein isoforms observed with si-tot*BRAF* was also obtained by mixing si-fl*BRAF* with si-Δ[3–10]*BRAF*, as well as by mixing si-ref*BRAF* with si*-BRAF*-E19-1 (Fig. 6h).

We next aimed at assessing whether the *BRAF*-X1 is the most prevalent Δ[3–10] variant, as it happens for full length *BRAF*. Since it is known that resistant cells rely on BRAF splicing variants in order to counteract vemurafenib activity, we reasoned that if the X1 splicing variant is expressed at higher levels than the reference one, then si-*BRAF*-E19-1 should cause a much stronger increase in the sensitivity of A375 C2 cells to vemurafenib compared to si-ref*BRAF*. As shown in Fig. 6i-j, the experimental results confirmed our hypothesis. When grown in vemurafenib, A375 C2 cells are very sensitive to the knock-down of Δ[3–10]*BRAF* and *BRAF*-X1, as indicated by the decreased proliferation (i) and ability to form colonies (j) that they display upon the transfection with si-Δ[3–10]*BRAF* (orange) and si-*BRAF*-E19-1 (black).

### *BRAF*-X1 is translated into protein

**Fig. 7:**
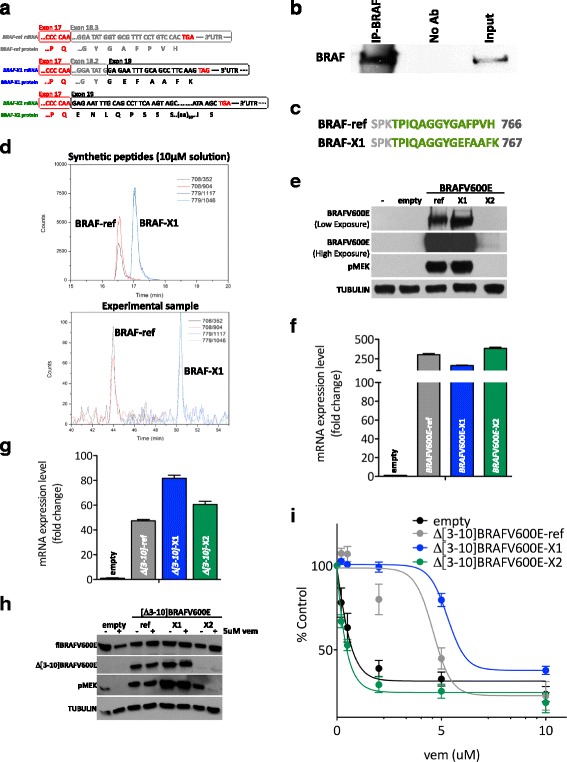
Identification and characterization of BRAF protein isoforms. **a** Schematic representation of the 3’ terminal region of reference, X1, and X2 *BRAF* mRNAs, as well as of the corresponding C-terminal regions of reference, X1, and X2 BRAF proteins. **b** Immunoprecipitation of BRAF protein in A375 cells. Endogenous BRAF was immunoprecipitated using a specific antibody that recognizes the N-terminal domain (IP-BRAF). As negative control, no antibody was used (No Ab). The basal level of BRAF in the cell lysate is shown in Input. **c** Identification by mass spectrometry of the C-terminal peptides of BRAF-ref and BRAF-X1. Immunoprecipitated BRAF was subjected to LC-MS analysis. The presence of both isoforms is revealed by the detection of isoform-specific peptides (in *green*). **d** Best transitions (BRAF-ref: 352 and 904; BRAF-X1: 1046 and 1117) of the two BRAF protein isoforms by mass spectrometry (MRM based method). **e**-**f** Upon the transient transfection of PIG-BRAFV600E-ref, X1, and X2 plasmids in HEK293T cells, western blot indicates that only reference and X1 BRAFV600E are efficiently translated and able to phosphorylate MEK, while X2 is not (**e**). This occurs in spite of the fact that according to real-time PCR for total *BRAF* levels, all 3 mRNAs are transcribed at similar levels (**f**). **g**-**i** Upon the stable infection of pMSCVHygro-Δ[3–10]BRAFV600E-ref, X1, and X2 plasmids in A375 cells, real-time PCR for total *BRAF* indicates that all 3 mRNAs are transcribed at similar levels (**g**), but western blot indicates that reference and X1 Δ[3–10]BRAFV600E are efficiently translated and able to phosphorylate MEK even in the presence of vemurafenib, while X2 is not (**h**). Consistently, only Δ[3–10]BRAFV600E-ref and -X1 are able to decrease the sensitivity of A375 cells to vemurafenib (**i**). The pictures are taken from 1 out of 3 independent experiments performed, all with comparable outcome. The *graphs* represent the mean ± SEM of 3 independent experiments

As mentioned above, *BRAF*-X1 mRNA derives from the splicing of exon 18.2 with exon 19, which contains the very last part of the CDS, the STOP codon and the 3’UTR (Fig. 1b). Not only does the *BRAF*-X1 mRNA differ from the *BRAF*-ref mRNA in its 3’UTR sequence, the BRAF-X1 protein (XP_005250102.1, GYG**EFAAFK**) also differs in its C-terminal sequence from the BRAF-ref protein (NP_004324.2, GYG**AFPVH**) (Fig. 7a and Additional file 1: Figure S20a, b). We also observed that BRAF-X1 appears to be more conserved across species than BRAF-ref (Additional file 1: Figures S21-23).

The difference between the BRAF-ref and BRAF-X1 proteins on one side and the BRAF-X2 protein (XP_005250103.1) on the other side is even more pronounced. The skipping of exon 18 and the direct splicing of exon 17 with exon 19 causes a distortion of the RNA sequence (Additional file 1: Figures S24-25) and a shift of the frame. Therefore, it results in the translation of a completely different C-terminal domain (Fig. 7a and Additional file 1: Figure S20c).

To identify BRAF protein isoforms that are translated in A375 melanoma cells, we immunoprecipitated endogenous BRAF using an antibody that recognizes the N-terminal domain (Fig. 7b). We then ran the immunoprecipitated BRAF on SDS-PAGE and subjected the corresponding band to trypsin digestion followed by mass spectrometry analysis, using an LTQ-Orbitrap XL and a 5600 TripleTOF mass spectrometers. Several peptides belonging to “human BRAF” were identified (Additional file 1: Figure S26), among them the BRAF-ref and the BRAF-X1 C-terminal peptides (Fig. 7c). Therefore we can conclude that BRAF-X1 is translated as a protein variant of BRAF. On the contrary, the BRAF-X2 protein variant was not detected. When other melanoma cell lines were tested, such as WM793B, similar results were obtained (Additional file 1: Figure S27).

We also estimated the ratio between the BRAF-ref and the BRAF-X1 proteins. To this end, we synthesized two peptides with the sequence of the C-terminal peptides identified by mass-spectrometry (see Fig. 7c, green). We then determined their spectra (Fig. 7d, upper) and confirmed that they are the same as those of the experimental peptides obtained from the immunoprecipitated BRAF (Fig. 7d, lower). The best MRM transitions proposed by SWATH analysis are 708/352 and 708/904 for the reference C-terminal peptide (TPIQAGGYGAFPVH) and 779/1046 and 779/1117 for the X1 C-terminal peptide (TPIQAGGYGEFAAFK). Since the transitions are retrieved using the synthetic peptides in equimolar concentration (10 uM, Fig. 7d, upper), we can infer that a most intense ionization of the X1 isoform occurs (response factor ref/X1 = 0.75). Furthermore, since a similar response factor (ref/X1 = 0.83) is observed in Fig. 7d lower, we can conclude that in the experimental sample the ref and X1 BRAF protein variants are expressed at similar concentration.

We next investigated whether the comparable BRAF-ref and BRAF-X1 levels, which we detected in spite of the fact that *BRAF*-X1 mRNA levels are markedly higher, are attributable to the fact that the translation of *BRAF*-ref mRNA is more efficient [22]. In order to answer to this question, we took advantage of the analysis that Obenauf and colleagues [23] performed on A375 cells and that is available at GEO (GSE64741). Specifically, we counted the paired reads that span E18.2-E18b, E18.2-E19 and E17-E19 as a measure of *BRAF*-ref, −X1, and -X2 mRNA levels, respectively, and we used them to compare total RNA samples (CT) and RNA samples obtained by TRAP (translating ribosome affinity purification). This technique enables for the enrichment of actively translated RNA molecules that are complexed with polysomes (PS). We found that *BRAF*-ref mRNA translation is in fact the most efficient (Additional file 1: Figure S28) and we propose that this might compensate for its lower stability (Additional file 1: Figure S10). It might also contribute to explain why the siRNA-mediated knock-down of *BRAF*-ref mRNA has at the protein level less pronounced effects than the knock-down of *BRAF*-X1 plus X2 mRNAs (Fig. 5c).

### The C-terminal domain of BRAF-X2 favors its degradation through the proteasome pathway

We next decided to investigate the absence of BRAF-X2 protein, as observed by mass spectrometry. By transiently transfecting BRAFV600E-ref, BRAFV600E-X1, and BRAFV600E-X2 coding sequences in HEK293T cells using the PIG-NotI plasmid, we discovered that the first two are expressed into proteins and can phosphorylate MEK (Fig. 7e, upper). However, we found that the BRAFV600E-X2 protein is expressed much less efficiently despite having similar mRNA levels to the other two variants (Fig. 7f). We also tested the Δ[3–10] splicing variants and obtained comparable results. The stable infection of pMSCVHygro-Δ[3–10]BRAFV600E-ref and -X1 in A375 cells leads to an increase in the corresponding RNA (Fig. 7g) and protein (Fig. 7h) levels. This results in a sustained activation of the MAPK pathway (MEK gets phosphorylated even in the presence of vemurafenib, Fig. 7h) and in a decreased sensitivity to the drug (Fig. 7i). On the contrary, Δ[3–10]BRAFV600E-X2 is efficiently expressed only at the RNA but not at the protein level and fails to protect A375 cells from vemurafenib activity (Fig. 7g-i). Altogether, these results suggest there is an impairment of *BRAF*-X2 expression that occurs at the post-transcriptional level.

**Fig. 8:**
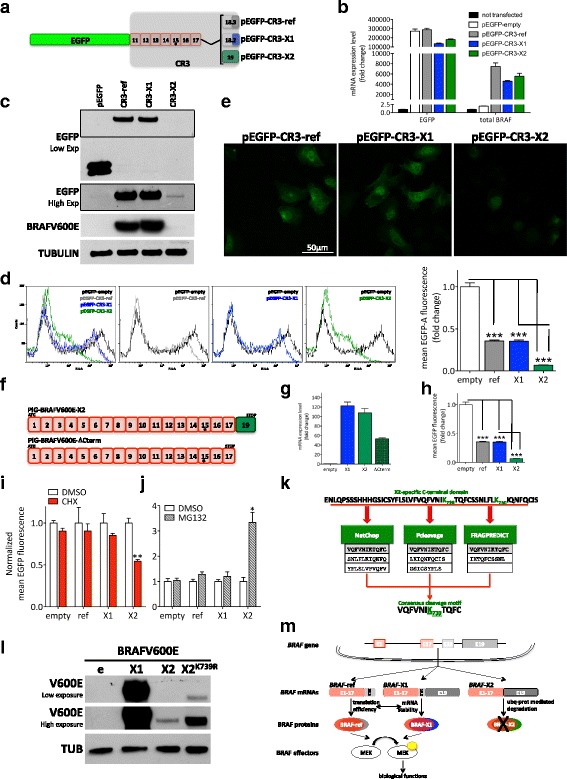
The X2 isoform displays a faster decay due to increased proteosomal-mediated degradation. **a** Schematic representation of the chimerical protein derived from the fusion of EGFP coding sequence with the CR3 domain of BRAFV600E-ref, X1, and X2 within the pEGFP-C1 plasmid. The *asterisk* indicates the presence of the V600E mutation. **b**-**e** Upon the transient transfection of pEGFP-C1 empty (pEGFP-empty), pEGFP-CR3-ref, pEGFP-CR3-X1, and pEGFP-CR3-X2 plasmids in A375 cells, real-time PCR performed with primers for EGFP and for total *BRAF* indicates that the chimerical mRNAs are all transcribed at similar levels (**b**), but western blot (**c**), flow cytometry (**d**) and confocal microscopy analysis (**e**) indicate that, when fused with CR3-X2, EGFP protein is expressed at lower levels. The *dotted box* shows a higher exposure of the anti-EGFP antibody. **f**-**h** When PIG-BRAFV600E-ΔCterm plasmid, which lacks the nucleotides encoding for the X2-specific C-terminal domain (**f**), is transiently transfected in HEK293T cells, not only *BRAF* mRNA (**g**), but also BRAF protein is detectable (**h**). e: empty PIG-NotI; X1: PIG-BRAFV600E-X1 (used as positive control); X2: PIG-BRAFV600E-X2; ΔCterm: PIG-BRAFV600E-ΔCterm. **i**-**j** Upon the transient transfection of pEGFP-empty, pEGFP-CR3-ref, pEGFP-CR3-X1, and pEGFP-CR3-X2 plasmids, A375 cells were treated with 100 ug/ml cicloheximide (CHX) (**i**) or 20 uM MG132 (**j**) for 8 h. The CHX treatment indicates that the decay rate of CR3-X2 is faster than that of CR3-ref and CR3-X1, while the MG132 treatment suggests that this is due to higher degradation rate through the ubiquitin-proteasome pathway. **k** The prediction of potential proteasomal cleavage sites using 3 different algorithms retrieves the indicated X2-specific consensus peptide. (**l**) The mutagenesis of Lys739 into a proteasome-insensitive Arg rescues the expression of the X2 isoform of BRAF protein. e: empty PIG-NotI; X1: PIG-BRAFV600E-X1 (used as positive control); X2: PIG-BRAFV600E-X2; X2^K739R^: PIG-BRAFV600E-X2 in which Lys(K)739 has been substituted with Arg(R) (AAA to AGA triplet change). **m** Cartoon that summarizes the main findings of this article (details in the text). The pictures are taken from 1 out of 3 independent experiments performed, all with comparable outcome. The *graphs* represent the mean ± SEM of 3 independent experiments. *p < 0.05, **p < 0.01, ***p < 0.001

To assess whether such impairment is dependent on the X2-specific domain, we devised two complementary strategies. First, we cloned the C-terminal CR3 region of BRAFV600E-ref, BRAFV600E-X1, and BRAFV600E-X2 in frame with EGFP coding sequence, taking advantage of the pEGFP-C1 plasmid (Fig. 8a). The pEGFP-CR3-ref, −X1, and -X2 plasmids were then transiently transfected in A375 cells. We obtained that, despite the fact that all transcripts are expressed at similar levels (Fig. 8b), the presence of the CR3-X2 domain causes a decrease in EGFP protein levels, as detected by western blot (Fig. 8c), FACS analysis (Fig. 8d), and confocal microscopy analysis (Fig. 8e). Then, we cloned the BRAFV600E-ΔCterm sequence, which is the BRAFV600E-X2 coding sequence minus the isoform-specific region that derives from the translation of exon 19 (Fig. 8f), in the PIG-NotI plasmid and used it to transfect HEK293T cells. As shown in Fig. 8g-h, we obtained a rescued expression of BRAFV600E compared to the full length BRAFV600E-X2. Therefore, we conclude that the X2-specific C-terminal domain negatively affects BRAFV600E-X2 protein levels.

To assess if the X2-specific C-terminal domain negatively affects BRAFV600E-X2 protein levels by increasing protein degradation, we transiently transfected A375 cells with the pEGFP-CR3-ref, −X1, and -X2 plasmids and treated them with 100 ug/ml cycloheximide for 8 h. In so doing, we discovered that the decay rate of the X2 isoform is faster than that of the other two isoforms (Fig. 8i). Furthermore, we discovered that the treatment of transfected A375 cells with 20 uM MG132 rescues the expression of the chimerical EGFP-CR3-X2 protein (Fig. 8j and Additional file 1: Figure S29). Therefore, we conclude that the X2 C-terminal domain causes BRAFV600E protein degradation through the ubiquitin-proteasome pathway.

Next, we aimed at predicting proteasomal cleavage sites located within the X2-specific C-terminal domain (aa710–758, green in Additional file 1: Figure S20c). To this end, we interrogated three different *in silico* algorithms: NetChop, Pcleavage, and FRAGPREDICT. The consensus analysis of the output of these three algorithms allowed us to identify the peptide VQFVNI**K**_**739**_TQFC as a very high-scoring cleavage-determining amino acid motif (Fig. 8k and Methods). Of note, when the reference-specific (aa 710–766) and the X1-specific (aa 710–767) C-terminal domains were analyzed using the same parameters, no notable proteasomal cleavage site was retrieved. This confirms the C-terminal region of BRAF-X2 is the most sensitive to proteasome-mediated degradation. To experimentally prove that the predicted cleavage-determining aminoacid motif contributes to increase the degradation of the X2 protein isoform through the ubiquitin-proteasome pathway, we mutagenized the Lysine at position 739 into a ubiquitin-insensitive Arginine and obtained the expected rescue in BRAFV600E-X2 protein expression (Fig. 8l).

## Conclusions

Our work sheds new light on *BRAF*, a crucially important gene in human cancer, by unveiling the repertoire of its mRNA and protein variants.

Using RNA-seq data obtained from more than 4800 patients and 9 cancer types, we demonstrate that *BRAF* mRNA is not a single transcript, but rather a pool of 3 transcripts (reference, X1, and X2), which differ in the length and sequence of their 3’UTR. Specifically, we show that *BRAF*-ref 3’UTR is as short as 76 nt, while the E19-derived 3’UTR that is shared by *BRAF*-X1 and *BRAF*-X2 extends for ~7 kb.

We also show that the total levels of *BRAF* mRNA are kept constant, while the levels of *BRAF*-ref on one side and those of *BRAF*-X1 plus X2 on the other side are inversely related.

In addition, we show that the ratio among the three transcripts is different in different cancer types. In melanoma in particular, the *BRAF*-X1 isoform is more expressed than the reference and the X2 isoforms, which in turn are expressed at similar levels. Remarkably, the presence and relative expression levels of the different *BRAF* isoforms are maintained when acquired resistance to vemurafenib occurs due to *BRAF* gene amplification [19] or BRAF splicing variants that lack the RAS-binding domain [20].

We demonstrate that *BRAF*-X1 mRNA, both the full length and the BRAFi-resistant Δ[3–10] splicing variant, is translated into a fully functional protein that differs from the reference one in the last few amino acids at the C-terminal domain. Together with BRAF-ref, BRAF-X1 protein accounts for *BRAF* functional activities.

We also show that the levels of the BRAF-ref and BRAF-X1 proteins are similar, despite the fact that *BRAF*-X1 mRNA is expressed at higher levels than *BRAF*-ref mRNA. We provide evidence that this might be due to a balance between mRNA stability (higher for the X1 isoform) and translation efficiency (higher for the reference isoform).

In contrast with our X1 variant experiments, we failed to detect endogenous BRAF-X2 proteins. We elucidate the underlying molecular mechanism: Lys739 in the X2-specific C-terminal domain is specifically recognized by the ubiquitin-proteasome pathway and thus causes a net impairment in protein stability.

Taken together, the results presented in this study are novel and highly relevant to basic cancer biology. They unveil that each step of *BRAF* expression (including transcription, splicing, mRNA stability, translation efficiency, and protein stability, Fig. 8m) is subjected to a very tight and sophisticated regulation that warrants further investigations. For example, we hypothesize that the 3’UTR that characterizes *BRAF*-X1 and -X2 is bound by microRNAs and RNA-binding proteins that are reasonably different from those that bind to the reference 3’UTR [24], and is therefore subjected to a different post-transcriptional regulation. We also hypothesize that, through microRNA binding, the extremely long E19-derived 3’UTR is actively involved in ceRNA networks and therefore confers coding-independent activities to *BRAF* mRNA [25]. In human cancer, amplification events can coexist with BRAFV600E mutation (www.cbioportal.org). Furthermore, the selective amplification of the BRAFV600E mutant allele has been reported as one of the mechanisms responsible for acquired resistance to BRAFi and/or MEKi [26–28]. Finally, the direct relationship that we observed between *BRAF*-X1 and *BRAF*-X2 levels is consistent with a reciprocal sponging effect. Therefore, we speculate that the unrestrained activity of the mutant BRAF protein can be further boosted by an increase in the levels of *BRAF* mRNA itself and, consequently, by an increase in the levels/activity of its oncogenic ceRNA partners.

Our results are important from a translational point of view as well.

The presence of different combinations of *BRAF* isoforms might contribute to explaining the highly heterogeneous degree of responses to BRAFi commonly observed among patients carrying different cancer types, and even among patients carrying the same cancer type [29].

Furthermore, the currently available BRAF-targeting drugs should be tested to determine whether they are equally effective against all BRAF protein variants. If this is not the case, isoform-specific drugs should be developed and used as cocktails.

Finally, we speculate that the combination of synthetic drugs that target BRAF proteins and RNA-based drugs that target *BRAF* mRNAs might result in increased efficacy, since this would concomitantly impair both the coding-dependent and the coding-independent activities of this gene.

## Methods

### Primers

All qRT-PCR and PCR primers were purchased from Eurofins Genomics. Sequences are reported in Additional file 2: Tables S3 and S4, respectively and are mapped in Additional file 3.

siRNAs were purchased from Shanghai GenePharma and their sequences are reported in Additional file 2: Table S5.

### Plasmids

#### PIG-NotI

The PIG-NotI retroviral plasmid was obtained from pMSCV-PIG plasmid (PIG, kind gift from Prof. Pandolfi, BIDMC-HMS), by adding a NotI site between the BglII and the XhoI site, so that the expanded multicloning site results composed of BglII, NotI, XhoI, and HpaI sites. PIG-NotI was used as backbone for the cloning of the plasmids listed below and as empty vector control for transient transfection/stable infection experiments.

#### PIG-BRAFV600E-ref, −X1, and -X2

BRAFV600E-ref CDS was amplified by PCR from pcDNA3.1-BRAFV600E-FLAG (kind gift from Dr. Pao, Vanderbilt University Medical Center) and cloned into the HindIII and SacI sites of pYES2 plasmid (kind gift from Dr. Galli, CNR-IFC), which was used as a cloning intermediate. The primers used for PCR amplification were the following:

- BRAF HindIII Kozak Fw 5′-CATAAGCTT**GCCACC** ATGGCGGCGCTGAGCGGTGG-3′ (ATG underlined; Kozak sequence in bold);
- BRAF SacI Rv 5′-GCAGAGCTCTCAGTGGACAGGAAACGCACCATA-3′ (STOP codon underlined).

The FLAG tag was not PCR amplified together with the CDS.

The pYES2-BRAFV600E-ref plasmid was then used as backbone for the generation of pYES2-BRAFV600E-X1 and pYES2-BRAFV600E-X2 plasmids: the X1- and X2-specific sequences were PCR amplified from A375 cDNA, using the common forward primer BglII CDS Fw 5′-GCAAGATCTCAGTAAGGTACGGAGT-3′ and the X1-specific reverse primer (BRAF X1 SacI Rv 5′-CATGAGCTCCTACTTGAAGGCTGCAAATTCTC-3′, STOP codon underlined) or the X2-specific reverse primer (BRAF X2 SacI Rv 5′-CATGAGCTCTCAGCTTATGCATTGGAAATTTTG-3′, STOP codon underlined). Subsequently, the reference specific sequence was substituted with the X1- and X2-specific sequences in the pYES2-BRAFV600E-ref plasmid, using BglII and SacI restriction sites.

At this point, BRAFV600E-ref, −X1, and -X2 CDS were PCR amplified from pYES2-BRAFV600E-ref, −X1, and -X2 plasmid using the common BRAF NotI Kozak Fw primer (5′-CATGCGGCC**GCCACC** ATGGCGGCGCTGAGCG-3′, ATG underlined, Kozak in bold) and isoform specific reverse primers:

- BRAF SalI Rv 5′-GCAGTCGACTCAGTGGACAGGAAACGCACCATATC-3′
- BRAF X1 SalI Rv 5′-GCAGTCGACCTACTTGAAGGCTGCAAATTCTC-3′
- BRAF X2 SalI Rv 5′-GCAGTCGACTCAGCTTATGCATTGGAAATTTTG-3′

(STOP codon underlined).

Finally, the CDS were cloned in PIG-NotI using the NotI and XhoI restriction sites.

#### PIG-BRAFV600E-ΔCterm

The BRAFV600E CDS lacking the X2-specific triplets was PCR amplified from PIG-BRAFV600E-X2 plasmid, using BRAF NotI Kozak Fw primer and the BRAF X2deltaCterm SalI Rv primer (5′-CATGTCGACTCATTGGGGAAAGAGTGGTCTCTCAT-3′, STOP codon underlined). It was then cloned in PIG-NotI using the NotI and XhoI restriction sites.

#### PIG-BRAFV600E-X2^K739R^

The BRAFV600E-X2 CDS that is mutagenized at the 739 aa position was PCR amplified from pYES2-BRAFV600E-X2^K739R^ plasmid (see “Mutagenesis” below), using the BRAF NotI Kozak Fw primer and the BRAF X2 SalI Rv primer, then cloned in PIG-NotI using the NotI and XhoI restriction sites.

#### pMSCVHygro-NotI

The pMSCVHygro retroviral plasmid was obtained from pMSCVHygro plasmid (kind gift from Prof. Pandolfi, BIDMC-HMS), by adding a NotI site between the BglII and the XhoI site, so that the expanded multicloning site results composed of BglII, NotI, XhoI, and HpaI sites. pMSCVHygro-NotI was used as backbone for the cloning of the plasmids listed below and as empty vector control for transient transfection/stable infection experiments.

#### pMSCVHygro-Δ[3–10]BRAFV600E-ref, −X1 and -X2

These plasmids were obtained following the same strategy already described for PIG-BRAFV600E-ref, −X1, and -X2. However, the starting point was the Δ[3–10]*BRAF*V600E-ref cDNA that was amplified by PCR from A375 C2 cDNA using the BRAF HindIII Kozak Fw and the BRAF SacI Rv primers mentioned above.

#### pEGFP-CR3-ref, −X1 and -X2

These plasmids were obtained by cloning the CR3 domain of reference, X1, and X2 BRAFV600E (from exon 11 to the exon containing the STOP codon) in frame with EGFP in the pEGFP-C1 plasmid (Clontech). The CR3 domains were obtained by PCR amplification from PIG-BRAFV600E-ref, −X1, and -X2 plasmids, using a common forward primer BRAFCR3 HindIII Fw (5′-CTCAAGCTTTGAAAACACTTGGTAGACGGGAC-3′) and isoform-specifc reverse primers (BRAF SalI Rv, BRAF X1 SalI Rv, BRAF X2 SalI Rv, see above for the sequences).

### Mutagenesis

The *BRAF*V600E-X2^K739R^ CDS was obtained using QuikChange Lightning Site-Directed Mutagenesis Kit (Agilent). 100 ng of pYES2-BRAFV600E-X2 plasmid were used as a template, along with the primers for mutagenesis listed in Additional file 2: Table S4, following the manufacturer’s instructions.

### Cell Culturing

Cells were grown at 37 °C in a humidified atmosphere with 5% CO_2_ and they were routinely tested for mycoplasma contamination. The cell lines derived from solid tumors were cultured and fingerprinted as reported in ref [16].

The resistant clones and clonal populations were obtained by prolonged exposure of A375 and 501Mel parental cells to 2 uM vemurafenib as reported in [5].

HEK293T cells were cultured in DMEM low glucose (Euroclone) supplemented with 10% fetal bovine serum (FBS, Euroclone), 1% glutamine (Sigma-Aldrich) and 1% Penicillin/Streptomycin (Euroclone).

### RNA extraction and retrotranscription

RNA was extracted using QIAzol (Qiagen), following the manufacturer’s instructions. Before retrotranscription, 1ug of total RNA was subjected to DNAse treatment using DNaseI amplification grade (Invitrogen), following the manufacturer’s protocol. 0.25ug of DNAse-treated RNA were retrotranscribed on a S1000 Thermal Cycler (Bio-Rad) using iScript cDNA Synthesis Kit (Bio-Rad).

### PCR

To determine the length of the E19-derived 3’UTR, the PCR reactions were performed using the PCR Master Mix (Thermo Fisher Scientific) in a S1000 Thermal Cycler. 1 ul of cDNA or 10 ng of gDNA were used as templates. The primers are listed in Additional file 2: Table S4 (*BRAF*-E19-1/2/3/4 F/R). The reaction condition were: 98 °C 10 s, (98 °C 1 s, 57 °C 5 s, 72 °C 55 s)× 35 cycles, 72 °C 1 min. The “chained PCR” were performed using Phusion Flash High Fidelity Master mix (Thermo Fisher Scientific) with the following reaction conditions: 98 °C 10 s, (98 °C 1 s, 55 °C 5 s, 72 °C 45 s)× 35 cycles, 72 °C 7 min).

To determine the expression of the NE6 exon, 1 ul of cDNA obtained from A375 cells was used as template in PCR reactions, using Phusion Flash High Fidelity Master mix (Thermo Fisher Scientific). The primers used were: *BRAF*-E1/2 F, *BRAF*-E3 qRT-PCR F, *BRAF*-NE6p qRT-PCR F, *BRAF-*E4a qRT-PCR R, *BRAF*-E4b R, and *BRAF*-NE6p qRT-PCR R (Additional file 2: Tables S3 and S4). The PCR conditions were: 98 °C 1 s, (98 °C 1 s, 57 °C 5 s, 72 °C 15 s)× 35 cycles, 72 °C 1 min.

To determine the expression of Δ[3–10] splicing variant in its reference, X1, and X2 isoforms, 1ul of cDNA obtained from A375 C2 cells was used as template, while 10 ng of pMSCVHygro-Δ[3–10]BRAFV600E-ref, X1, and X2 plasmids were used as positive controls. The reaction condition using Phusion Flash High Fidelity Master mix and primers *BRAF*-E1/2 F, ref*BRAF*-STOP R, *BRAF*-X1-STOP R (Additional file 2: Table S4) were: 98 °C 10 s, (98 °C 1 s, 57 °C 5 s, 72 °C 35 s)× 35 cycles, 72 °C 1 min.

### Real-time PCR

For real-time PCR (qRT-PCR) reactions, 1 ul of diluited cDNA (1:5), the SsoAdvanced Universal Supermix (Bio-Rad) and appropriate primers (Additional file 2: Table S3) were used in a CFX96 Real-Time System (Bio-Rad). The reaction conditions were the following: 98 °C 30 s, (98 °C 3 s, 58 °C 20 s, 72 °C 10 s)× 40 cycles. In order to confirm the specificity of the reaction, a melting curve was performed after each PCR (from 65 °C to 95 °C with an increase of temperature of 0.5 °C/s). All reactions were performed in duplicate, and the average of the two C_t_ values was used to calculate the expression of the different transcripts by the “2^-ΔΔCt^” method, using the geometrical square mean of three housekeeping genes as a reference.

### siRNA transfection

2×10^5^ A375 or 2,5×10^5^ MeWo and 501 Mel cells/6well plate were seeded. The day after, 6ul of 20 uM siRNA stock solution (see Additional file 2: Table S5 for siRNA sequences) per well were added to 250 ul of OptiMEM I® (Invitrogen), while 10 ul of 1 mg/ml LIPOFECTAMINE 2000™ (Invitrogen) were added to additional 250 ul of OptiMEM I®. These two solutions were then combined together and the siRNA-LIPOFECTAMINE 2000™ complexes were allowed to form for 20 min at room temperature. In the meantime, the medium from each well was aspirated and replaced with 1.5 ml of fresh OptiMEM I®. The OptiMEM I®/siRNA/LIPOFECTAMINE 2000™ mixture was then added to the wells, let stand for 6 h and replaced with complete medium for 24 h (for qRT-PCR analyses) or 48 h (for western blot analyses). In alternative, at the end of the 6 h, the cells were trypsinized and used for cellular assays.

### Plasmid transfection

The transfection of plasmids in HEK293T cells was performed using PEI. 5×10^5^ cells/6well plate were seeded. In the afternoon of the day after, 167 ul of DMEM low glucose were mixed with 4ug of plasmidic DNA and 7.5 ul of 0.02 mM PEI (Sigma-Aldrich). The mixture was then left at room temperature for 20 min. Meanwhile, the cell medium was replaced with 833 ul of fresh DMEM low glucose containing 2%FBS. The DNA/PEI/DMEM mixture was then added to the wells and the next morning was replaced with fresh complete DMEM low glucose medium. Cell pellets were collected after 24 additional hours.

The transfection of plasmids in A375 cells was performed using LIPOFECTAMINE 2000™ (Invitrogen). 5×10^4^ A375 cells/24well plate were seeded and 24 h later they were transfected with 0.8 ug of plasmidic DNA and 2 ul of 1 mg/ml LIPOFECTAMINE 2000™, following the manufacturer’s protocol.

### Stable infection

The stable infections of retroviral plasmids were carried out as described in [30].

### Proliferation assay

6 h after the transfection mix was added, A375 or A375 C2 siRNA-transfected cells were seeded at 3000 cells/well in 12well plates (3 wells per condition per time point). Each time point (1, 3, 5, and 7 days post transfection) was fixed with 4% paraformaldehyde (PFA) for 10 min at room temperature. All time points were then stained together using 0.5 ml/well of crystal violet solution (0.1% crystal violet, 20% methanol) for 15 min at room temperature on a rocking plate. After the excess crystal violet solution was removed, the plates were washed with tap water and dried. Then, they were de-stained using a 10% acetic acid solution. Finally, the absorbance of each well was read in duplicate at 590 nm. Each sample was normalized on its first time point (day 1) and the results of three independent experiments were averaged for statistical analyses.

### Clonogenicity assay

6 h after the transfection mix was added, 2×10^2^ siRNA-transfected A375 C2 cells were seeded in 6 cm plates in triplicate and treated with vehicle (DMSO) or 2 uM vemurafenib. After 8 days, cells were fixed and stained with a 0.1% crystal violet, 4% formaldehyde solution. The number of colonies of the si-CT transfected and DMSO-treated cells was used as normalizer and the results of three independent experiments were averaged for statistical analyses.

### Migration assay

6 h after the transfection mix was added, 1.5×10^4^ siRNA-transfected A375 cells were seeded on a plate using silicone inserts (Culture-Insert Family, IBIDI). 24 h later (t0), when the confluency was ~70–80%, the inserts were removed and the quality of the covered surface was evaluated. Then, the cell-free gaps were monitored at different time points after insert removal, using 10× and 20× objective lens. Images were captured using Leica DM IL LED microscope. The measure of cell-free gaps was taken with Image J software (http://rsb.info.nih.gov). The migratory rate was determined as a relative percentage of gap closure compared to the t0 area. For each condition, ‘wounds’ from three independent experiments were measured.

### Western blot analysis

Cells were harvested and protein were extracted with 5 mM Tris pH8, 1% TritonX 100, 0.25% Sodium Deoxycholate, 10% proteinase inhibitor, 2% PMSF, 0.5% Ortovanadate. The samples were then heated at 95 °C for 5 min, separated on 10% SDS-polyacrylamide gels (Mini-PROTEAN Precast gel, Bio-Rad) and electrotransferred to polyvinylidene difluoride (PVDF) membranes using Trans-Blot Turbo system (Bio-Rad).

Membranes were blocked at room temperature for 1 h using 3% milk in TBST for the detection of BRAFV600E, BRAF, α-TUBULIN and EGFP or 3% BSA in TBST for the detection of pMEK. They were then incubated overnight at 4 °C with the following primary antibodies:

- anti-BRAF (F-7, #5284, Santa Cruz Biotechnology; mouse monoclonal antibody, dilution 1:1000 in 5% milk in TBST);
- anti-human BRAFV600E (VE1, #E19290, Spring Bioscience; mouse monoclonal antibody, dilution 1:400 in 1% milk in TBST);
- anti-pMEK (#9154, Cell Signaling; rabbit monoclonal antibody, dilution 1:1000 in 3% BSA in TBST);
- anti-α-TUBULIN (#T9026, Sigma-Aldrich; mouse monoclonal antibody, dilution 1:20000 in 5% milk in TBST);
- anti-EGFP (#A111-22, Molecular Probes, rabbit polyclonal antibody, dilution 1:2000 in 5% milk in TBST).

According to the manufacturers’ indications, all the primary antibodies used have been tested for their ability to recognize the relevant human proteins.

The detection of primary antibodies was performed using alkaline phosphatase-conjugated secondary antibodies and enhanced chemiluminescence reagents (GE Healthcare, Life Sciences, Milano, Italy).

### FACS analysis

The day after transfection, A375 cells were treated with 20 uM MG132 (Sigma-Aldrich) or 100 ug/ml cycloheximide (CHX, Sigma-Aldrich) or both in combination for 8 h. At the end of the treatment, cells were collected and washed once with PBS. The mean fluorescence of EGFP was then measured by flow cytometry using Accuri C6 (BD Biosciences).

### Confocal microscopy analysis

5 × 10^4^ A375 cells were seeded on circular 12 mm coverslips and transfected with pEGFP-C1 or pEGFP-CR3-ref/X1/X2 plasmids as reported in “Plasmid transfection”. The day after, the cells were washed twice with PBS and fixed with 4% formaldehyde for 10 min at room temperature. The coverslips with fixed cells were then mounted on microscope glass using Vectashield® and EGFP was visualized by confocal microscopy (inverted confocal laser scanning microscopy Leica TCS SP8 microscope).

### Actinomycin D assay

2.5 × 10^5^ cells were seeded in 6well plates. The day after, the cells seeded in 1 well were collected and used as t0, while the others were treated with 10 ug/ml actinomycin D (Sigma-Aldrich) for 2/4/8/12/24 h. Total RNA was then extracted and qRT-PCR performed as described above.

### In vitro *BRAF* transcription

The in vitro transcription of the [a^32^P]UTP-labeled antisense *BRAF* probe was performed using T7 RNA polymerase on a linearized plasmid containing *BRAF* CDS (2.3 kbp). The MEGAshortscript™ T7 Transcription kit (Thermo Fisher Scientific) was used.

### Northern blot

A total of 30 ug of each RNA sample was resolved in a 1% formaldehyde-agarose gel, transferred to a GeneScreen Plus membrane (Perkin-Elmer, Waltham, MA), hybridized with a ^32^P-labeled antisense RNA probe for *BRAF* CDS at 45° on ULTRAhyb® Ultrasensitive Hybridization Buffer (Thermo Fisher Scientific), washed and, finally, detected and quantified with a Cyclone Phosphoimager (Perkin-Elmer).

### Immunoprecipitation

A375 cells were lysed on ice for 1 h by incubating them in Pierce Lysis buffer (25 mM Tris, 0.15 M NaCl, 1 mM EDTA, 1% NP40, 5% glycerol, pH 7.4) supplemented with protease inhibitors. They were then subjected to sonication 3 times for 30 s and centrifuged at 16000 g for 30 min at 4 °C in order to remove cellular debris. Protein concentration was determined by bicinchoninic assay (#23223 and #23224, Pierce). Subsequently, 1 mg of proteins in a final volume of 500 ul were mixed with 10 ug of BRAF antibody (F-7, #5284, Santa Cruz Biotechnology, see above) and 25 ul of Protein G magnetic beads (Pierce) overnight at 4 °C. The immunoprecipitate was washed once with water and boiled in loading buffer in order to release the BRAF-antibody complexes from the beads. Western blot was performed using again F7 BRAF antibody (dilution 1:500 in 5% milk in TBST) and overnight incubation.

### In-gel tryptic digestion and LC-mass spectrometry

Immunoprecipitates were separated by SDS-PAGE using 10% polyacrylamide gel. Gel was subject to coomassie staining and the bands corresponding to BRAF size were excised. For each gel lane, a single band was excised, dehydrated with acetonitrile, and dried under vacuum. Gel was incubated with 10 mM dithiothreitol (Fluka) in 25 mM NH_4_HCO_3_ for 45 min at 56 °C. After removing the reducing solution, alkylation was performed with 55 mM iodoacetamide (Sigma-Aldrich) dissolved in 25 mM NH_4_HCO_3_ for 30 min with constant shaking, at room temperature and in the dark. In-gel protein digestion was performed overnight by treating the samples with 5 ng/ul sequencing-grade modified trypsin (Promega) in 25 mM NH_4_HCO_3_ at 37 °C. Peptides were extracted from the gel with 50% acetonitrile/0.1% formic acid (v/v) and analyzed with a LTQ-Orbitrap XL mass spectrometer (Thermo Fisher Scientific) coupled with a nano-HPLC Ultimate 3000 (Dionex-Thermo Fisher Scientific). Peptides were loaded onto a homemade pico-frit column packed with C18 material (Aeris Peptide 3.6 um XB-C18; Phenomenex) and separated using a linear gradient of acetonitrile/0.1% formic acid from 3 to 40% in 20 min at a flow rate of 250 nl/min. Spray voltage was set to 1.2 kV with an ion source capillary temperature of 200 °C. The instrument was operating in data-dependent mode with a top-ten acquisition method (a full scan at 60,000 resolution on the Orbitrap followed by MS/MS fragmentation in the linear trap of the ten most intense ions).

#### Analysis of LC-mass spectrometry data

The acquired raw data files were processed using Proteome Discoverer 1.4 software (Thermo Fisher Scientific) coupled with a Mascot server (version 2.2.4; Matrix Science). Protein identification was performed against the Uniprot Human protein database (release April 2015; 90411 sequences) modified in-house with the addition of three FASTA sequences from NCBI database: Serine/threonine-protein kinase BRAF (ref. NP_004324.2); Predicted: Serine/threonine-protein kinase BRAF isoform X1 (ref. XP_005250102.1); predicted: Serine/threonine-protein kinase BRAF isoform X2 (ref. XP_005250103.1).

Enzyme specificity was set to trypsin, and a maximum of one missed cleavage was allowed. The precursor and fragment mass tolerances were set to 10 ppm and 0.6 Da, respectively. Carbamidomethylation of Cys was set as a static modification, whereas oxidation of Met was set as variable modification. Percolator (Wright et al., 2012) was used to assess peptide and protein identification confidence: only proteins identified with at least 2 unique peptides with high confidence (q ≤ 0.01) were considered as positive hits. Proteins were grouped into protein families according to the principle of maximum parsimony.

### Protein preparation for subsequent LC-MS/MS SWATH

The proteins present in the gel slices from immunoprecipitates were reduced with dithiothreitol 5 mM at 80 °C for 30 min and alkylated using iodoacetamide 10 mM at 37 °C for 20 min (both in 50 mM NH_4_HCO_3_). Digestion was performed by incubating the proteins with 10ug/ul trypsin (#11418033001, Roche) in 25 mM NH_4_HCO_3_ at 37 °C overnight. The peptide solution was recovered by sonication and centrifugation at 16000 g and then loaded on a C18 column. Once eluted from the column, the peptides were diluted in a 2% Acetonitrile, 0.1% Formic acid buffer and filtered with a 0.22 um filter.

#### Analysis of LC-MS/MS SWATH data

Peptides were subject to chromatographic separation using a nano-HPLC system (Eksigent, ABSciex). The samples were pre-concentrated in a pre-column cartridge by the loading pump (PepMap-100 C18 um, 100 Å, 0.1 × 20 mm, Thermo Fisher Scientific) and then separated in a C18 PepMap-100 column (3 um, 75 um × 250 mm, Thermo Fisher Scientific) at 300 nl/min flow rate. Each run was done with eluent A (ultrapure water, 0.1% Formic acid) with 60 min linear gradient from 5 to 40% of eluent B (Acetonitrile, 0.1% Formic acid) followed by a purge step of 10 min and a re-equilibration step of 20 min. Eluted peptides were directly processed by 5600 TripleTOF™ mass spectrometer (ABSciex) equipped with a Duo Spray™ ion source (ABSciex). Information dependent acquisition (IDA) analysis was performed acquiring survey scans in 250 ms and collecting 25 products ion scans if a threshold of 125 counts per second was exceeded. For each scan four time bins were summed at a pulser frequency value of 11 kHz through monitoring of the 40 GHz multichannel TDC detector with 4-anode/channel detection. Dynamic exclusion was set for 1/2 of peak width (~8 s) and the precursor was refreshed off the exclusion list. We performed three runs for each sample. MS/MS data were processed with ProteinPilot software (ABSciex), using the Paragon and Pro Group Algorithms against the database containing all the human protein sequences from NCBI Reference Sequences. The false discovery rate (FDR) was analyzed by the integrated tools in ProteinPilot software with a set confidence level of 95%. Data were also acquired using the new Sequential Window Acquisition of all Theoretical Mass Spectra (SWATH™) method for shotgun data independent MRM quantification. We performed three runs for each sample. The SWATH MS spectral library was generated by ProteinPilot Software. PeakView4.5 Software (ABSciex) with MS/MS(ALL) with SWATH™ Acquisition MicroApp 2.0 and MarkerViewTM (ABSciex) was used for label free statistical comparative analysis. Peptides from top score proteins were selected for retention time alignment with a processing settings of 7 peptides per protein, 7 transitions per peptide, 95% peptide confidence, 5% FDR, XIC (Extracted-Ion Chromatogram) extraction window of 10 min, width 50 ppm and 0.1 Da. Global normalization of profiles based on total protein content was applied.

### Peptide synthesis and purification

The two peptides TPIQAGGYGAFPVH (C-terminal of BRAF-ref) and TPIQAGGYGEFAAFK (C-terminal of BRAF-X1) were prepared by solid-phase synthesis using Fmoc chemistry on an automatic Liberty Blue Peptide Synthesizer with an integrated microwave system (CEM). Standard couplings suggested by the producer were used thorough the synthesis. The resin was Rink-amide, loading 0.46 mmol/g (Bachem). The deprotected peptide was precipitated with cold diethyl ether after removing in vacuo most of the cleavage cocktail. In turn, the cleavage cocktail was composed of: TFA/Tri(isopropylsilane)/H2O/Ethanedithiol/thioanisole 93/2/2/2/1; cleavage was performed according to manufacturer conditions (30 min at 30 °C under microwave irradiation). HPLC analyses and purifications were performed on a Dionex Ultimate 3000 HPLC system or on a Shimadzu Nexera HPLC system. Crude peptides were purified by RP-HPLC on a Jupiter Proteo 90 Å column (4 uM, 250 × 10 mm; Phenomenex) using water:TFA 100:0.01 v/v (eluent A) and acetonitrile:water:TFA 95:5:0.01 v/v (eluent B) as mobile phase. The identity of the purified product was confirmed by electrospray mass spectroscopy, using an API3200QTRAP Hybrid Triple Quadrupole/Linear Ion Trap (ABSciex).

### Mass spectrometry characterization of synthetic peptides

Synthetic peptides were analysed by LC-MSMS using a microHPLC (Eksigent LC-300) interfaced with 5500 QTRAP (ABSciex). 5 ul of a 10 uM solution of each peptide was injected in a Jupiter 4u Proteo 90A chromatographic column (250 × 0.3 mm, Phenomenex) at a flow rate of 5 ul/min. Runs were performed with eluent A (ultrapure water, 0.1% Formic acid) under 10 min linear gradient from 5 to 95% of eluent B (Acetonitrile, 0.1% Formic acid) followed by 5 min of re-equilibration step. Peptides eluted from chromatography were directly processed using 5500 QTRAP™ mass spectrometer (ABSciex) equipped with an ESI ion source (ABSciex). Precursor Ions (MS spectra) and MSMS fragmentations (Product ions) were obtained. For C-terminal peptide of BRAF-ref, the m/z principal ion was m/z 708.00 (M + 2H^+^) and the Q3 selected ions were 904.44, 352.92, and 440.76, while for C terminal peptide of BRAF-X1 Q1 the m/z principal ion was m/z 779.04 (M + 2H^+^) and Q3 selected ions were 1046.49, 1117.53, and 974.52.

The best MRM transitions proposed by SWATH analysis were 708/352 and 708/904 for the C-terminal peptide of BRAF-ref and 779/1046 and 779/1117 for the C-terminal peptide from BRAF-X1. These ions were chosen for peptide quantitation analysis and for determining peptide area ratio at known concentration (10 uM solutions).

### Xenograft in zebrafish embryos

24 h after siRNA transfection, mcherry-A375 cells were prepared by mixing 10^6^ cells with 2ul of MATRIGEL (Cultrex Basement Membrane Extract, PathClear) and injected into zebrafish embryos of the wt AB line (RRID:ZIRC_ZL1, kind gift from Prof. Andreazzoli, Dipartimento di Biologia, Università di Pisa, Italy). The AB fishes were raised in the zebrafish facility at CNR-IFC and inbred. The protocol followed for the injection and the subsequent analyses was the one described in ref [31]. Briefly, 48 hpf embryos were anesthetizes with 0.04 mg tricaine (#A-5040, Sigma-Aldrich) and grafted in the perivitelline space with 500 cells in 1 nl. The fluorescence imaging was carried out 2 days after the injection using the Nikon Eclips E600 microscope. The embryos were carefully set down on glass slides embedded with 1% low melting agarose. The acquisitions were conducted with CoolSnap-CF camera using NIS-Elements software version 2.0. ImageJ software (http://rsb.info.nih.gov) was used to analyze the tumor area. The rcmdr package of R software was used for statistical analysis (http://www.rcommander.com/). 15–25 embryos were injected per experimental condition and the experiment was repeated 3 times.

### Statistical analyses

Data were analyzed with unpaired and two-tailed t test (GraphPad Prism, GraphPad Software Inc.). Values of *p* < 0.05 were considered statistically significant (*p < 0.05, **p < 0.01, *** < 0.001, ****p < 0.0001).

### Analysis of *BRAF* transcript variants

#### Data download and read mapping

RNA-seq paired-end raw reads of nine cancers were downloaded from the TCGA database. Raw reads were then mapped on human genome version GRCh37.75, using tophat2 on Pegasus cluster in the Center for Computational Science at University of Miami.

#### Count of reads on exons

The reads mapping on each exon of *BRAF* in each individual were extracted from alignment files using an in-house program. Only completely mapped reads were kept for the following analysis.

#### Count of exon-spanning reads

The exon-spanning reads were identified as reads of the same pair that are mapped one on an exon and the other on another exon, with the distance in between falling in a reasonable range as inferred by all read pairs.

#### Comparison among exons and transcripts

The comparison among exons and transcripts regarding read counts was conducted using R. The reads on each exon were normalized dividing them by the total number of reads mapped on all exons of each individual and then multiplying them by a 10^7^ factor, which brings the normalized values into a suitable range for comparison. The normalized values falling outside 95% quantile were discarded as outliers.

### Analysis of the secondary structure of *BRAF*-X1 and *BRAF*-X2 mRNAs

The prediction of the most stable secondary structure of *BRAF*-X1 mRNA (XM_005250045.1, 9464 nt starting from the ATG) and *BRAF*-X2 mRNA (XM_005250046.1, 9310 nt starting from the ATG) was performed using UNAfold program [32], version 3.8. Among all the possible ones, the secondary structure showing the lowest energy of pairing (dG) was taken for each of the 2 molecules. These structures were then compared using a dot plot. The conversion was made taking advantage of the Python interactive visualization library Bokeh, version 0.11.1 (http://bokeh.pydata.org/en/latest/).

### Prediction of potential proteasomal cleavage sites

Three different algorithms have been used. NetChop 3.1 stand-alone software package [33] is a neuronal network method trained with a database consisting of 1260 publicly available C-terminal cleavage site of proteasome ligands (3.0 C-ter option). To select the most probable cleavage-determining amino acid motifs, we performed our *in silico* analyses using the very high 0.9 threshold. Pcleavage is a Support Vector Machine (SVM) method for the prediction of 20S proteasome cleavage sites based on 172 fragments generated by 184 distinct cleavage sites [34]. Also in this case, a very high stringency threshold (0.2) was used in our analysis. Finally, FRAGPREDICT combines the two strategies: it predicts potential proteasomal cleavage sites that are used as input for the selection of major proteolytic fragments, applying the kinetic model of the 20S proteasome [35, 36]. Also in this case a stringent threshold value was used (0.9). The final output was obtained through a consensus analysis, by considering the fragments selected by all three algorithms.

## Additional files

Additional file 1: **Figure S1.** Schematic representation of the exons belonging to the different variants of human *BRAF* reported in NCBI and Ensembl. **Figure S2.** Scan of exon 18 of *BRAF* in 8 cancer types. **Figure S3.** Count of the reads mapping to all *BRAF* exons, E19 included. **Figure S4.** Count of reads mapping to all *BRAF* exons (w/o E19 on the left and w E19 on the right). **Figure S5.** Count of E13-E16 exon-spanning reads. **Figure S6.** *BRAF*-004 transcript variant is expressed but it is truncated. **Figure S7.** Position of the primers/siRNAs used to detect/downregulate *BRAF*-ref, *BRAF*-X1, and *BRAF*-X2. **Figure S8.** Real-time PCR detection of *BRAF*-ref, *BRAF*-X1 plus X2, *BRAF*-X1, and *BRAF*-X2. **Figure S9.** Box plot of the reads that span E17-E18.2, E18.2-E18b, E18.2-E19, and E17-E19. **Figure S10.** Stability of reference and X1/X2 *BRAF* mRNA. **Figure S11.** Correlation among the expression levels of the different *BRAF* isoforms in breast cancer, head and neck cancer, lung SCC, AML, and DLBCL. **Figure S12.** Lack of association of reference, X1, and X2 *BRAF* levels with age (left panels), gender (middle panels), and stage at diagnosis in primary and metastatic melanoma patients. **Figure S13.** Lack of association of age (left panels), gender (middle panels), and stage at diagnosis (right panels) with the ratio between *BRAF*-X1 and *BRAF*-ref levels (upper panels, red) and with the ratio between *BRAF*-X2 and *BRAF*-ref levels (lower panels, blue) in primary and metastatic melanoma patients. **Figure S14.** Tools for the detection of *BRAF* CDS plus the E19-derived 3’UTR. **Figure S15.** Length of *BRAF*-X1 and *BRAF*-X2 3’UTR in melanoma. **Figure S16.** siRNA-mediated downregulation of *BRAF* isoforms in melanoma cells. **Figure S17.** Cartoon summarizing the position of the primers and the siRNAs used to determine the levels and the identity of the Δ[3–10] splicing variant of *BRAF*. **Figure S18.** Real-time PCR detection of *BRAF*-ref (grey) and *BRAF*-X1 plus X2 (black) in vemurafenib-resistant clones and clonal populations. **Figure S19.** The 3’UTR of X1 and X2 Δ[3–10]*BRAF* splicing variant is up to 7 kb long. **Figure S20.** Sequence of reference, X1, and X2 BRAF proteins. **Figure S21.** Alignment between the sequence of human BRAF-ref (NP_004324.2, left) or BRAF-X1 (XP_005250102.1, right) and mouse Braf-ref (NP_647455.3). **Figure S22.** Alignment between the sequence of human BRAF-ref (NP_004324.2, left) or BRAF-X1 (XP_005250102.1, right) and rat Braf-X1 (XP_001070228.2). **Figure S23.** Alignment between the sequence of human BRAF-ref (NP_004324.2, left) or BRAF-X1 (XP_005250102.1, right) and pig Braf-X1 (XP_005654324.1). **Figure S24.** Dot plot of the secondary structure of *BRAF*-X1 (blue) and *BRAF*-X2 (green) mRNA sequences. **Figure S25.** Enlargement of box1 as reported in Figure S24. **Figure S26.** BRAF peptides identified by an LTQ-Orbitrap XL mass spectrometer (Thermo Fisher Scientific, a) and by a 5600 TripleTOF mass spectrometer (ABSciex, b) in A375 melanoma cells. **Figure S27.** BRAF peptides identified by LTQ-Orbitrap XL mass spectrometer (Thermo Fisher Scientific) in WM793B melanoma cells. **Figure S28.** Translation efficiency of reference, X1 and X2 *BRAF* mRNAs in A375 cells. **Figure S29.** BRAF-X2 protein displays a faster decay due to increased proteosomal-mediated degradation. (PDF 14500 kb)

Additional file 2: **Table S1.** Coordinates of the exons of human *BRAF* analyzed in this study. **Table S2.** List of the human tumor samples used in this study. **Table S3.** Real-time PCR primers. **Table S4.** PCR primers. **Table S5.** siRNA sequences. (PDF 172 kb)

Additional file 3: Primers and siRNAs location along *BRAF*-ref, *BRAF*-X1, and *BRAF*-X2 mRNAs. (PDF 67 kb)
