# Supplementary material for: The landscape of BRAF transcript and protein variants in human cancer
Source: Mol Cancer. 2017 Apr 28;16:85. doi: 10.1186/s12943-017-0645-4 (PMC5410044; doi:10.1186/s12943-017-0645-4)
Supplement: Supplementary file 1 — Figure S1. Schematic representation of the exons belonging to the different variants of human BRAF reported in NCBI and Ensembl. Figure S2. Scan of exon 18 of BRAF in 8 cancer types. Figure S3. Count of the reads mapping to all BRAF exons, E19 included. Figure S4. Count of reads mapping to all BRAF exons (w/o E19 on the left and w E19 on the right). Figure S5. Count of E13-E16 exon-spanning reads. Figure S6. BRAF-004 transcript variant is expressed but it is truncated. Figure S7. Position of the primers/siRNAs used to detect/downregulate BRAF-ref, BRAF-X1, and BRAF-X2. Figure S8. Real-time PCR detection of BRAF-ref, BRAF-X1 plus X2, BRAF-X1, and BRAF-X2. Figure S9. Box plot of the reads that span E17-E18.2, E18.2-E18b, E18.2-E19, and E17-E19. Figure S10. Stability of reference and X1/X2 BRAF mRNA. Figure S11. Correlation among the expression levels of the different BRAF isoforms in breast cancer, head and neck cancer, lung SCC, AML, and DLBCL. Figure S12. Lack of association of reference, X1, and X2 BRAF levels with age (left panels), gender (middle panels), and stage at diagnosis in primary and metastatic melanoma patients. Figure S13. Lack of association of age (left panels), gender (middle panels), and stage at diagnosis (right panels) with the ratio between BRAF-X1 and BRAF-ref levels (upper panels, red) and with the ratio between BRAF-X2 and BRAF-ref levels (lower panels, blue) in primary and metastatic melanoma patients. Figure S14. Tools for the detection of BRAF CDS plus the E19-derived 3’UTR. Figure S15. Length of BRAF-X1 and BRAF-X2 3’UTR in melanoma. Figure S16. siRNA-mediated downregulation of BRAF isoforms in melanoma cells. Figure S17. Cartoon summarizing the position of the primers and the siRNAs used to determine the levels and the identity of the Δ[3–10] splicing variant of BRAF. Figure S18. Real-time PCR detection of BRAF-ref (grey) and BRAF-X1 plus X2 (black) in vemurafenib-resistant clones and clonal populations. Figure S19. The 3’UTR of X1 and X [file 12943_2017_645_MOESM1_ESM.pdf]

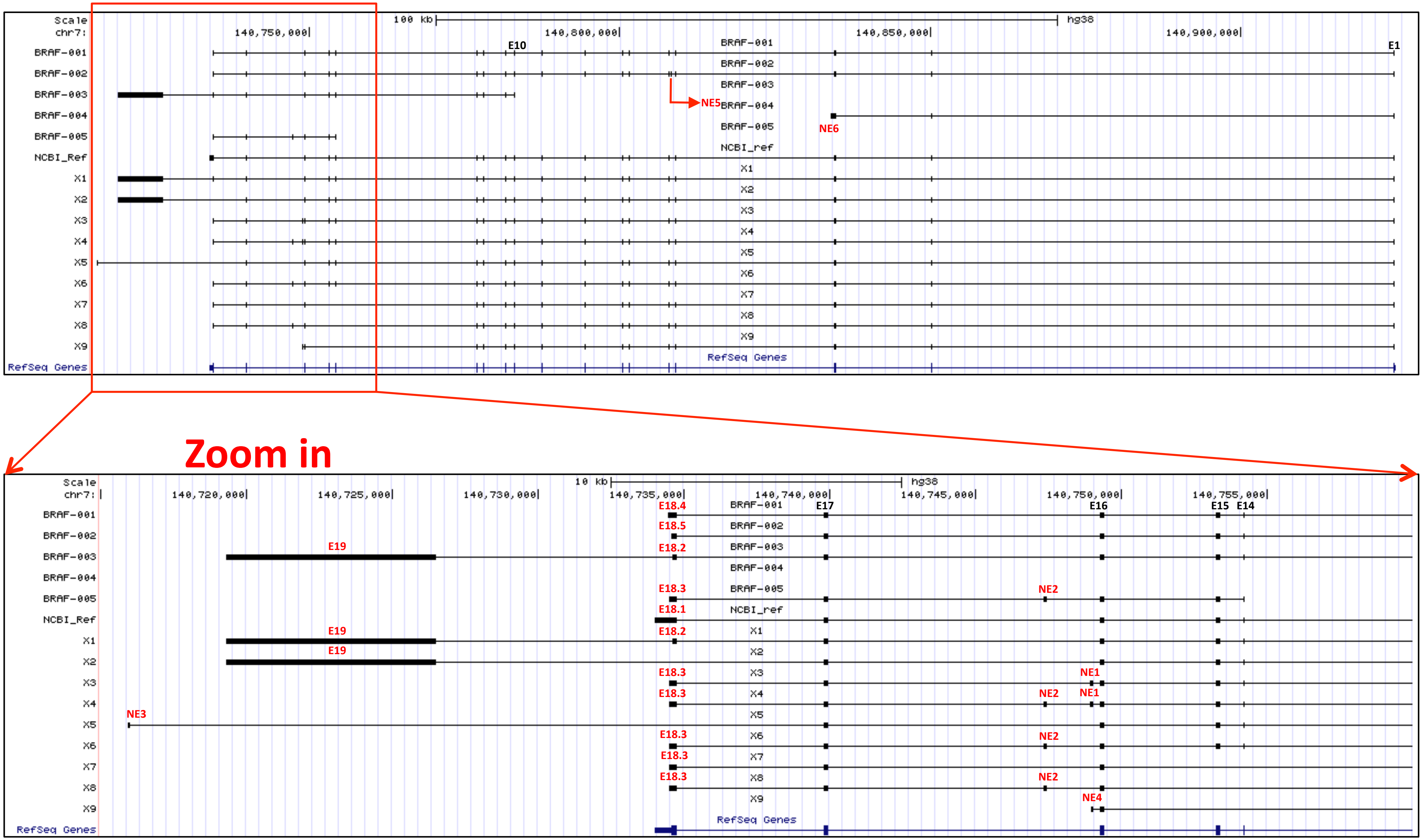

Supplementary Figure S1. Schematic representation of the exons belonging to the different transcript variants of human *BRAF* reported in NCBI and Ensembl.
(*top*) Human *BRAF* is located on chromosome 7q34 and is transcribed in the antisense orientation. Ensembl Genome Browser (http://www.ensembl.org/index.html) reports 5 *BRAF* transcript variants that are named *BRAF*-001, -002, -003, -004, and -005, with *BRAF*-001 being the reference. Analogously, NCBI (http://www.ncbi.nlm.nih.gov/nucleotide/) reports 10 *BRAF* transcripts, the reference (NM_004333.4) and X1 to X9 variants.
(*bottom*) Enlargement of the 3'end of *BRAF* gene (exon 14-19). Exons NE1-6 and E19 are not present in the reference sequence. E18.1-5 are 5 variants of exon 18 that differ in their length (see Supplementary Table S1).

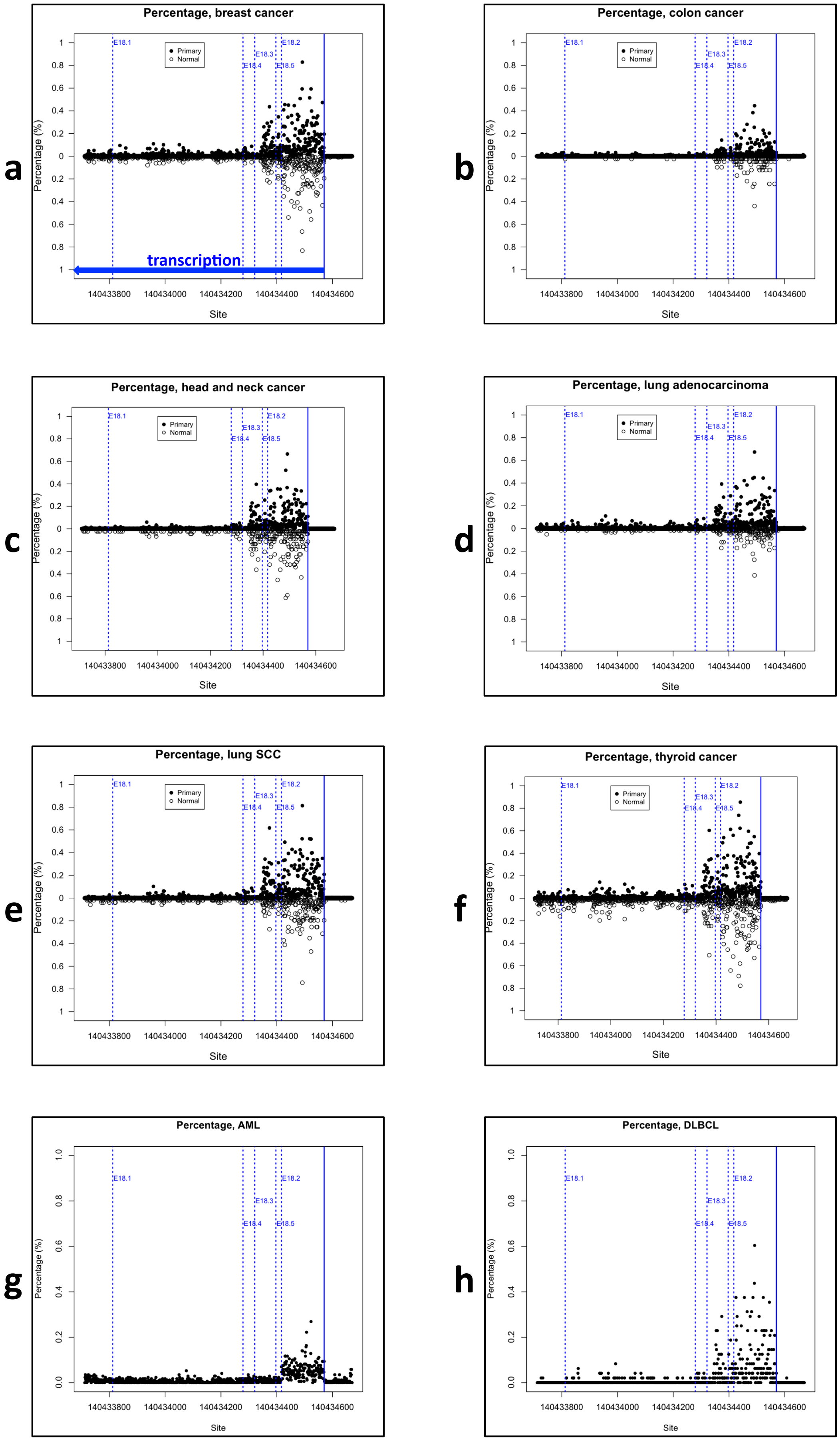

Supplementary Figure S2. Scan of exon 18 of *BRAF* in 8 cancer types.
Analysis of the length of *BRAF* 3’UTR by counting the reads that map to E18.1,2,3,4, and 5.

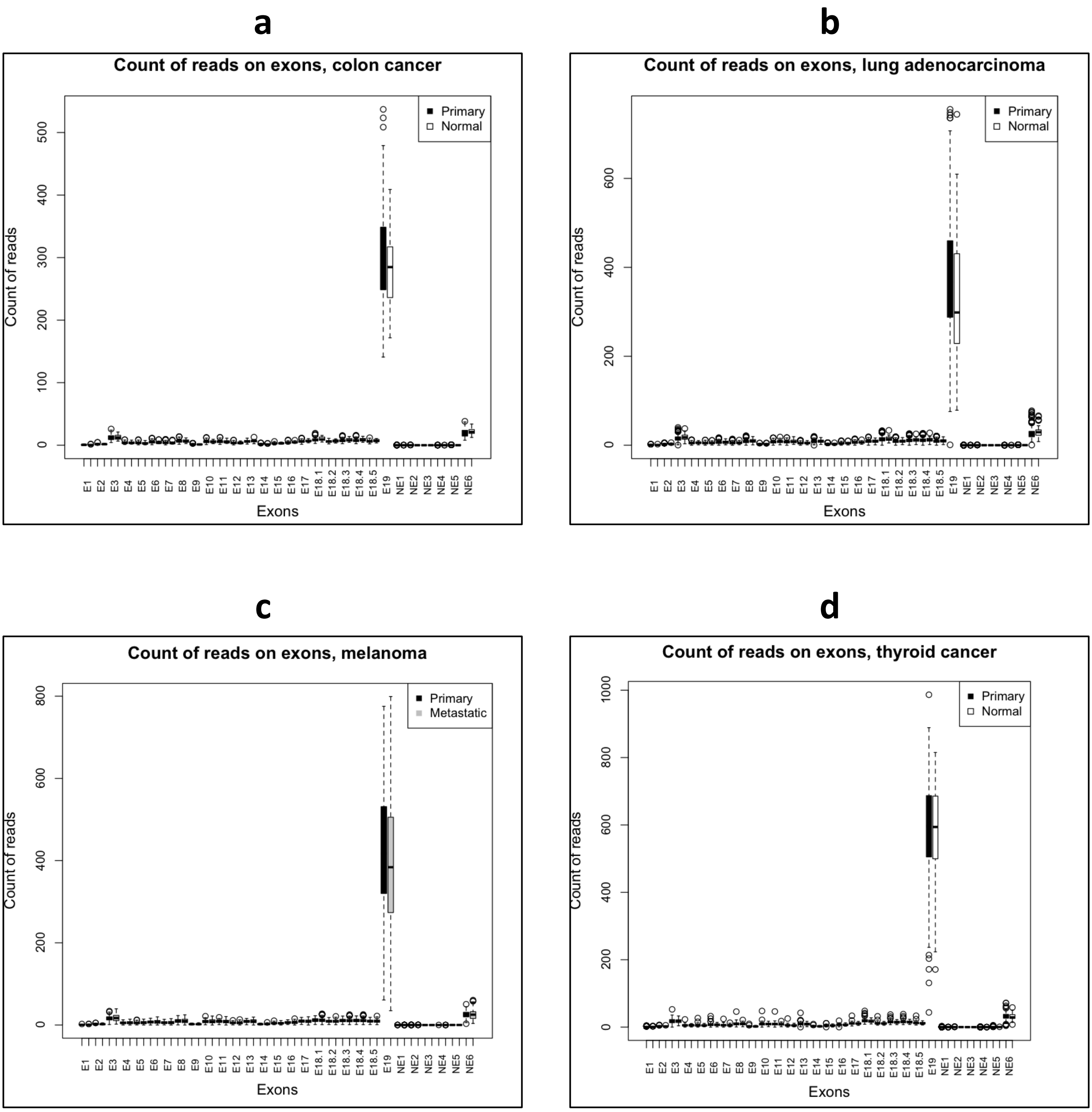

Supplementary Figure S3. Count of the reads mapping to all *BRAF* exons, E19 included.
(a) Colon cancer. **(b)** Lung adenocarcinoma. **(c)** Melanoma. **(d)** Thyroid cancer.

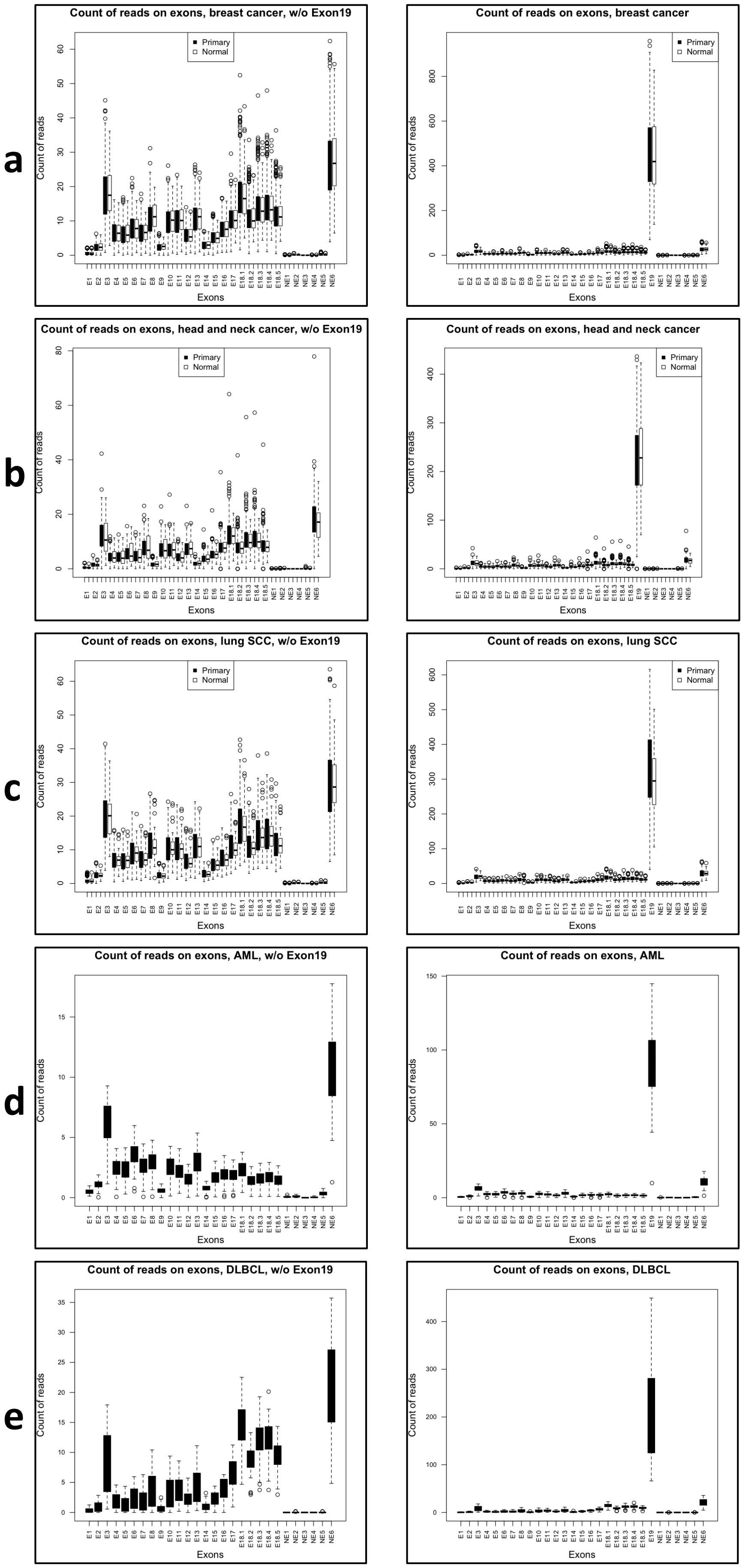

Supplementary Figure S4. Count of reads mapping to all *BRAF* exons (w/o E19 on the left and w E19 on the right).

(a) Breast cancer. **(b)** Head and neck cancer. **(c)** Lung SCC. **(d)** AML. **(e)** DLBCL.

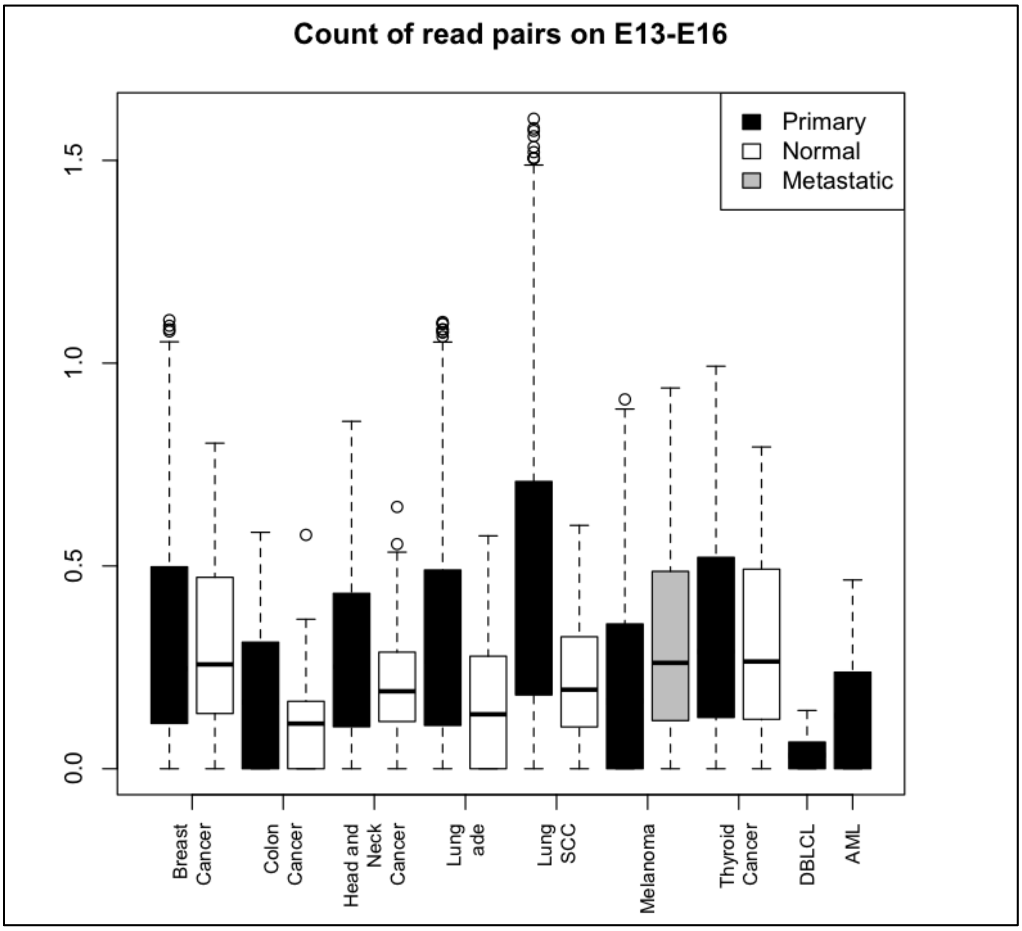

Supplementary Figure S5. Count of E13-E16 exon-spanning reads.

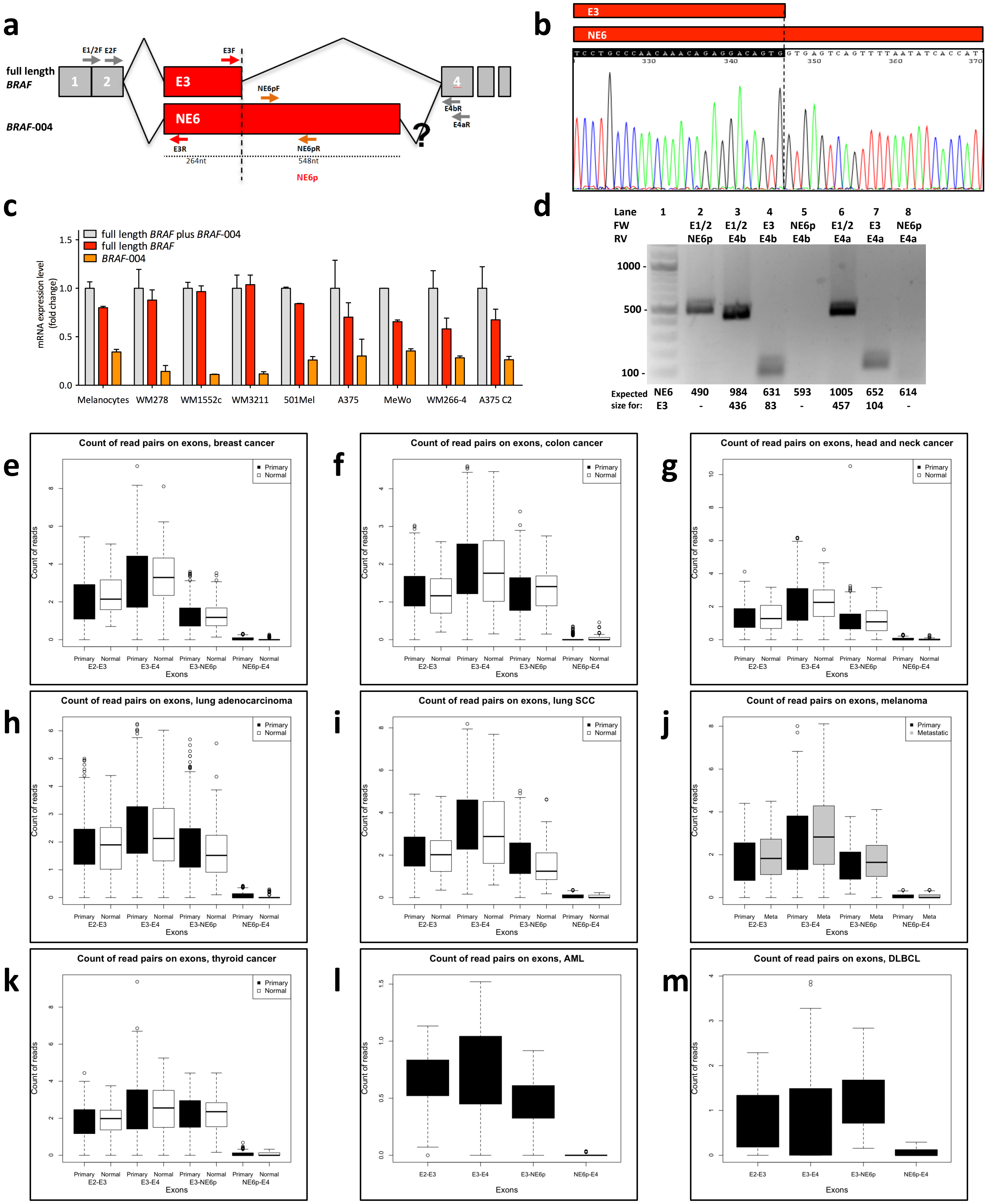

Supplementary Figure S6. *BRAF*-004 transcript variant is expressed but it is truncated.
(a) Schematic representation of E3 and NE6. The NE6-specific region is called NE6p. The primers used for PCR and/or qRT-PCR experiments are indicated as arrows. **(b)** Sanger sequencing of the PCR product obtained using *BRAF*-E1/2 F primer and *BRAF*-NE6p qRT-PCR R primer on the cDNA of A375 melanoma cells. The sequencing confirms that NE6 exon is transcribed. **(c)** Real-time PCR performed on the cDNA obtained from the indicated melanoma cell lines and the vemurafenib-resistant A375 C2 clone (see Fig. 6). In grey, the expression level of full length *BRAF* transcripts plus *BRAF*-004; in red, the expression level of full length *BRAF* transcripts; in orange, the expression level of *BRAF*-004. The graph represents the mean±SEM of 3 independent experiments. **(d)** PCR analysis of the *BRAF*-004 transcript variant using the indicated primer pairs (refer to panel **a** for positions). The presence of the expected PCR band in lane 2 confirms that NE6 is transcribed. However, the absence of the longer PCR product in lane 3, 4, 6 and 7, as well as the absence of any PCR product in lane 5 and 8 indicate that, downstream of NE6, *BRAF*-004 is truncated. Lane 1: 100bp ladder. **(e-m)** Count of the reads that span E2-E3 (full length *BRAF* transcripts plus *BRAF*-004), E3-E4 (full length *BRAF* transcripts) and E3-NE6p (*BRAF*-004) in the indicated tumor types. The presence of E3-NE6p spanning reads confirms that NE6p is transcribed. The absence of reads spanning NE6p and E4 confirms that *BRAF*-004 is truncated downstream of the NE6 exon. The lower counts of E2-E3 exon-spanning reads compared to E3-E4 exon-spanning reads has to be considered a technical artifact due to the bias that RNA-seq data obtained from polyA libraries have against the 5'end exons (see main text).

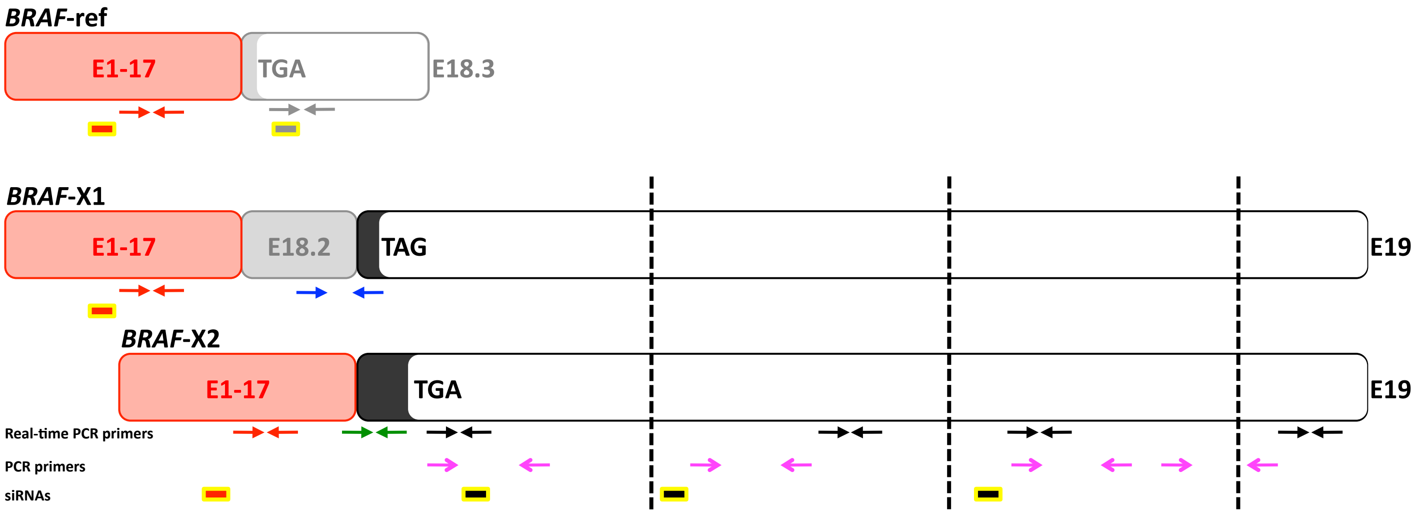

Supplementary Figure S7. Position of the primers/siRNAs used to detect/downregulate *BRAF*-ref, *BRAF*-X1, and *BRAF*-X2.

Exon 17, 18 and 19 are represented in red, grey and black, respectively. The 3'UTRs are in white.

ref*BRAF* qRT-PCR F/R, the primers used for real-time PCR amplification of *BRAF*-ref, are represented as grey arrows, while the siRNA used to knock-down this isoform (si-ref*BRAF*) is represented as a yellow and grey rectangle.

The primers used for real-time PCR amplification of *BRAF*-X1, *BRAF*-X2 and *BRAF*-X1 plus X2 are represented as blue, green and black arrows, respectively. They are called *BRAF*-X1 qRT-PCR F/R (1 pair), *BRAF*-X2 qRT-PCR F/R (1 pair) and *BRAF*-E19-1/2/3/4 qRT-PCR F/R (4 pairs).

The 4 primer pairs used for PCR amplification of *BRAF*-X1 plus X2 (*BRAF*-E19-1/2/3/4 F/R) are represented as open pink arrows. *BRAF*-E19-1 qRT-PCR F and *BRAF*-E19-1 F have the same sequence.

The siRNAs used to knock-down *BRAF*-X1 plus X2 (si-*BRAF*-E19-1/2/3) are represented as yellow and black rectangles.

The primers used for real-time PCR amplification of all *BRAF* isoforms (tot*BRAF* qRT-PCR F/R) are represented as red arrows, while the siRNA used for their knock-down (si-tot*BRAF*) is represented as a yellow and red rectangle.

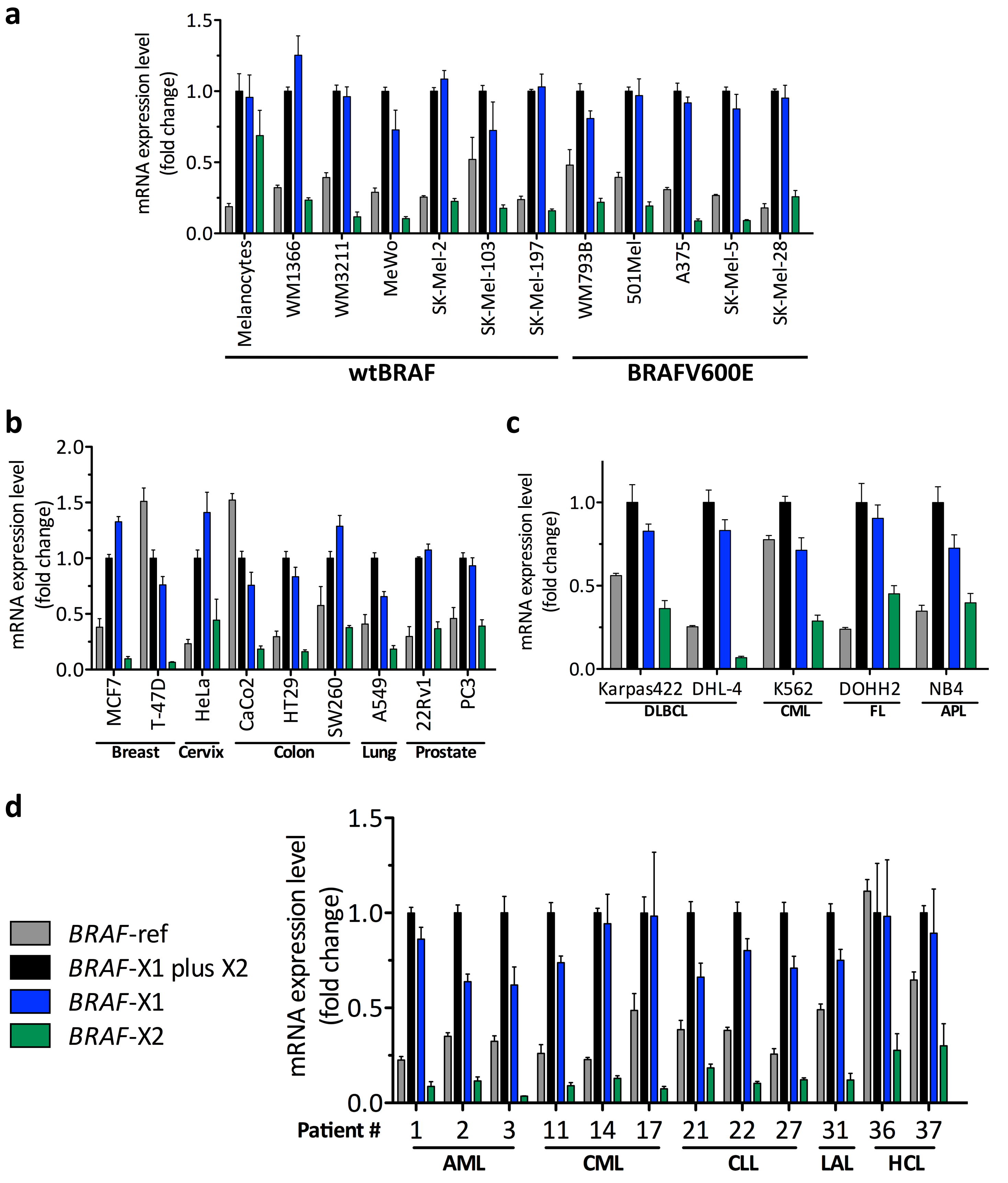

Supplementary Figure S8. Real-time PCR detection of *BRAF*-ref, *BRAF*-X1 plus X2, *BRAF*-X1, and *BRAF*-X2.
(a) Melanoma cell lines. **(b)** Cell lines derived from solid tumors. **(c)** Leukemia and lymphoma cell lines. **(d)** Leukemia and lymphoma patients. The expression level of *BRAF*-X1 plus X2 is taken as 1. *BRAF*-ref: grey; *BRAF*-X1 plus X2: black; *BRAF*-X1: blue; *BRAF*-X2: green. The graphs represent the mean±SEM of 3 independent experiments.

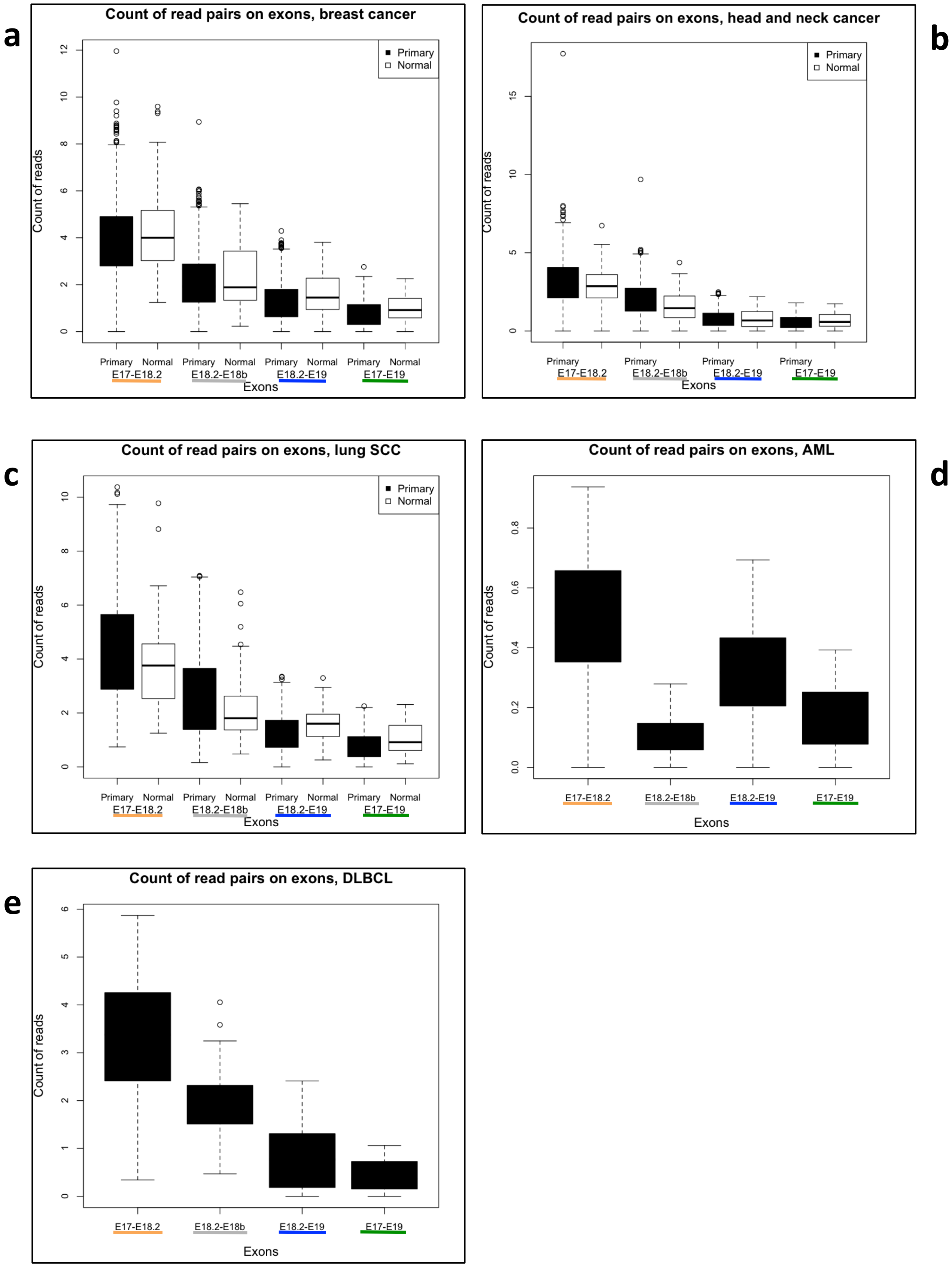

Supplementary Figure S9. Box plot of the reads that span E17-E18.2, E18.2-E18b, E18.2-E19, and E17-E19.
(a) Breast cancer. **(b)** Head and neck cancer. **(c)** Lung SCC. **(d)** AML. **(e)** DLBCL. For the color code, please refer to Figure 1c.

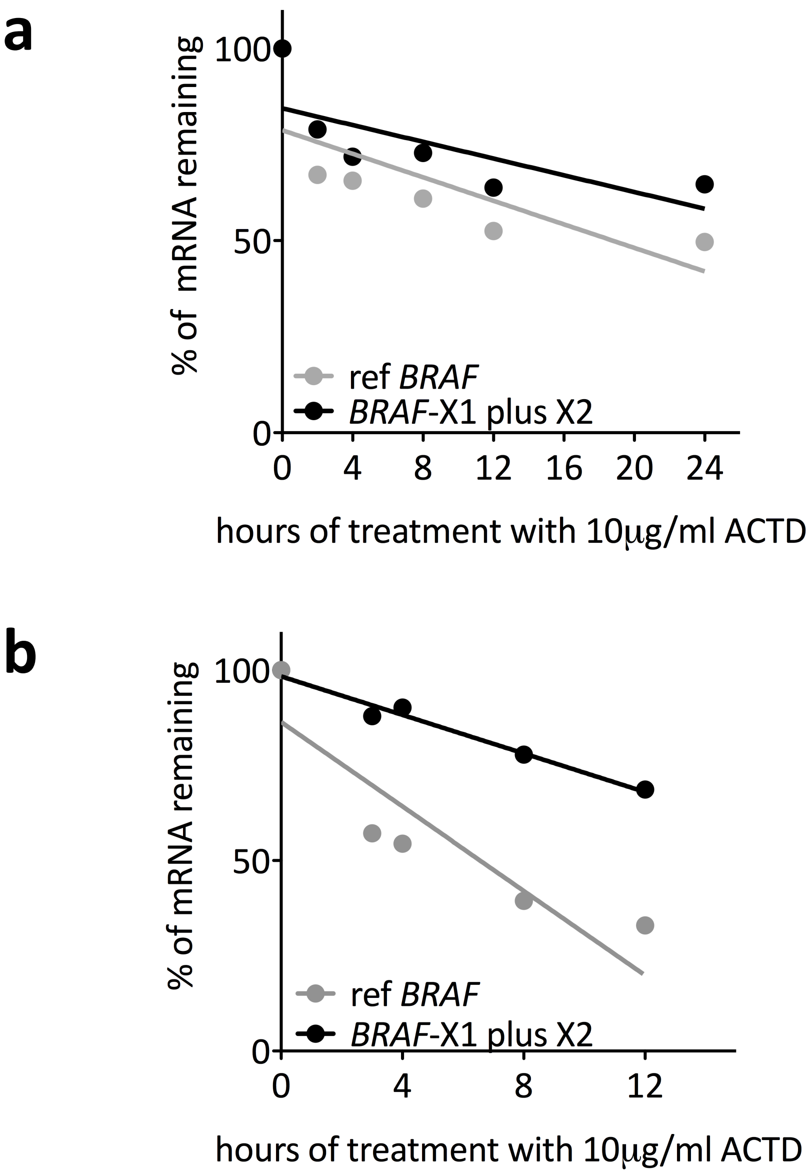

Supplementary Figure S10. Stability of reference and X1/X2 *BRAF* mRNA.
Real-time PCR quantification of *BRAF* isoforms at the indicated time points after treatment with 10ug/ml actinomycin D (ACTD). **(a)** A375 cells (homozygous BRAFV600E). **(b)** MeWo cells (wt BRAF). The graphs represent the mean of 3 independent experiments.

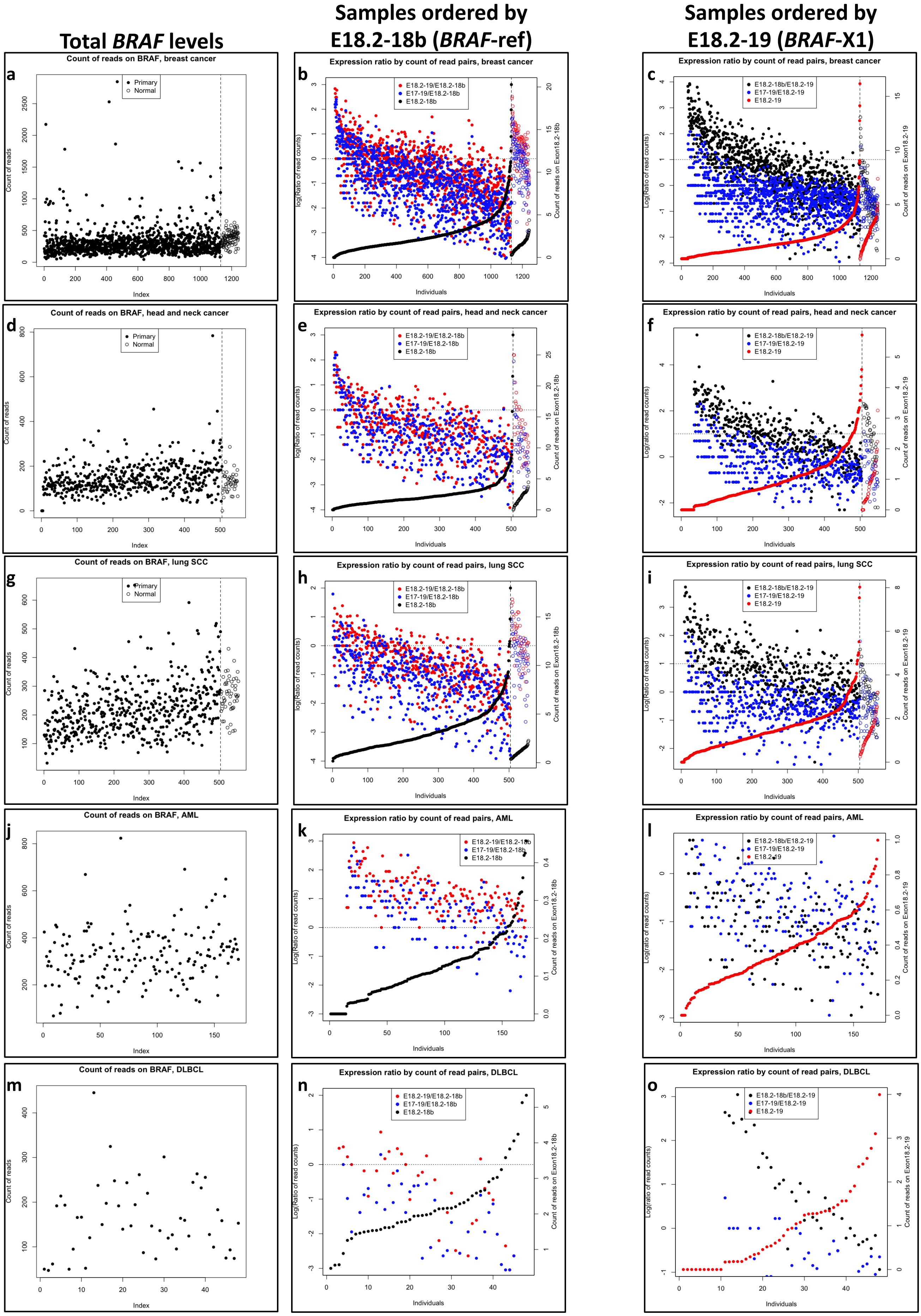

Supplementary Figure S11. Correlation among the expression levels of the different *BRAF* isoforms in breast cancer, head and neck cancer, lung SCC, AML, and DLBCL.
(a,d,g,j,m) Total number of *BRAF* reads across patients. **(b,e,h,k,n)** Expression ratios over the ref spanning reads. Samples were sorted by reads spanning E18.2-E18b (*BRAF*-ref, in black). Red dots are E18.2-E19/E18.2-E18b ratios (which means the X1/ref ratio) and blue dots are E17-E19/E18.2-E18b ratios (which means the X2/ref ratio). The data points are log transformed and the dotted line marks the 0, which means X1/ref ratio = 1 and X2/ref ratio = 1. **(c,f,i,l,o)** Expression ratios over the X1 spanning reads. Samples were sorted by reads spanning E18.2-E19 (*BRAF*-X1, in red). Black dots are E18.2-E18b/E18.2-E19 ratios (which means the ref/X1 ratio) and blue dots are E17-E19/E18.2-E19 ratios (which means the X2/X1 ratio). The data points are log transformed and the dotted line marks the 0, which means ref/X1 ratio = 1 and X2/X1 ratio = 1.
In the left and middle panels the samples are presented in the same order.

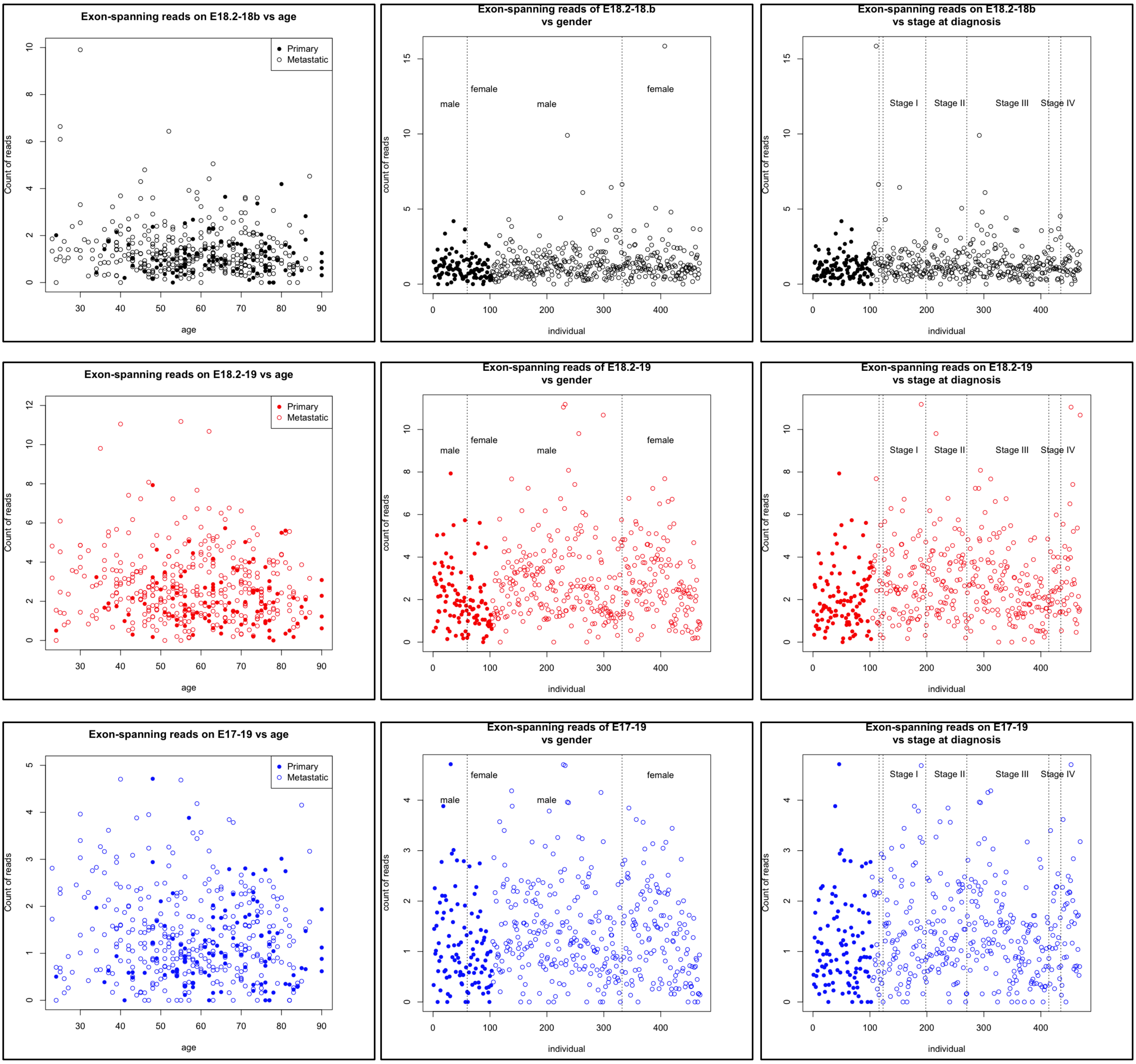

Supplementary Figure S12. Lack of association of reference, X1 and X2 *BRAF* levels with age (left panels), gender (middle panels), and stage at diagnosis in primary and metastatic melanoma patients.

The number of reads that span E18.2-18b (*BRAF*-ref, black), E18.2-19 (*BRAF*-X1, red), and E17-19 (*BRAF*-X2, blue) are reported.

Primary tumors: age range 24-90years (n = 103); n=43 females, n=60 males.

Metastatic tumors: age range 15-87years (n = 357); n=136 females, n=229 males; Stage I n=76, Stage II n=74, Stage III n=140, Stage IV n=20.

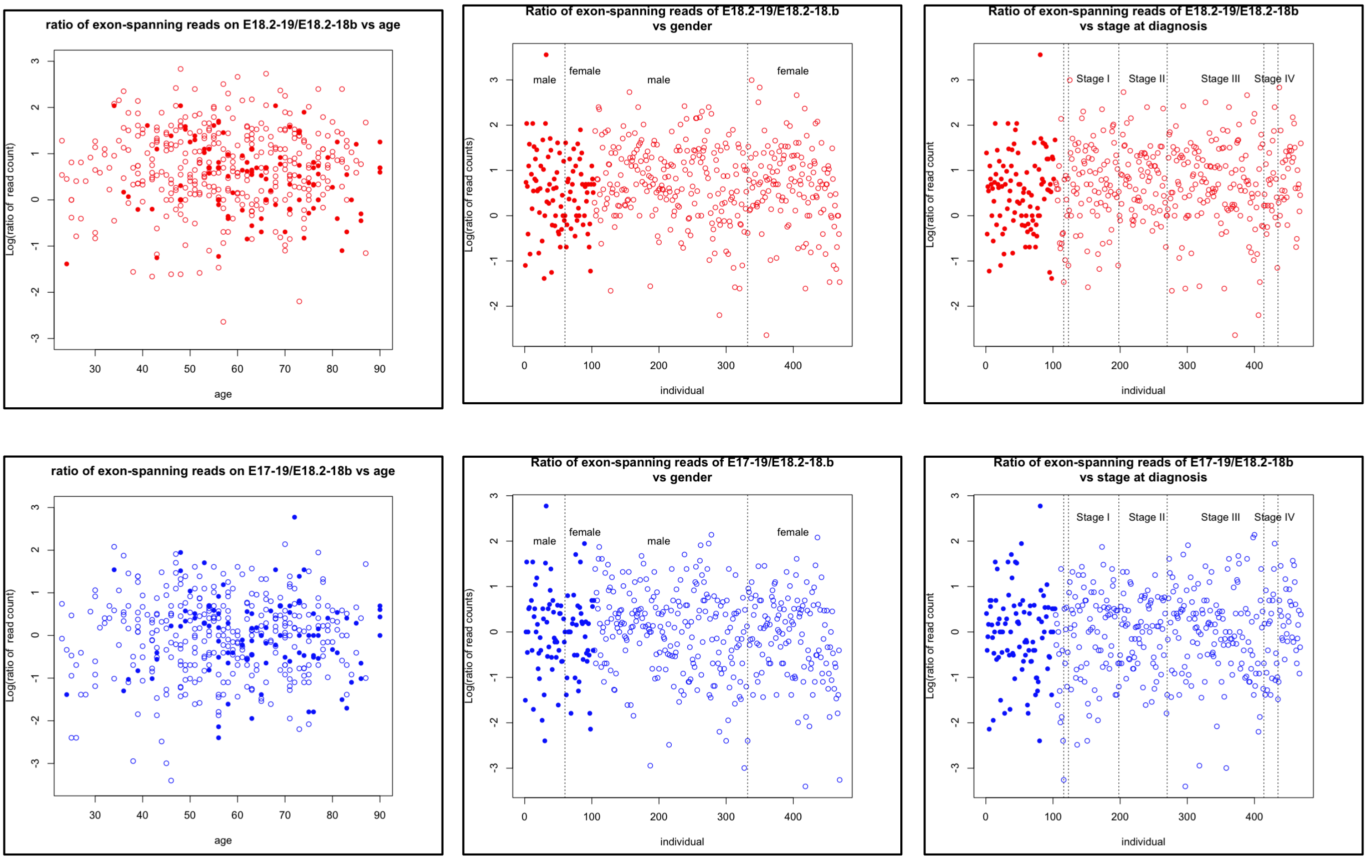

Supplementary Figure S13. Lack of association of age (left panels), gender (middle panels), and stage at diagnosis (right panels) with the ratio between *BRAF*-X1 and *BRAF*-ref levels (upper panels, red) and with the ratio between *BRAF*-X2 and *BRAF*-ref levels (lower panels, blue) in primary and metastatic melanoma patients.

Primary tumors: age range 24-90years (n = 103); n=43 females, n=60 males.

Metastatic tumors: age range 15-87years (n = 357); n=136 females, n=229 males; Stage I n=76, Stage II n=74, Stage III n=140, Stage IV n=20.

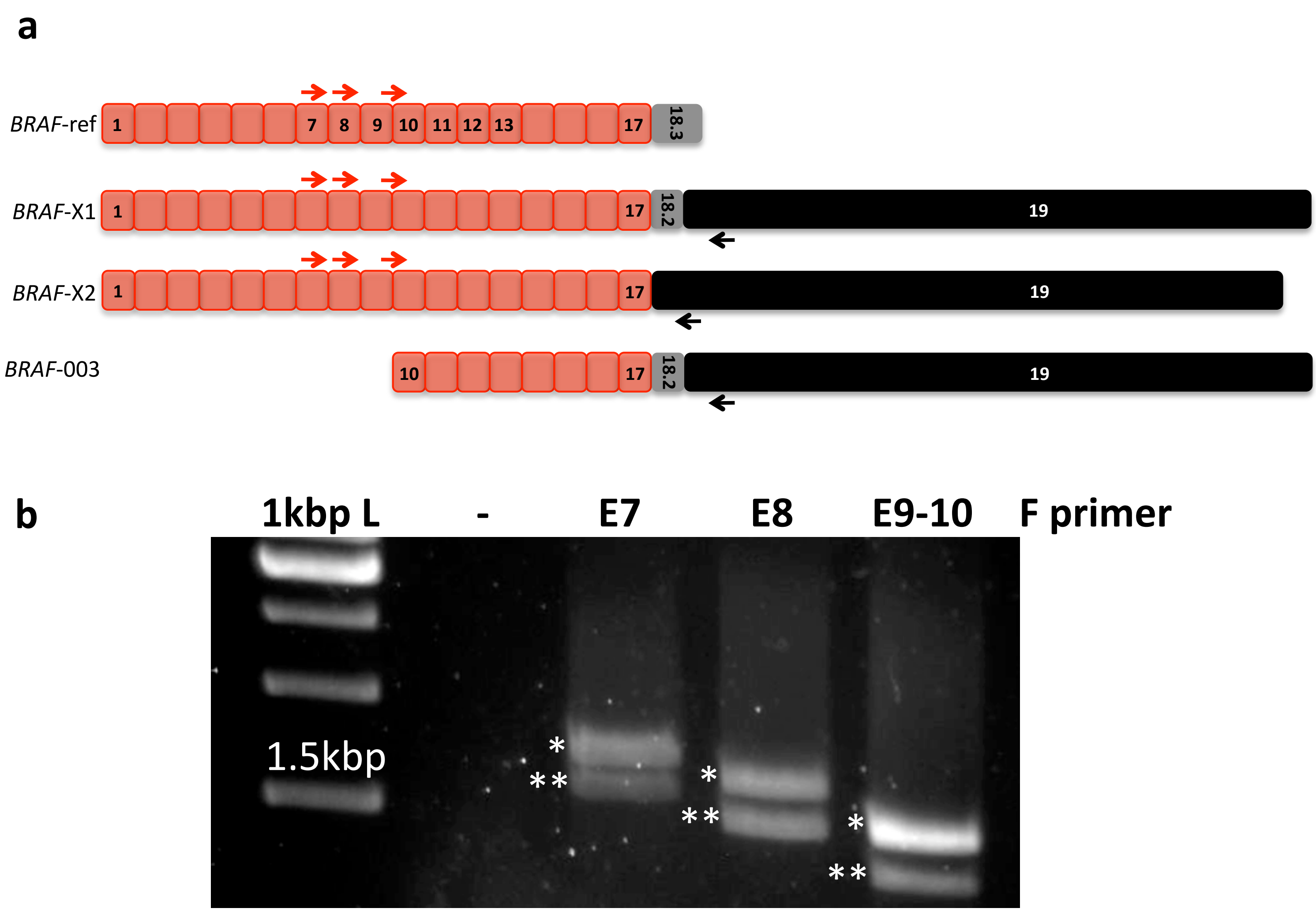

Expected size

	BRAF-X1*	*BRAF*-X2**
**E7 F**	1606	1452
**E8 F**	1505	1351
**E9-10**	1356	1202

Supplementary Figure S14. Tools for the detection of *BRAF* CDS plus the E19-derived 3'UTR.
(a) Primers location. The forward primers used for PCR amplification of *BRAF*-X1 and *BRAF*-X2 CDS (*BRAF*-E7 F, *BRAF*-E8 F, *BRAF*-E9-10 F) are represented as open red arrows. They were used together with *BRAF*-E19-1 qRT-PCR R (open black arrow). **(b)** Results of the PCR performed on A375 melanoma cells. The detection of a PCR doublet indicates that both *BRAF*-X1 and *BRAF*-X2 variants are expressed. In addition, it confirms that *BRAF* CDS and the E19-derived 3'UTR coexist in the same RNA molecule. In turn, this result rules out the possibility that the E19-derived 3'UTR is part only of *BRAF*-003 truncated transcript.

a

b

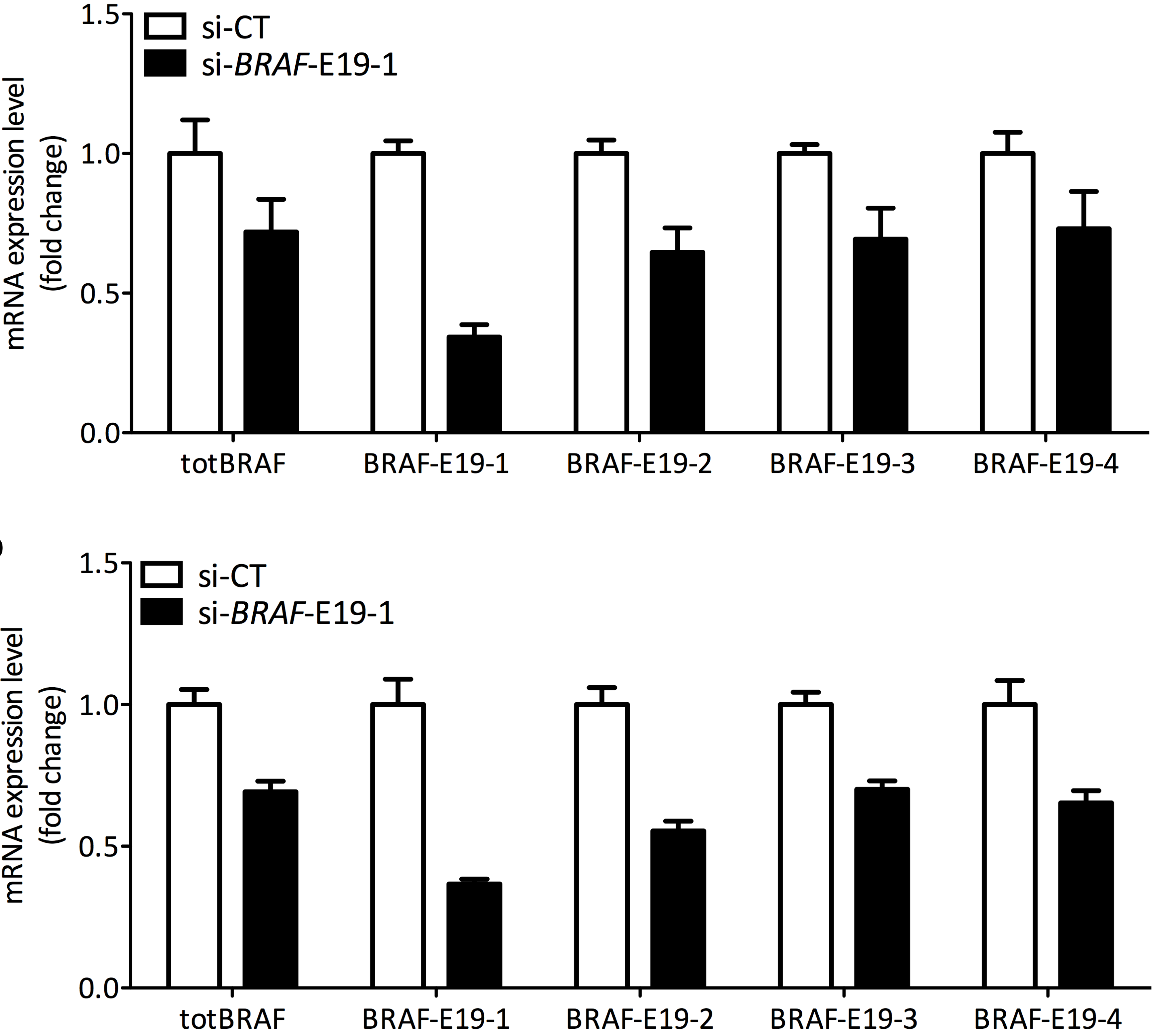

Supplementary Figure S15. Length of *BRAF*-X1 and *BRAF*-X2 3'UTR in melanoma.
Expression levels of *BRAF* CDS (detected using the tot*BRAF* qRT-PCR primers) and of different regions of the 3'UTR transcribed from E19 (detected using the *BRAF*-E19-1/2/3/4 qRT-PCR primer pairs) after the transfection of si-*BRAF*-E19-1 in 501Mel **(a)** and MeWo cells **(b)**. The graphs represent the mean±SD of 3 independent experiments.

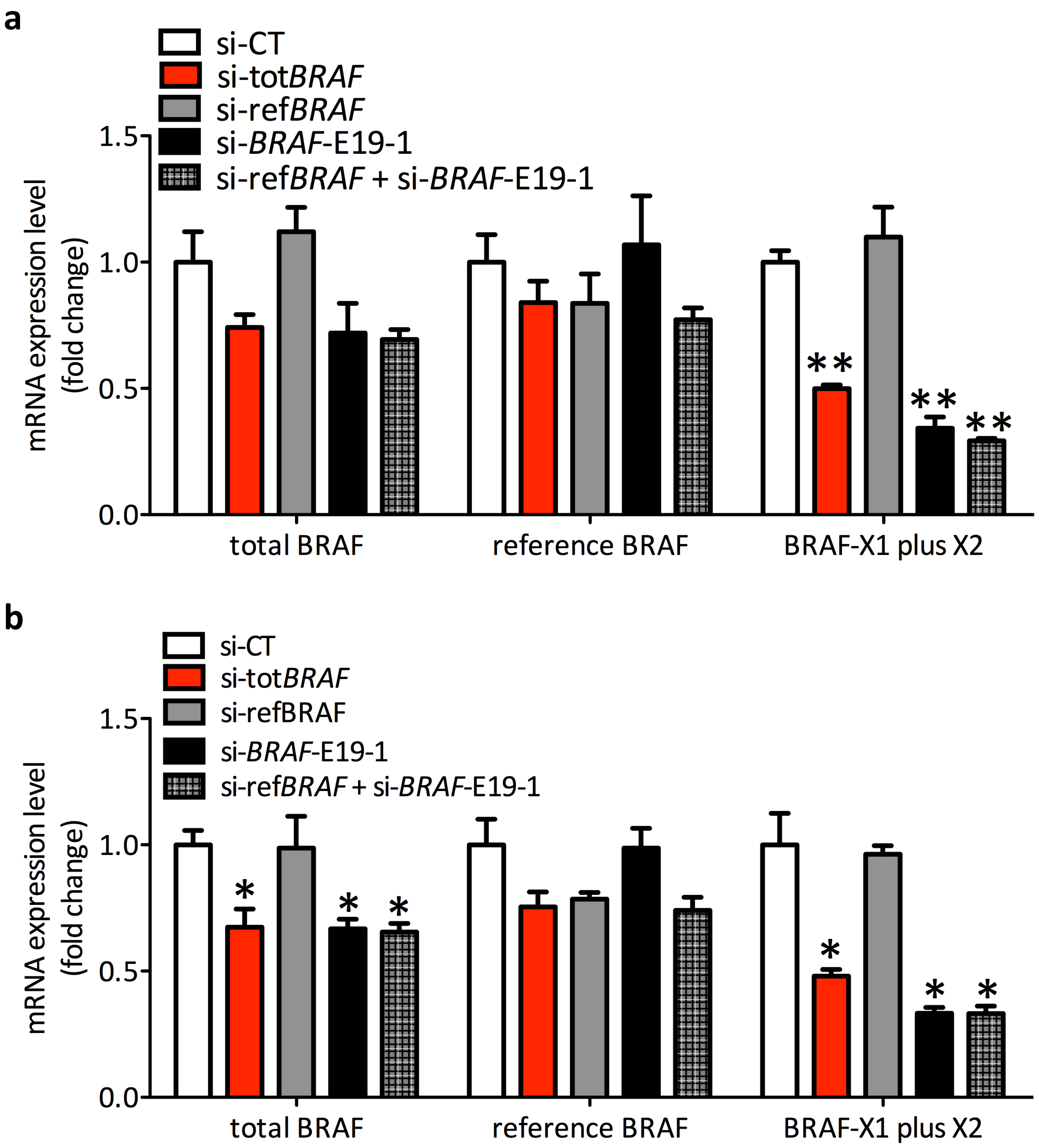

Supplementary Figure S16. siRNA-mediated downregulation of *BRAF* isoforms in melanoma cells.
Real-time PCR detection of total *BRAF*, *BRAF*-ref and *BRAF*-X1 plus X2 24h after the transfection of the indicated siRNAs in 501Mel **(a)** and in MeWo cells **(b)**. The graphs represent the mean±SD of 3 independent experiments.*p<0.05, **p<0.01.

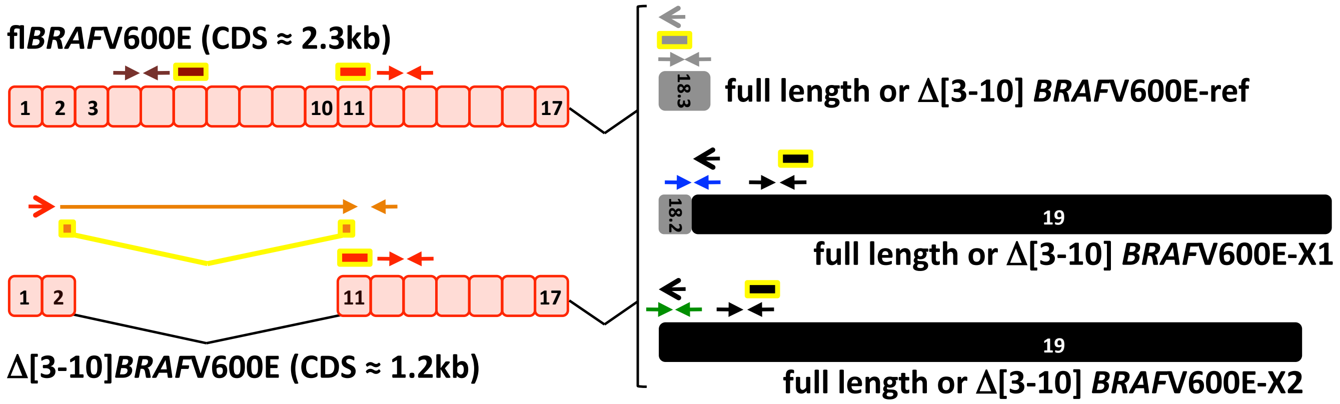

Supplementary Figure S17. Cartoon summarizing the position of the primers and the siRNAs used to determine the levels and the identity of the Δ[3-10] splicing variant of *BRAF*.

The PCR amplification of Δ[3-10]*BRAF* in its reference and X1/X2 isoforms was obtained using the *BRAF*-E1/2 F primer (red open arrow) and reverse primers that recognize the 3' end of *BRAF*-ref CDS (ref*BRAF*-STOP R) or *BRAF*-X1/X2 CDS (*BRAF*-X1-STOP R) (grey and black open arrow, respectively). The primers used for real-time PCR amplification of all *BRAF* isoforms (tot*BRAF* qRT-PCR F/R), *BRAF*-ref (ref*BRAF* qRT-PCR F/R), *BRAF*-X1 plus X2 (*BRAF*-E19-1 qRT-PCR F/R), *BRAF*-X1 (*BRAF*-X1 qRT-PCR F/R), *BRAF*-X2 (*BRAF*-X2 qRT-PCR F/R), full length *BRAF* (fl*BRAF* qRT-PCR F/R) and Δ[3-10]*BRAF* splicing variant (Δ[3-10]*BRAF* qRT-PCR F/R) are represented as red, grey, black, blue, green, brown and orange arrows, respectively. Analogously, the siRNAs used for the knock-down of all *BRAF* isoforms (si-tot*BRAF*), *BRAF*-ref (si-ref*BRAF*), *BRAF*-X1 plus X2 (si-*BRAF*-E19-1), full length *BRAF* (si-fl*BRAF*) and Δ[3-10]*BRAF* splicing variant (si-Δ[3-10]*BRAF*) are represented as yellow rectangles filled with red, grey, black, brown and orange, respectively.

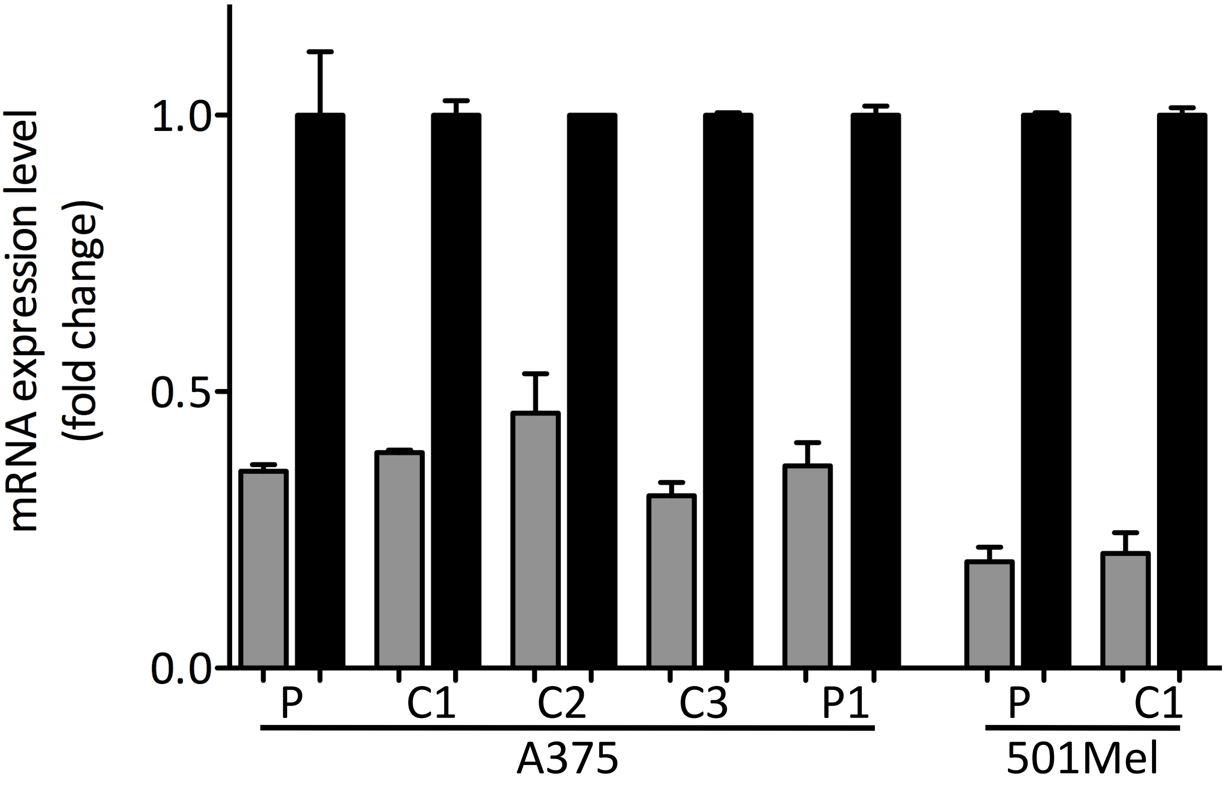

Supplementary Figure S18. Real-time PCR detection of *BRAF*-ref (grey) and *BRAF*-X1 plus X2 (black) in vemurafenib-resistant clones and clonal populations.

(*left*) P: parental A375 cells; A375 C1: vemurafenib-resistant clone carrying the Δ[3-10]BRAFV600E splicing variant; A375 C2: vemurafenib-resistant clone carrying the Δ[3-10]BRAFV600E splicing variant; A375 C3: vemurafenib-resistant clone carrying the Δ[3-8]BRAFV600E splicing variant; A375 P1: vemurafenib-resistant clonal population carrying the Δ[2-10]BRAFV600E splicing variant.

(*right*) P: parental 501Mel cells; 501Mel P1: vemurafenib-resistant clonal population carrying the Δ[3-10]BRAFV600E splicing variant.

The graphs represent the mean±SEM of 3 independent experiments.

a

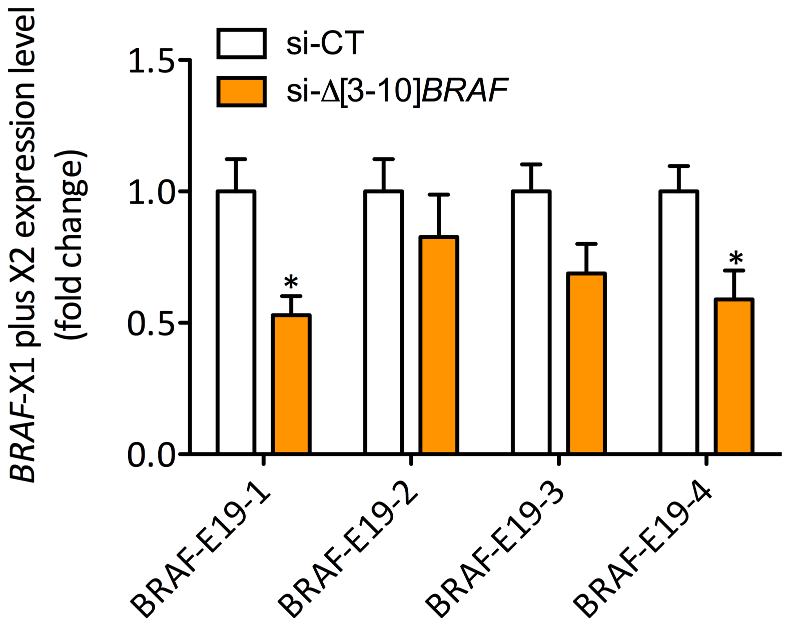

b

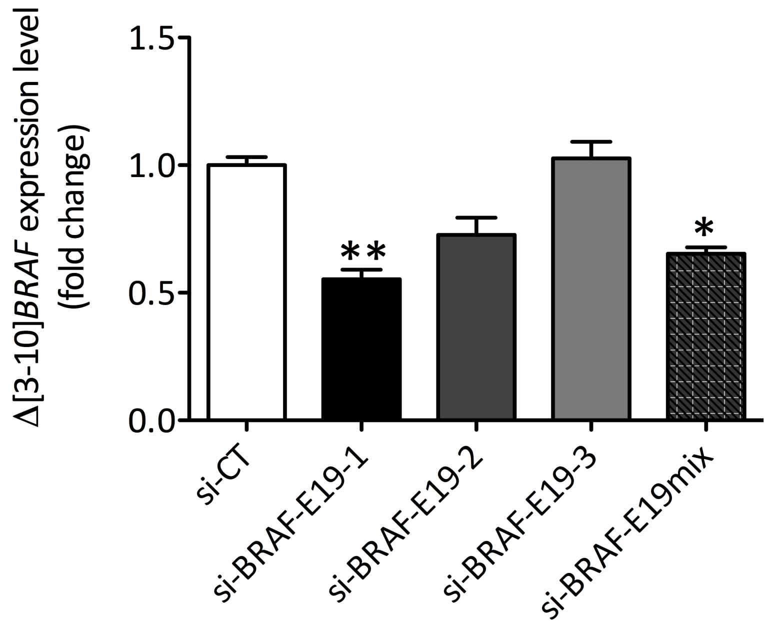

Supplementary Figure S19. The 3'UTR of X1 and X2 Δ[3-10]*BRAF* splicing variant is up to 7kb long.
(a) Real-time PCR detection of *BRAF*-X1 plus X2 24h after the transfection of si-Δ[3-10]*BRAF* in A375 C2 cells. Expression levels were detected using the 4 primer pairs that are located along the E19-derived 3'UTR.
(b) Real-time PCR detection of Δ[3-10]*BRAF* 24h after the transfection of si-*BRAF*-E19-1, si-*BRAF*-E19-2, si-*BRAF*-E19-3 or their mix in A375 C2 cells. The graphs represent the mean±SEM of 3 independent experiments. *p<0.05, **p<0.01.

a

Reference BRAF 766aa 84306MW
NP_004324.2 (http://www.ncbi.nlm.nih.gov/protein/NP_004324.2)

```
1   maalsggggg gaepgqalfn gdmepeagag agaaassaad paipeevwni kqmikltqeh
61  iealldkfgg ehnppsiyle ayeeytskld alqqreqqll eslgngtdfs vsssasmdtv
121 tsssssslsv lpsslsvfqn ptdvarsnpk spqkpivrvf lpnkqrtvvp arcgvtvrds
181 lkkalmmrgl ipeccavyri qdgekkpigw dtdiswltge elhvevlenv pltthnfvrk
241 tfftlafcdf crkllfqgfr cqtcgykfhq rcstevplmc vnydqldllf vskffehhpi
301 pqeeaslaet altsgsspsa pasdsigpqi ltspspsksi pipqpfrpad edhrnqfgqr
3
421 gpqrerksss ssedrnrmkt lgrrdssddw eipdgqitvg qrigsgsfgt vykgkwhgdv
481 avkmlnvtap tpqqlqafkn evgvlrktrh vnillfmgys tkpqlaivtq wcegsslyhh
541 lhiietkfem iklidiarqt aqgmdylhak siihrdlksn niflhedltv kigdfglatv
601 ksrwsgshqf eqlsgsilwm apevirmqdk npysfqsdvy afgivlyelm tgqlpysnin
661 nrdqiifmvg rgylspdlsk vrsncpkamk rlmaeclkkk rderplfpqi lasiellars
721 lpkihrsase pslnragfqt edfslyacas pktpiqaggy gafpvh
```

b

BRAF-X1 767aa 84448MW
XP_005250102.1 (http://www.ncbi.nlm.nih.gov/protein/XP_005250102.1)

```
1   maalsggggg gaepgqalfn gdmepeagag agaaassaad paipeevwni kqmikltqeh
61  iealldkfgg ehnppsiyle ayeeytskld alqqreqqll eslgngtdfs vsssasmdtv
121 tsssssslsv lpsslsvfqn ptdvarsnpk spqkpivrvf lpnkqrtvvp arcgvtvrds
181 lkkalmmrgl ipeccavyri qdgekkpigw dtdiswltge elhvevlenv pltthnfvrk
241 tfftlafcdf crkllfqgfr cqtcgykfhq rcstevplmc vnydqldllf vskffehhpi
301 pqeeaslaet altsgsspsa pasdsigpqi ltspspsksi pipqpfrpad edhrnqfgqr
361 drsssapnvh intiepvnid dlirdqgfrg dggsttglsa tppaslpgsl tnvkalqksp
421 gpqrerksss ssedrnrmkt lgrrdssddw eipdgqitvg qrigsgsfgt vykgkwhgdv
481 avkmlnvtap tpqqlqafkn evgvlrktrh vnillfmgys tkpqlaivtq wcegsslyhh
541 lhiietkfem iklidiarqt aqgmdylhak siihrdlksn niflhedltv kigdfglatv
601 ksrwsgshqf eqlsgsilwm apevirmqdk npysfqsdvy afgivlyelm tgqlpysnin
661 nrdqiifmvg rgylspdlsk vrsncpkamk rlmaeclkkk rderplfpqi lasiellars
721 lpkihrsase pslnragfqt edfslyacas pktpiqaggy gefaafk
```

c

BRAF-X2 758aa 83843MW
XP_005250103.1 (http://www.ncbi.nlm.nih.gov/protein/XP_005250103.1)

```
1   maalsggggg gaepgqalfn gdmepeagag agaaassaad paipeevwni kqmikltqeh
61  iealldkfgg ehnppsiyle ayeeytskld alqqreqqll eslgngtdfs vsssasmdtv
121 tsssssslsv lpsslsvfqn ptdvarsnpk spqkpivrvf lpnkqrtvvp arcgvtvrds
181 lkkalmmrgl ipeccavyri qdgekkpigw dtdiswltge elhvevlenv pltthnfvrk
241 tfftlafcdf crkllfqgfr cqtcgykfhq rcstevplmc vnydqldllf vskffehhpi
301 pqeeaslaet altsgsspsa pasdsigpqi ltspspsksi pipqpfrpad edhrnqfgqr
361 drsssapnvh intiepvnid dlirdqgfrg dggsttglsa tppaslpgsl tnvkalqksp
421 gpqrerksss ssedrnrmkt lgrrdssddw eipdgqitvg qrigsgsfgt vykgkwhgdv
481 avkmlnvtap tpqqlqafkn evgvlrktrh vnillfmgys tkpqlaivtq wcegsslyhh
541 lhiietkfem iklidiarqt aqgmdylhak siihrdlksn niflhedltv kigdfglatv
601 ksrwsgshqf eqlsgsilwm apevirmqdk npysfqsdvy afgivlyelm tgqlpysnin
661 nrdqiifmvg rgylspdlsk vrsncpkamk rlmaeclkkk rderplfpqe nlqpssshhh
721 gsicsyflsl vfvqfvnikt qfcssnlflk iqnfqcis
```

Supplementary Figure S20. Sequence of reference, X1, and X2 BRAF proteins.
The isoform-specific amino acids are highlighted in grey (reference, **a**), blue (X1, **b**), and green (X2, **c**).

Human BRAF-ref vs mouse Braf-ref

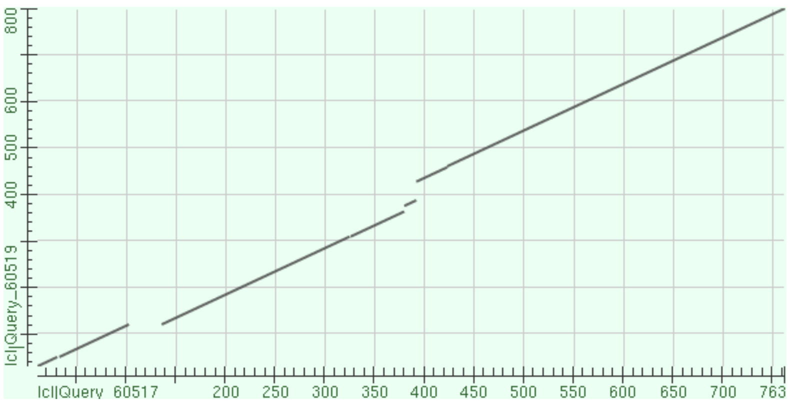

Identities	Positives	Gaps
695/806(86%)	703/806(87%)	90/806(11%)

```
1    MAALSGG-------------------GGGGAEPGQALFNGDMEPEAGAGAGAAASSAADPAIPEEVWNIKQMIKLTQEHI  61
1    MAALSGGGGSSSGGGGGGGGGGGGGDGGGGAEQGQALFNGDMEPEAGAGA--AASSAADPAIPEEVWNIKQMIKLTQEHI  78

62   EALLDKFGGEHNPPSIYLEAYEEYTSKLDALQQREQQLLESLGNGTDFSVSSSASMDTVTSSSSSSLSVLPSSLSVFQNP  141
79   EALLDKFGGEHNPPSIYLEAYEEYTSKLDALQQREQQLLESL---------------------------------VFQTP  125

142  TDVARSNPKSPQKPIVRVFLPNKQRTVVPARCGVTVRDSLKKALMMRGLIPECCAVYRIQDGEKKPIGWDTDISWLTGEE  221
126  TDASRNNPKSPQKPIVRVFLPNKQRTVVPARCGVTVRDSLKKALMMRGLIPECCAVYRIQDGEKKPIGWDTDISWLTGEE  205

222  LHVEVLENVPLTTHNFVRKTFFTLAFCDFCRKLLFQGFRCQTCGYKFHQRCSTEVPLMCVNYDQLDLLFVSKFFEHHPIP  301
206  LHVEVLENVPLTTHNFVRKTFFTLAFCDFCRKLLFQGFRCQTCGYKFHQRCSTEVPLMCVNYDQLDLLFVSKFFEHHPVP  285

302  QEEASLAETALTSGSSPSAPASDSIGPQILTSPSPSKSIPIPQPFRPADEDHRNQFGQRDRSSSAPNVHINTIEPVNID-  380
286  QEEASFPETALPSGSS-SAPPSDSTGPQILTSPSPSKSIPIPQPFRPADEDHRNQFGQRDRSSSAPNVHINTIEPVNIDE  364

381  -----------DLIRDQGFRGDG----------------------------------------GSTTGLSATPPASLPGS  409
365  KFPEVELQDQRDLIRDQGFRGDGAPLNQLMRCLRKYQSRTPSPLLHSVPSEIVFDFEPGPVFRGSTTGLSATPPASLPGS  444

410  LTNVKALQKSPGPQRERK--SSSSSEDRNRMKTLGRRDSSDDWEIPDGQITVGQRIGSGSFGTVYKGKWHGDVAVKMLNV  487
445  LTNVKALQKSPGPQRERKSSSSSSSEDRSRMKTLGRRDSSDDWEIPDGQITVGQRIGSGSFGTVYKGKWHGDVAVKMLNV  524

488  TAPTPQQLQAFKNEVGVLRKTRHVNILLFMGYSTKPQLAIVTQWCEGSSLYHHLHIIETKFEMIKLIDIARQTAQGMDYL  567
525  TAPTPQQLQAFKNEVGVLRKTRHVNILLFMGYSTKPQLAIVTQWCEGSSLYHHLHIIETKFEMIKLIDIARQTAQGMDYL  604

568  HAKSIIHRDLKSNNIFLHEDLTVKIGDFGLATVKSRWSGSHQFEQLSGSILWMAPEVIRMQDKNPYSFQSDVYAFGIVLY  647
605  HAKSIIHRDLKSNNIFLHEDLTVKIGDFGLATVKSRWSGSHQFEQLSGSILWMAPEVIRMQDKNPYSFQSDVYAFGIVLY  684

648  ELMTGQLPYSNINNRDQIIFMVGRGYLSPDLSKVRSNCPKAMKRLMAECLKKKRDERPLFPQILASIELLARSLPKIHRS  727
685  ELMTGQLPYSNINNRDQIIFMVGRGYLSPDLSKVRSNCPKAMKRLMAECLKKKRDERPLFPQILASIELLARSLPKIHRS  764

728  ASEPSLNRAGFQTEDFSLYACASPKTPIQAGGYGAFPVH-  766
765  ASEPSLNRAGFQTEDFSLYACASPKTPIQAGGYGEFAAFK  804
```

Human BRAF-X1 vs mouse Braf-ref

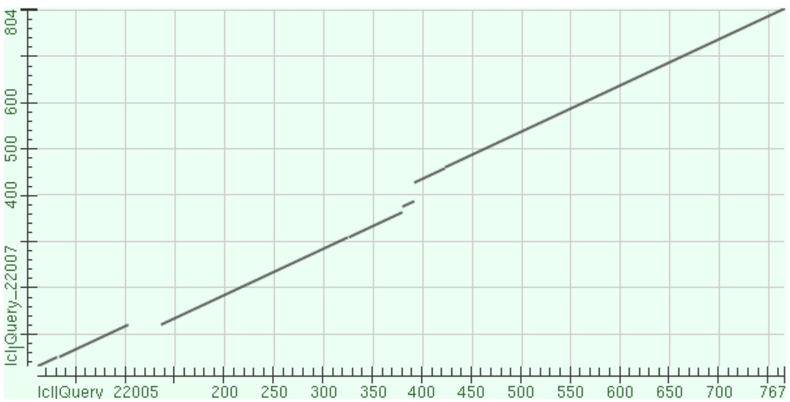

Identities	Positives	Gaps
700/810(86%)	708/810(87%)	90/810(11%)

```
1    MAALSGG-------------------GGGGAEPGQALFNGDMEPEAGAGAGAAASSAADPAIPEEVWNIKQMIKLTQEHI  61
1    MAALSGGGGSSSGGGGGGGGGGGGGDGGGGAEQGQALFNGDMEPEAGAGA--AASSAADPAIPEEVWNIKQMIKLTQEHI  78

62   EALLDKFGGEHNPPSIYLEAYEEYTSKLDALQQREQQLLESLGNGTDFSVSSSASMDTVTSSSSSSLSVLPSSLSVFQNP  141
79   EALLDKFGGEHNPPSIYLEAYEEYTSKLDALQQREQQLLESL---------------------------------VFQTP  125

142  TDVARSNPKSPQKPIVRVFLPNKQRTVVPARCGVTVRDSLKKALMMRGLIPECCAVYRIQDGEKKPIGWDTDISWLTGEE  221
126  TDASRNNPKSPQKPIVRVFLPNKQRTVVPARCGVTVRDSLKKALMMRGLIPECCAVYRIQDGEKKPIGWDTDISWLTGEE  205

222  LHVEVLENVPLTTHNFVRKTFFTLAFCDFCRKLLFQGFRCQTCGYKFHQRCSTEVPLMCVNYDQLDLLFVSKFFEHHPIP  301
206  LHVEVLENVPLTTHNFVRKTFFTLAFCDFCRKLLFQGFRCQTCGYKFHQRCSTEVPLMCVNYDQLDLLFVSKFFEHHPVP  285

302  QEEASLAETALTSGSSPSAPASDSIGPQILTSPSPSKSIPIPQPFRPADEDHRNQFGQRDRSSSAPNVHINTIEPVNID-  380
286  QEEASFPETALPSGSS-SAPPSDSTGPQILTSPSPSKSIPIPQPFRPADEDHRNQFGQRDRSSSAPNVHINTIEPVNIDE  364

381  -----------DLIRDQGFRGDG----------------------------------------GSTTGLSATPPASLPGS  409
365  KFPEVELQDQRDLIRDQGFRGDGAPLNQLMRCLRKYQSRTPSPLLHSVPSEIVFDFEPGPVFRGSTTGLSATPPASLPGS  444

410  LTNVKALQKSPGPQRERK--SSSSSEDRNRMKTLGRRDSSDDWEIPDGQITVGQRIGSGSFGTVYKGKWHGDVAVKMLNV  487
445  LTNVKALQKSPGPQRERKSSSSSSSEDRSRMKTLGRRDSSDDWEIPDGQITVGQRIGSGSFGTVYKGKWHGDVAVKMLNV  524

488  TAPTPQQLQAFKNEVGVLRKTRHVNILLFMGYSTKPQLAIVTQWCEGSSLYHHLHIIETKFEMIKLIDIARQTAQGMDYL  567
525  TAPTPQQLQAFKNEVGVLRKTRHVNILLFMGYSTKPQLAIVTQWCEGSSLYHHLHIIETKFEMIKLIDIARQTAQGMDYL  604

568  HAKSIIHRDLKSNNIFLHEDLTVKIGDFGLATVKSRWSGSHQFEQLSGSILWMAPEVIRMQDKNPYSFQSDVYAFGIVLY  647
605  HAKSIIHRDLKSNNIFLHEDLTVKIGDFGLATVKSRWSGSHQFEQLSGSILWMAPEVIRMQDKNPYSFQSDVYAFGIVLY  684

648  ELMTGQLPYSNINNRDQIIFMVGRGYLSPDLSKVRSNCPKAMKRLMAECLKKKRDERPLFPQILASIELLARSLPKIHRS  727
685  ELMTGQLPYSNINNRDQIIFMVGRGYLSPDLSKVRSNCPKAMKRLMAECLKKKRDERPLFPQILASIELLARSLPKIHRS  764

728  ASEPSLNRAGFQTEDFSLYACASPKTPIQAGGYGEFAAFK  767
765  ASEPSLNRAGFQTEDFSLYACASPKTPIQAGGYGEFAAFK  804
```

Supplementary Figure S21. Alignment between the sequence of human BRAF-ref (NP_004324.2, left) or BRAF-X1 (XP_005250102.1, right) and mouse Braf-ref (NP_647455.3).
The blue box highlights the identity between the C-terminal amino acids of human BRAF-X1 (and not of human BRAF-ref) and mouse Braf-ref.

Human BRAF-ref vs rat Braf-X1

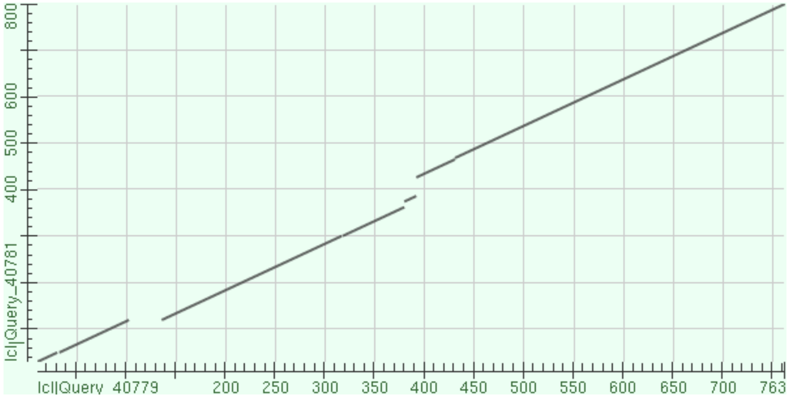

Identities 702/807(87%) Positives 707/807(87%) Gaps 91/807(11%)

```
1     MAALS------------------GGGGGGAEPGQALFNGDMEPEAGAGAGAAASSAADPAIPEEVWNIKQMIKLTQEHIE  62
1     MAALSGGGGSSSGGGGGGGGGGGGGGGGAEQGQALFNGDMEPEAGAGA--AASSAADPAIPEEVWNIKQMIKLTQEHIE  78

63    ALLDKFGGEHNPPSIYLEAYEEYTSKLDALQQREQQLLESLGNGTDFSVSSSASMDTVTSSSSSSLSVLPSSLSVFQNPT  142
79    ALLDKFGGEHNPPSIYLEAYEEYTSKLDALQQREQQLLESL----------------------------------VFQTPT  125

143   DVARSNPKSPQKPIVRVFLPNKQRTVVPARCGVTVRDSLKKALMMRGLIPECCAVYRIQDGEKKPIGWDTDISWLTGEEL  222
126   DVSRNNPKSPQKPIVRVFLPNKQRTVVPARCGVTVRDSLKKALMMRGLIPECCAVYRIQDGEKKPIGWDTDISWLTGEEL  205

223   HVEVLENVPLTTHNFVRKTFFTLAFCDFCRKLLFQGFRCQTCGYKFHQRCSTEVPLMCVNYDQLDLLFVSKFFEHHPIPQ  302
206   HVEVLENVPLTTHNFVRKTFFTLAFCDFCRKLLFQGFRCQTCGYKFHQRCSTEVPLMCVNYDQLDLLFVSKFFEHHPVPQ  285

303   EEASLAETALTSGSSPSAPASDSIGPQILTSPSPSKSIPIPQPFRPADEDHRNQFGQRDRSSSAPNVHINTIEPVNID--  380
286   EEAFSAETTLPSGCS-SAPPSDSIGPQILTSPSPSKSIPIPQPFRPADEDHRNQFGQRDRSSSAPNVHINTIEPVNIDEK  364

381   ----------DLIRDQGFRGDG----------------------------------------GSTTGLSATPPASLPGSL  410
365   FPEVELQDQRDLIRDQGFRGDGAPLNQLMRCLRKYQSRTPSPLLHSVPSEIVFDFEPGPVFRGSTTGLSATPPASLPGSL  444

411   TNVKALQKSPGPQRERKSSSSS---EDRNRMKTLGRRDSSDDWEIPDGQITVGQRIGSGSFGTVYKGKWHGDVAVKMLNV  487
445   TNVKALQKSPGPQRERKSSSSSSSTEDRSRMKTLGRRDSSDDWEIPDGQITVGQRIGSGSFGTVYKGKWHGDVAVKMLNV  524

488   TAPTPQQLQAFKNEVGVLRKTRHVNILLFMGYSTKPQLAIVTQWCEGSSLYHHLHIIETKFEMIKLIDIARQTAQGMDYL  567
525   TAPTPQQLQAFKNEVGVLRKTRHVNILLFMGYSTKPQLAIVTQWCEGSSLYHHLHIIETKFEMIKLIDIARQTAQGMDYL  604

568   HAKSIIHRDLKSNNIFLHEDLTVKIGDFGLATVKSRWSGSHQFEQLSGSILWMAPEVIRMQDKNPYSFQSDVYAFGIVLY  647
605   HAKSIIHRDLKSNNIFLHEDLTVKIGDFGLATVKSRWSGSHQFEQLSGSILWMAPEVIRMQDKNPYSFQSDVYAFGIVLY  684

648   ELMTGQLPYSNINNRDQIIFMVGRGYLSPDLSKVRSNCPKAMKRLMAECLKKKRDERPLFPQILASIELLARSLPKIHRS  727
685   ELMTGQLPYSNINNRDQIIFMVGRGYLSPDLSKVRSNCPKAMKRLMAECLKKKRDERPLFPQILASIELLARSLPKIHRS  764

728   ASEPSLNRAGFQTEDFSLYACASPKTPIQAGGYGAFPVH-  766
765   ASEPSLNRAGFQTEDFSLYACASPKTPIQAGGYGEFAAFK  804
```

Human BRAF-X1 vs rat Braf-X1

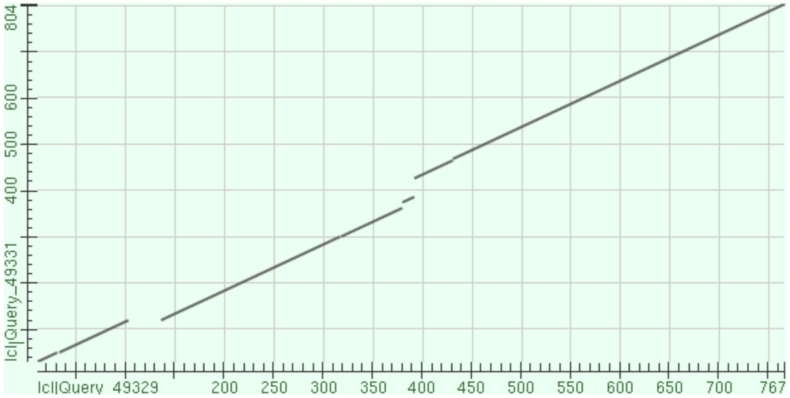

Identities 707/811(87%) Positives 712/811(87%) Gaps 91/811(11%)

```
1     MAALS------------------GGGGGGAEPGQALFNGDMEPEAGAGAGAAASSAADPAIPEEVWNIKQMIKLTQEHIE  62
1     MAALSGGGGSSSGGGGGGGGGGGGGGGGAEQGQALFNGDMEPEAGAGA--AASSAADPAIPEEVWNIKQMIKLTQEHIE  78

63    ALLDKFGGEHNPPSIYLEAYEEYTSKLDALQQREQQLLESLGNGTDFSVSSSASMDTVTSSSSSSLSVLPSSLSVFQNPT  142
79    ALLDKFGGEHNPPSIYLEAYEEYTSKLDALQQREQQLLESL----------------------------------VFQTPT  125

143   DVARSNPKSPQKPIVRVFLPNKQRTVVPARCGVTVRDSLKKALMMRGLIPECCAVYRIQDGEKKPIGWDTDISWLTGEEL  222
126   DVSRNNPKSPQKPIVRVFLPNKQRTVVPARCGVTVRDSLKKALMMRGLIPECCAVYRIQDGEKKPIGWDTDISWLTGEEL  205

223   HVEVLENVPLTTHNFVRKTFFTLAFCDFCRKLLFQGFRCQTCGYKFHQRCSTEVPLMCVNYDQLDLLFVSKFFEHHPIPQ  302
206   HVEVLENVPLTTHNFVRKTFFTLAFCDFCRKLLFQGFRCQTCGYKFHQRCSTEVPLMCVNYDQLDLLFVSKFFEHHPVPQ  285

303   EEASLAETALTSGSSPSAPASDSIGPQILTSPSPSKSIPIPQPFRPADEDHRNQFGQRDRSSSAPNVHINTIEPVNID--  380
286   EEAFSAETTLPSGCS-SAPPSDSIGPQILTSPSPSKSIPIPQPFRPADEDHRNQFGQRDRSSSAPNVHINTIEPVNIDEK  364

381   ----------DLIRDQGFRGDG----------------------------------------GSTTGLSATPPASLPGSL  410
365   FPEVELQDQRDLIRDQGFRGDGAPLNQLMRCLRKYQSRTPSPLLHSVPSEIVFDFEPGPVFRGSTTGLSATPPASLPGSL  444

411   TNVKALQKSPGPQRERKSSSSS---EDRNRMKTLGRRDSSDDWEIPDGQITVGQRIGSGSFGTVYKGKWHGDVAVKMLNV  487
445   TNVKALQKSPGPQRERKSSSSSSSTEDRSRMKTLGRRDSSDDWEIPDGQITVGQRIGSGSFGTVYKGKWHGDVAVKMLNV  524

488   TAPTPQQLQAFKNEVGVLRKTRHVNILLFMGYSTKPQLAIVTQWCEGSSLYHHLHIIETKFEMIKLIDIARQTAQGMDYL  567
525   TAPTPQQLQAFKNEVGVLRKTRHVNILLFMGYSTKPQLAIVTQWCEGSSLYHHLHIIETKFEMIKLIDIARQTAQGMDYL  604

568   HAKSIIHRDLKSNNIFLHEDLTVKIGDFGLATVKSRWSGSHQFEQLSGSILWMAPEVIRMQDKNPYSFQSDVYAFGIVLY  647
605   HAKSIIHRDLKSNNIFLHEDLTVKIGDFGLATVKSRWSGSHQFEQLSGSILWMAPEVIRMQDKNPYSFQSDVYAFGIVLY  684

648   ELMTGQLPYSNINNRDQIIFMVGRGYLSPDLSKVRSNCPKAMKRLMAECLKKKRDERPLFPQILASIELLARSLPKIHRS  727
685   ELMTGQLPYSNINNRDQIIFMVGRGYLSPDLSKVRSNCPKAMKRLMAECLKKKRDERPLFPQILASIELLARSLPKIHRS  764

728   ASEPSLNRAGFQTEDFSLYACASPKTPIQAGGYGEFAAFK  767
765   ASEPSLNRAGFQTEDFSLYACASPKTPIQAGGYGEFAAFK  804
```

Supplementary Figure S22. Alignment between the sequence of human BRAF-ref (NP_004324.2, left) or BRAF-X1 (XP_005250102.1, right) and rat Braf-X1 (XP_001070228.2).
The blue box highlights the identity between the C-terminal amino acids of human BRAF-X1 (and not of human BRAF-ref) and rat Braf-X1. In the rat there are 4 predicted Braf variants among which the X1 is the longest.

Humanreference BRAF vs pig Braf-X1

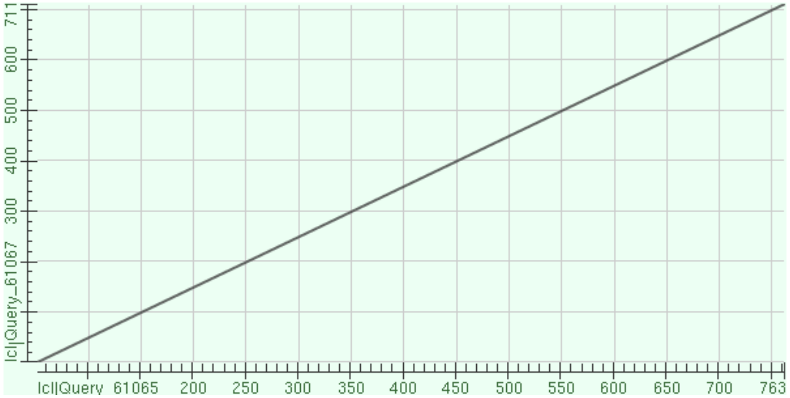

Identities	Positives	Gaps
704/711(99%)	706/711(99%)	0/711(0%)

```
1    MAALSGGGGGGAEPGQALFNGDMEPEAGAGAGAAASSAADPAIPEEVWNIKQMIKLTQEHIEALLDKFGGEHNPPSIYLE  80
1    ----------------------------------------------------MIKLTQEHIEALLDKFGGEHNPPSIYLE  28

81   AYEEYTSKLDALQQREQQLLESLGNGTDFSVSSSASMDTVTSSSSSSLSVLPSSLSVFQNPTDVARSNPKSPQKPIVRVF  160
29   AYEEYTSKLDALQQREQQLLESLGNGTDFSVSSSASTDTVTSSSSSSLSVLPSSLSVFQNPTDASRSNPKSPQKPIVRVF  108

161  LPNKQRTVVPARCGVTVRDSLKKALMMRGLIPECCAVYRIQDGEKKPIGWDTDISWLTGEELHVEVLENVPLTTHNFVRK  240
109  LPNKQRTVVPARCGVTVRDSLKKALMMRGLIPECCAVYRIQDGEKKPIGWDTDISWLTGEELHVEVLENVPLTTHNFVRK  188

241  TFFTLAFCDFCRKLLFQGFRCQTCGYKFHQRCSTEVPLMCVNYDQLDLLFVSKFFEHHPIPQEEASLAETALTSGSSPSA  320
189  TFFTLAFCDFCRKLLFQGFRCQTCGYKFHQRCSTEVPLMCVNYDQLDLLFVSKFFEHHPIPQEEASLAETALTSGSSPSA  268

321  PASDSIGPQILTSPSPSKSIPIPQPFRPADEDHRNQFGQRDRSSSAPNVHINTIEPVNIDDLIRDQGFRGDGGSTTGLSA  400
269  PPSDSLGPQILTSPSPSKSIPIPQPFRPADEDHRNQFGQRDRSSSAPNVHINTIEPVNIDDLIRDQGFRSDGGSTTGLSA  348

401  TPPASLPGSLTNVKALQKSPGPQRERKSSSSSEDRNRMKTLGRRDSSDDWEIPDGQITVGQRIGSGSFGTVYKGKWHGDV  480
349  TPPASLPGSLTNVKALQKSPGPQRERKSSSSSEDRNRMKTLGRRDSSDDWEIPDGQITVGQRIGSGSFGTVYKGKWHGDV  428

481  AVKMLNVTAPTPQQLQAFKNEVGVLRKTRHVNILLFMGYSTKPQLAIVTQWCEGSSLYHHLHIIETKFEMIKLIDIARQT  560
429  AVKMLNVTAPTPQQLQAFKNEVGVLRKTRHVNILLFMGYSTKPQLAIVTQWCEGSSLYHHLHIIETKFEMIKLIDIARQT  508

561  AQGMDYLHAKSIIHRDLKSNNIFLHEDLTVKIGDFGLATVKSRWSGSHQFEQLSGSILWMAPEVIRMQDKNPYSFQSDVY  640
509  AQGMDYLHAKSIIHRDLKSNNIFLHEDLTVKIGDFGLATVKSRWSGSHQFEQLSGSILWMAPEVIRMQDKNPYSFQSDVY  588

641  AFGIVLYELMTGQLPYSNINNRDQIIFMVGRGYLSPDLSKVRSNCPKAMKRLMAECLKKKRDERPLFPQILASIELLARS  720
589  AFGIVLYELMTGQLPYSNINNRDQIIFMVGRGYLSPDLSKVRSNCPKAMKRLMAECLKKKRDERPLFPQILASIELLARS  668

721  LPKIHRSASEPSLNRAGFQTEDFSLYACASPKTPIQAGGYGAFPVH-  766
669  LPKIHRSASEPSLNRAGFQTEDFSLYACASPKTPIQAGGYGEFAAFK  715
```

Human BRAF-X1 vs pig Braf-X1

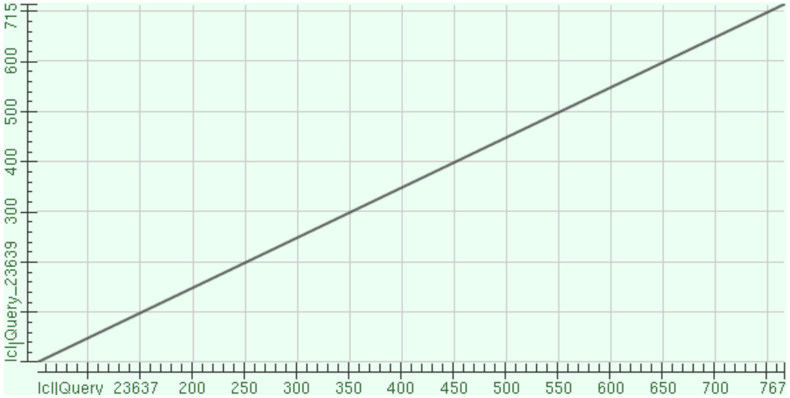

Identities	Positives	Gaps
709/715(99%)	711/715(99%)	0/715(0%)

```
1    MAALSGGGGGGAEPGQALFNGDMEPEAGAGAGAAASSAADPAIPEEVWNIKQMIKLTQEHIEALLDKFGGEHNPPSIYLE  80
1    ----------------------------------------------------MIKLTQEHIEALLDKFGGEHNPPSIYLE  28

81   AYEEYTSKLDALQQREQQLLESLGNGTDFSVSSSASMDTVTSSSSSSLSVLPSSLSVFQNPTDVARSNPKSPQKPIVRVF  160
29   AYEEYTSKLDALQQREQQLLESLGNGTDFSVSSSASTDTVTSSSSSSLSVLPSSLSVFQNPTDASRSNPKSPQKPIVRVF  108

161  LPNKQRTVVPARCGVTVRDSLKKALMMRGLIPECCAVYRIQDGEKKPIGWDTDISWLTGEELHVEVLENVPLTTHNFVRK  240
109  LPNKQRTVVPARCGVTVRDSLKKALMMRGLIPECCAVYRIQDGEKKPIGWDTDISWLTGEELHVEVLENVPLTTHNFVRK  188

241  TFFTLAFCDFCRKLLFQGFRCQTCGYKFHQRCSTEVPLMCVNYDQLDLLFVSKFFEHHPIPQEEASLAETALTSGSSPSA  320
189  TFFTLAFCDFCRKLLFQGFRCQTCGYKFHQRCSTEVPLMCVNYDQLDLLFVSKFFEHHPIPQEEASLAETALTSGSSPSA  268

321  PASDSIGPQILTSPSPSKSIPIPQPFRPADEDHRNQFGQRDRSSSAPNVHINTIEPVNIDDLIRDQGFRGDGGSTTGLSA  400
269  PPSDSLGPQILTSPSPSKSIPIPQPFRPADEDHRNQFGQRDRSSSAPNVHINTIEPVNIDDLIRDQGFRSDGGSTTGLSA  348

401  TPPASLPGSLTNVKALQKSPGPQRERKSSSSSEDRNRMKTLGRRDSSDDWEIPDGQITVGQRIGSGSFGTVYKGKWHGDV  480
349  TPPASLPGSLTNVKALQKSPGPQRERKSSSSSEDRNRMKTLGRRDSSDDWEIPDGQITVGQRIGSGSFGTVYKGKWHGDV  428

481  AVKMLNVTAPTPQQLQAFKNEVGVLRKTRHVNILLFMGYSTKPQLAIVTQWCEGSSLYHHLHIIETKFEMIKLIDIARQT  560
429  AVKMLNVTAPTPQQLQAFKNEVGVLRKTRHVNILLFMGYSTKPQLAIVTQWCEGSSLYHHLHIIETKFEMIKLIDIARQT  508

561  AQGMDYLHAKSIIHRDLKSNNIFLHEDLTVKIGDFGLATVKSRWSGSHQFEQLSGSILWMAPEVIRMQDKNPYSFQSDVY  640
509  AQGMDYLHAKSIIHRDLKSNNIFLHEDLTVKIGDFGLATVKSRWSGSHQFEQLSGSILWMAPEVIRMQDKNPYSFQSDVY  588

641  AFGIVLYELMTGQLPYSNINNRDQIIFMVGRGYLSPDLSKVRSNCPKAMKRLMAECLKKKRDERPLFPQILASIELLARS  720
589  AFGIVLYELMTGQLPYSNINNRDQIIFMVGRGYLSPDLSKVRSNCPKAMKRLMAECLKKKRDERPLFPQILASIELLARS  668

721  LPKIHRSASEPSLNRAGFQTEDFSLYACASPKTPIQAGGYGEFAAFK  767
669  LPKIHRSASEPSLNRAGFQTEDFSLYACASPKTPIQAGGYGEFAAFK  715
```

Supplementary Figure S23. Alignment between the sequence of human BRAF-ref (NP_004324.2, left) or BRAF-X1 (XP_005250102.1, right) and pig Braf-X1 (XP_005654324.1).
The blue box highlights the identity between the C-terminal amino acids of human BRAF-X1 (and not of human BRAF-ref) and pig Braf-X1. In the pig there are 3 predicted Braf variants among which the X1 is the longest.

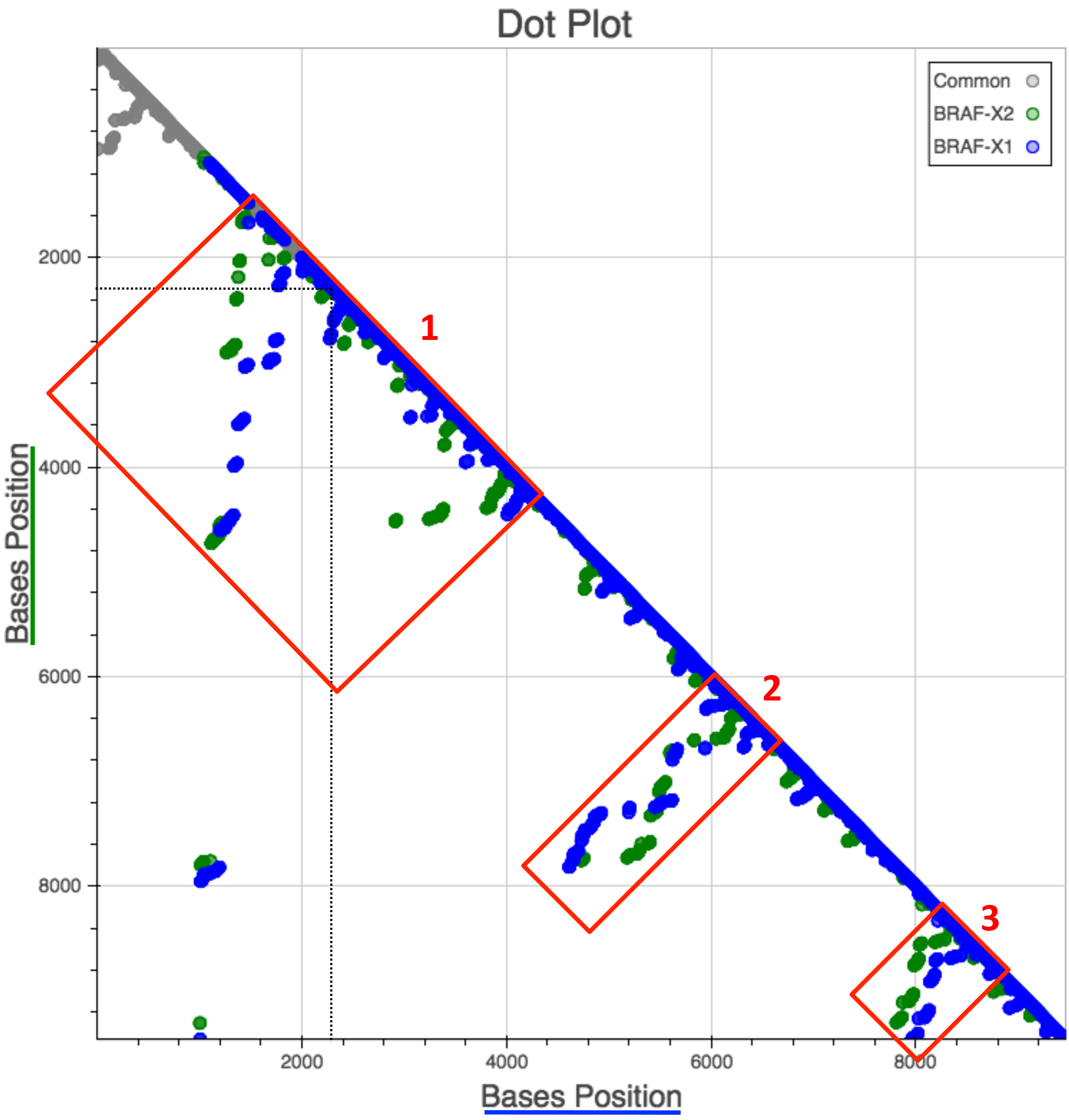

Supplementary Figure S24. Dot plot of the secondary structure of *BRAF*-X1 (blue) and *BRAF*-X2 (green) mRNA sequences. The most stable secondary structures of *BRAF*-X1 mRNA (9464nt, starting from the ATG) and *BRAF*-X2 mRNA (9310nt, starting from the ATG) were predicted using UNAfold program version 3.8 and were used to build the dot plot (see Methods for details). The dots located on the diagonal represent secondary structures that are shared by the 2 mRNA molecules (grey), while the green and blue dots represent isoform-specific secondary structures. The red boxes highlight the regions of highest dissimilarity. The dotted line marks the position of the STOP codon.

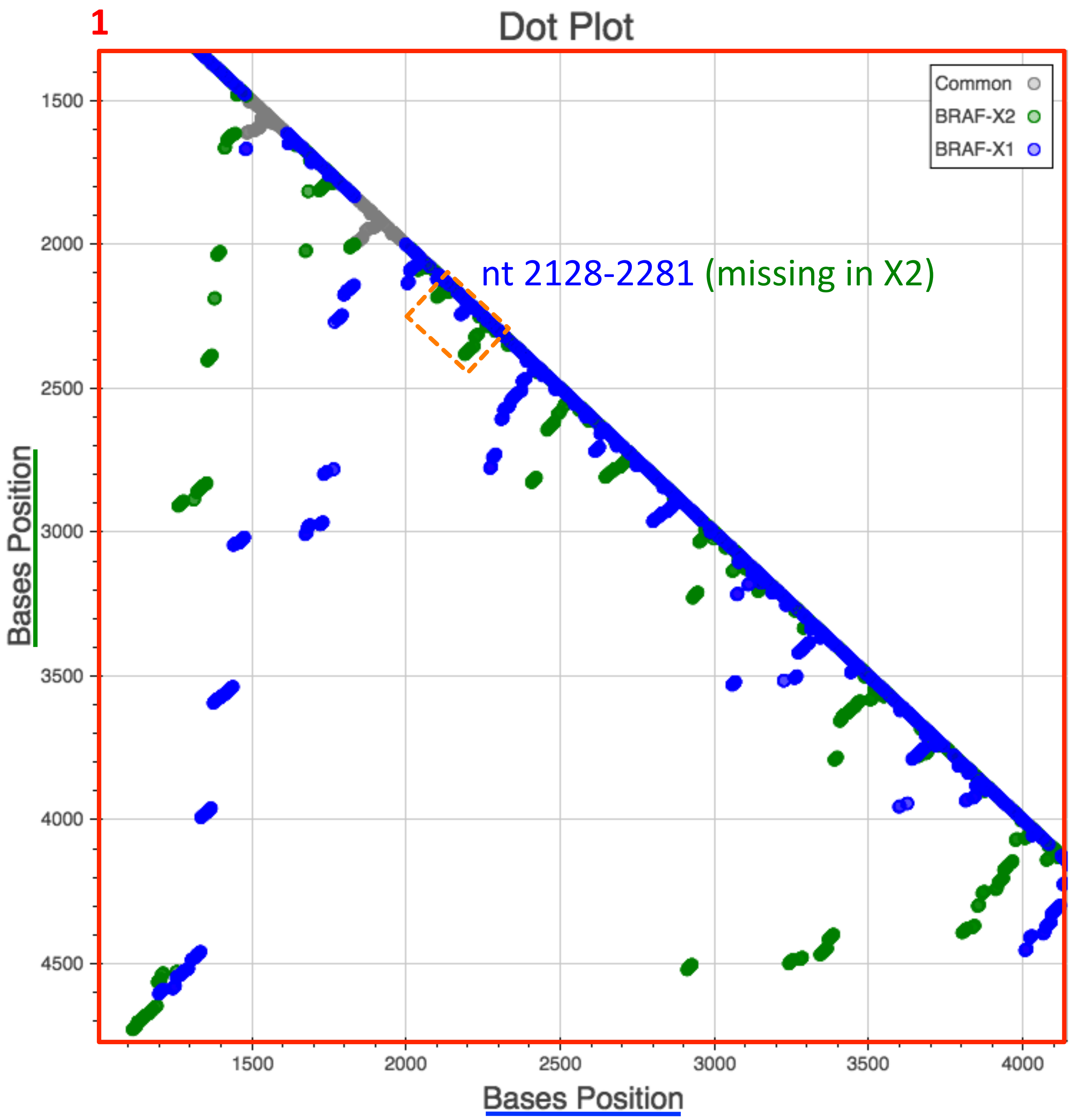

Supplementary Figure S25. Enlargement of box1 as reported in Supplementary Figure S24.
In spite of the fact that the region missing in the X2 isoform is only the one highlighted in orange (154nt), the direct splicing of exon 17 with exon 19 that occurs in the *BRAF*-X2 variant causes changes in the secondary structure of the mRNA molecule that extend way upstream in the coding sequence and downstream in the 3'UTR (the involved region encompasses approximately nt. 1500-4000).

a BRAF-ref

Serine/threonine-protein kinase B-raf OS=Homo sapiens GN=BRAF PE=1 SV=4 - [BRAF_HUMAN]

```
MAALSGGGGG GAEPGQALFN GDMEPEAGAG AGAAASSAAD PAIPEEVWNI KQMIKLTQEH IEALLDKFGG EHNPPSIYLE
AYEEYTSKLD ALQQREQQLL ESLGNGTDFS VSSSASMDTV TSSSSSSLSV LPSSLSVFQN PTDVARSNPK SPQKPIVRVF
LPNKQRTVVP ARCGVTVRDS LKKALMMRGL IPECCAVYRI QDGEKKPIGW DTDISWLTGE ELHVEVLENV PLTTHNFVRK
TFFTLAFCDF CRKLLFQGFR CQTCGYKFHQ RCSTEVPLMC VNYDQLDLLF VSKFFEHHPI PQEEASLAET ALTSGSSPSA
PASDSIGPQI LTSPSPSKSI PIPQPFRPAD EDHRNQFGQR DRSSSAPNVH INTIEPVNID DLIRDQGFRG DGGSTTGLSA
TPPASLPGSL TNVKALQKSP GPQRERKSSS SSEDRNRMKT LGRRDSSDDW EIPDGQITVG QRIGSGSFGT VYKGKWHGDV
AVKMLNVTAP TPQQLQAFKN EVGVLRKTRH VNILLFMGYS TKPQLAIVTQ WCEGSSLYHH LHIIETKFEM IKLIDIARQT
AQGMDYLHAK SIIHRDLKSN NIFLHEDLTV KIGDFGLATV KSRWSGSHQF EQLSGSILWM APEVIRMQDK NPYSFQSDVY
AFGIVLYELM TGQLPYSNIN NRDQIIFMVG RGYLSPDLSK VRSNCPKAMK RLMAECLKKK RDERPLFPQI LASIELLARS
LPKIHRSASE PSLNRAGFQT EDFSLYACAS PKTPIQAGGY GAFPVH
```

BRAF-X1

PREDICTED: serine/threonine-protein kinase B-raf isoform X1 [Homo sapiens]

```
MAALSGGGGG GAEPGQALFN GDMEPEAGAG AGAAASSAAD PAIPEEVWNI KQMIKLTQEH IEALLDKFGG EHNPPSIYLE
AYEEYTSKLD ALQQREQQLL ESLGNGTDFS VSSSASMDTV TSSSSSSLSV LPSSLSVFQN PTDVARSNPK SPQKPIVRVF
LPNKQRTVVP ARCGVTVRDS LKKALMMRGL IPECCAVYRI QDGEKKPIGW DTDISWLTGE ELHVEVLENV PLTTHNFVRK
TFFTLAFCDF CRKLLFQGFR CQTCGYKFHQ RCSTEVPLMC VNYDQLDLLF VSKFFEHHPI PQEEASLAET ALTSGSSPSA
PASDSIGPQI LTSPSPSKSI PIPQPFRPAD EDHRNQFGQR DRSSSAPNVH INTIEPVNID DLIRDQGFRG DGGSTTGLSA
TPPASLPGSL TNVKALQKSP GPQRERKSSS SSEDRNRMKT LGRRDSSDDW EIPDGQITVG QRIGSGSFGT VYKGKWHGDV
AVKMLNVTAP TPQQLQAFKN EVGVLRKTRH VNILLFMGYS TKPQLAIVTQ WCEGSSLYHH LHIIETKFEM IKLIDIARQT
AQGMDYLHAK SIIHRDLKSN NIFLHEDLTV KIGDFGLATV KSRWSGSHQF EQLSGSILWM APEVIRMQDK NPYSFQSDVY
AFGIVLYELM TGQLPYSNIN NRDQIIFMVG RGYLSPDLSK VRSNCPKAMK RLMAECLKKK RDERPLFPQI LASIELLARS
LPKIHRSASE PSLNRAGFQT EDFSLYACAS PKTPIQAGGY GEFAAFK
```

Green: peptides identified with confidence >99%

Red: peptides identified with confidence <95%

b BRAF-ref

Serine/threonine-protein kinase B-raf

```
MAALSGGGGGGAEPGQALFNGDMEPEAGAGAGAAASSAADPAIPEEVWNIKQMIKLTQEHIEALLDKFGGEHNPPSIYLEAYEEYTS
KLDALQQREQQLLESLGNGTDFSVSSSASMDTVTSSSSSSLSVLPSSLSVFQNPTDVARSNPKSPQKPIVRVFLPNKQRTVVPARCG
VTVRDSLKKALMMRGLIPECCAVYRIQDGEKKPIGWDTDISWLTGEELHVEVLENVPLTTHNFVRKTFFTLAFCDFCRKLLFQGFRC
QTCGYKFHQRCSTEVPLMCVNYDQLDLLFVSKFFEHHPIPQEEASLAETALTSGSSPSAPASDSIGPQILTSPSPSKSIPIPQPFRP
ADEDHRNQFGQRDRSSSAPNVHINTIEPVNIDDLIRDQGFRGDGGSTTGLSATPPASLPGSLTNVKALQKSPGPQRERKSSSSSEDR
NRMKTLGRRDSSDDWEIPDGQITVGQRIGSGSFGTVYKGKWHGDVAVKMLNVTAPTPQQLQAFKNEVGVLRKTRHVNILLFMGYSTK
PQLAIVTQWCEGSSLYHHLHIIETKFEMIKLIDIARQTAQGMDYLHAKSIIHRDLKSNNIFLHEDLTVKIGDFGLATVKSRWSGSHQ
FEQLSGSILWMAPEVIRMQDKNPYSFQSDVYAFGIVLYELMTGQLPYSNINNRDQIIFMVGRGYLSPDLSKVRSNCPKAMKRLMAEC
LKKKRDERPLFPQILASIELLARSLPKIHRSASEPSLNRAGFQTEDFSLYACASPKTPIQAGGYGAFPVH
```

BRAF-X1

PREDICTED: serine/threonine-protein kinase B-raf

```
MAALSGGGGGGAEPGQALFNGDMEPEAGAGAGAAASSAADPAIPEEVWNIKQMIKLTQEHIEALLDKFGGEHNPPSIYLEAYEEYTS
KLDALQQREQQLLESLGNGTDFSVSSSASMDTVTSSSSSSLSVLPSSLSVFQNPTDVARSNPKSPQKPIVRVFLPNKQRTVVPARCG
VTVRDSLKKALMMRGLIPECCAVYRIQDGEKKPIGWDTDISWLTGEELHVEVLENVPLTTHNFVRKTFFTLAFCDFCRKLLFQGFRC
QTCGYKFHQRCSTEVPLMCVNYDQLDLLFVSKFFEHHPIPQEEASLAETALTSGSSPSAPASDSIGPQILTSPSPSKSIPIPQPFRP
ADEDHRNQFGQRDRSSSAPNVHINTIEPVNIDDLIRDQGFRGDGGSTTGLSATPPASLPGSLTNVKALQKSPGPQRERKSSSSSEDR
NRMKTLGRRDSSDDWEIPDGQITVGQRIGSGSFGTVYKGKWHGDVAVKMLNVTAPTPQQLQAFKNEVGVLRKTRHVNILLFMGYSTK
PQLAIVTQWCEGSSLYHHLHIIETKFEMIKLIDIARQTAQGMDYLHAKSIIHRDLKSNNIFLHEDLTVKIGDFGLATVKSRWSGSHQ
FEQLSGSILWMAPEVIRMQDKNPYSFQSDVYAFGIVLYELMTGQLPYSNINNRDQIIFMVGRGYLSPDLSKVRSNCPKAMKRLMAEC
LKKKRDERPLFPQILASIELLARSLPKIHRSASEPSLNRAGFQTEDFSLYACASPKTPIQAGGYGEFAAFK
```

Green: peptides identified with confidence >95%

Red: peptides identified with confidence <50%

Supplementary Figure S26. BRAF peptides identified by an LTQ-Orbitrap XL mass spectrometer (Thermo Fisher Scientific, a) and by a 5600 TripleTOF mass spectrometer (ABSciex, b) in A375 melanoma cells.

BRAF-ref

Serine/threonine-protein kinase B-raf OS=Homo sapiens GN=BRAF PE=1 SV=4 - [BRAF_HUMAN]

```
MAALSGGGGG GAEPGQALFN GDMEPEAGAG AGAAASSAAD PAIPEEVWNI KQMIKLTQEH IEALLDKFGG EHNPPSIYLE
AYEEYTSKLD ALQQREQQLL ESLGNGTDFS VSSSASMDTV TSSSSSSLSV LPSSLSVFQN PTDVARSNPK SPQKPIVRVF
LPNKQRTVVP ARCGVTVRDS LKKALMMRGL IPECCAVYRI QDGEKKPIGW DTDISWLTGE ELHVEVLENV PLTTHNFVRK
TFFTLAFCDF CRKLLFQGFR CQTCGYKFHQ RCSTEVPLMC VNYDQLDLLF VSKFFEHHPI PQEEASLAET ALTSGSSPSA
PASDSIGPQI LTSPSPSKSI PIPQPFRPAD EDHRNQFGQR DRSSSAPNVH INTIEPVNID DLIRDQGFRG DGGSTTGLSA
TPPASLPGSL TNVKALQKSP GPQRERKSSS SSEDRNRMKT LGRRDSSDDW EIPDGQITVG QRIGSGSFGT VYKGKWHGDV
AVKMLNVTAP TPQQLQAFKN EVGVLRKTRH VNILLFMGYS TKPQLAIVTQ WCEGSSLYHH LHIIETKFEM IKLIDIARQT
AQGMDYLHAK SIIHRDLKSN NIFLHEDLTV KIGDFGLATV KSRWSGSHQF EQLSGSILWM APEVIRMQDK NPYSFQSDVY
AFGIVLYELM TGQLPYSNIN NRDQIIFMVG RGYLSPDLSK VRSNCPKAMK RLMAECLKKK RDERPLFPQI LASIELLARS
LPKIHRSASE PSLNRAGFQT EDFSLYACAS PKTPIQAGGY GAFPVH
```

BRAF-X1

PREDICTED: serine/threonine-protein kinase B-raf isoform X1 [Homo sapiens]

```
MAALSGGGGG GAEPGQALFN GDMEPEAGAG AGAAASSAAD PAIPEEVWNI KQMIKLTQEH IEALLDKFGG EHNPPSIYLE
AYEEYTSKLD ALQQREQQLL ESLGNGTDFS VSSSASMDTV TSSSSSSLSV LPSSLSVFQN PTDVARSNPK SPQKPIVRVF
LPNKQRTVVP ARCGVTVRDS LKKALMMRGL IPECCAVYRI QDGEKKPIGW DTDISWLTGE ELHVEVLENV PLTTHNFVRK
TFFTLAFCDF CRKLLFQGFR CQTCGYKFHQ RCSTEVPLMC VNYDQLDLLF VSKFFEHHPI PQEEASLAET ALTSGSSPSA
PASDSIGPQI LTSPSPSKSI PIPQPFRPAD EDHRNQFGQR DRSSSAPNVH INTIEPVNID DLIRDQGFRG DGGSTTGLSA
TPPASLPGSL TNVKALQKSP GPQRERKSSS SSEDRNRMKT LGRRDSSDDW EIPDGQITVG QRIGSGSFGT VYKGKWHGDV
AVKMLNVTAP TPQQLQAFKN EVGVLRKTRH VNILLFMGYS TKPQLAIVTQ WCEGSSLYHH LHIIETKFEM IKLIDIARQT
AQGMDYLHAK SIIHRDLKSN NIFLHEDLTV KIGDFGLATV KSRWSGSHQF EQLSGSILWM APEVIRMQDK NPYSFQSDVY
AFGIVLYELM TGQLPYSNIN NRDQIIFMVG RGYLSPDLSK VRSNCPKAMK RLMAECLKKK RDERPLFPQI LASIELLARS
LPKIHRSASE PSLNRAGFQT EDFSLYACAS PKTPIQAGGY GEFAAFK
```

Green: peptides identified with confidence >99%

Yellow: peptides identified with 95%<confidence<99%

Red: peptides identified with confidence <95%

Supplementary Figure S27. BRAF peptides identified by LTQ-Orbitrap XL mass spectrometer (Thermo Fisher Scientific) in WM793B melanoma cells.

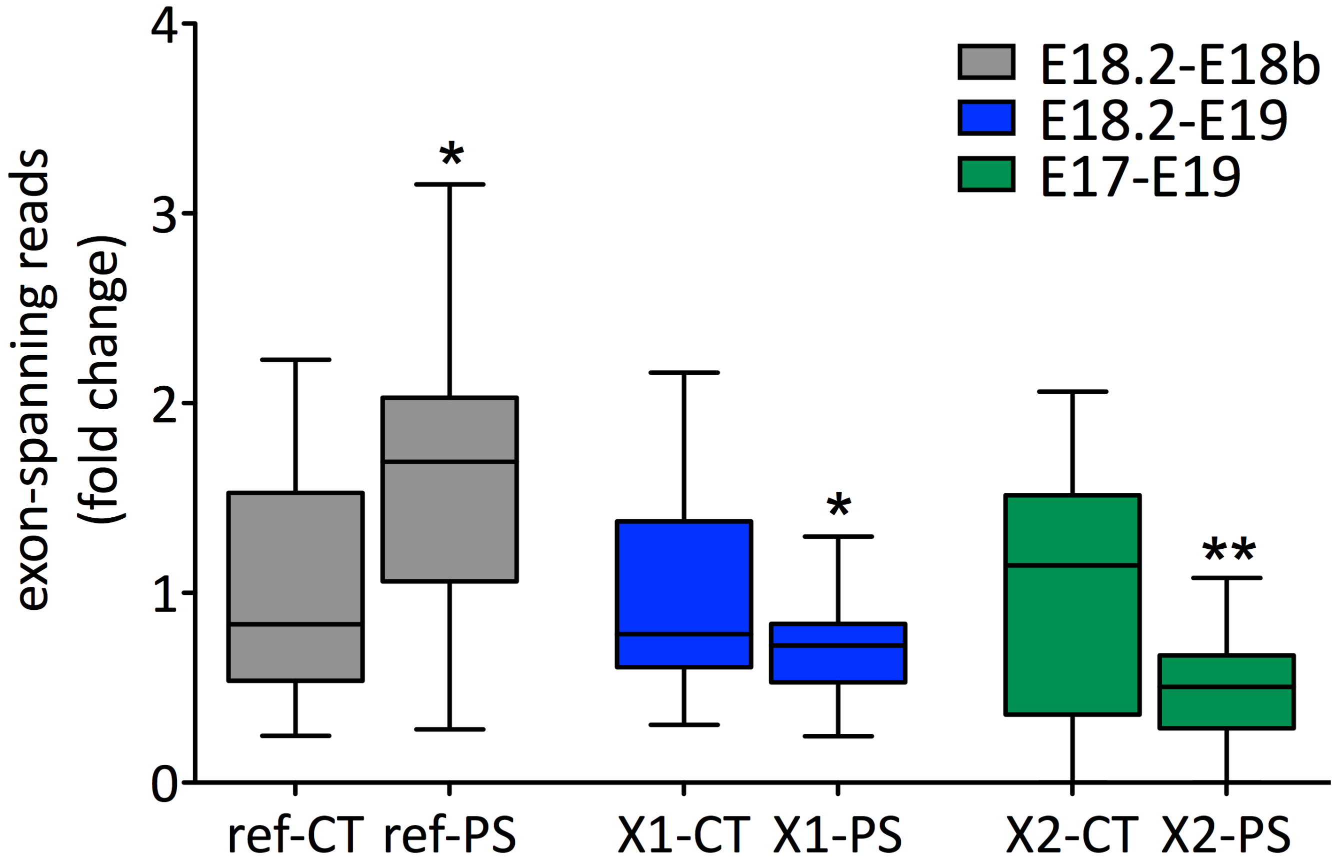

Supplementary Figure S28. Translation efficiency of reference, X1 and X2 *BRAF* mRNAs in A375 cells. The reads that span E18.2-E18b (*BRAF*-ref, grey), E18.2-E19 (*BRAF*-X1, blue), E17-E19 (*BRAF*-X2, green) were retrieved using the RNA-seq data reported in GSE64741 on n=9 whole RNA samples (GSM1579147 to GSM1579155, CT) and in n=20 RNA samples isolated using the TRAP (translating ribosome affinity purification) protocol (GSM1579156 to GSM1579163 and GSM1579176 to GSM1579187, PS). In order to account for the differences in library size among samples, they were then normalized against the reads that span E17-E18.2. Finally, normalized reads were used to compare CT and PS samples.*p<0.05, **p<0.01.

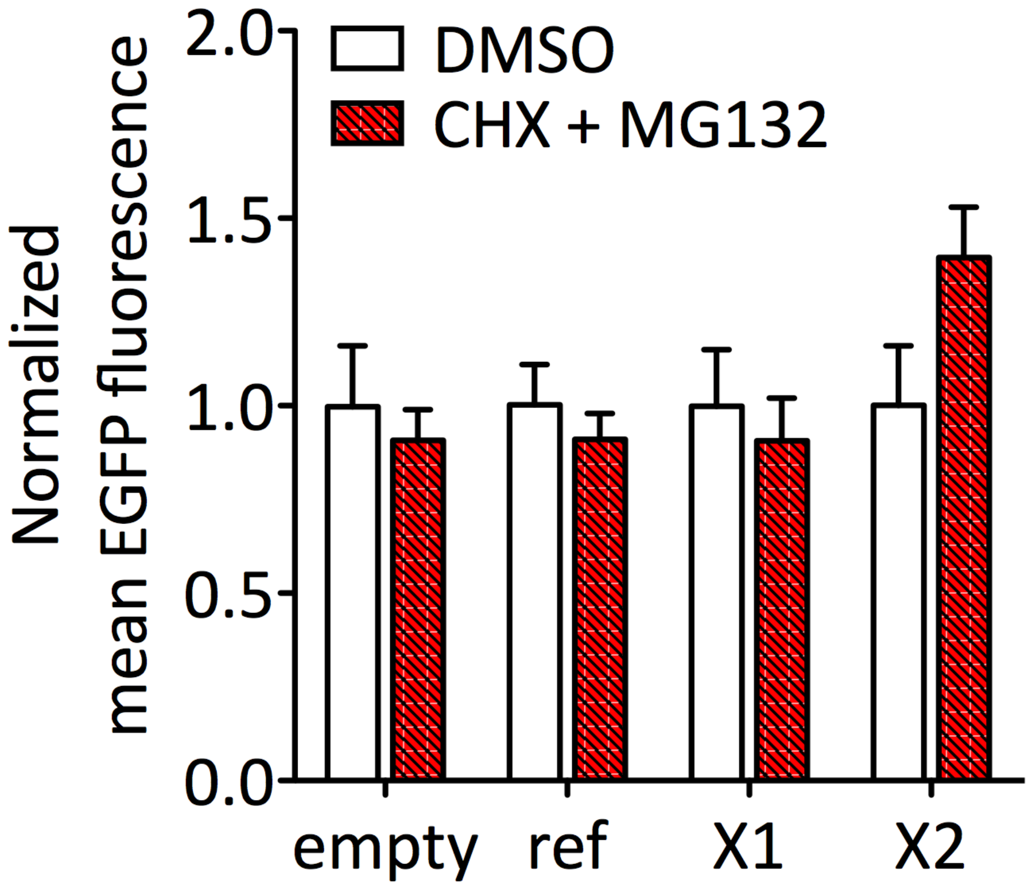

Supplementary Figure S29. BRAF-X2 protein displays a faster decay due to increased proteosomal-mediated degradation.

Upon the transient transfection of pEGFP-empty, pEGFP-CR3-ref, pEGFP-CR3-X1 and pEGFP-CR3-X2 plasmids, A375 cells were treated with 100ug/ml cicloheximide (CHX) and 20uM MG132 for 8h. The block of CR3-X2 degradation causes by MG132 remains evident also in the absence of new protein synthesis. The graph represents the mean±SEM of 3 independent experiments.
