# Supplementary material for: The landscape of BRAF transcript and protein variants in human cancer
Source: Mol Cancer. 2017 Apr 28;16:85. doi: 10.1186/s12943-017-0645-4 (PMC5410044; doi:10.1186/s12943-017-0645-4)
Supplement: Supplementary file 2 — Table S1. Coordinates of the exons of human BRAF analyzed in this study. Table S2. List of the human tumor samples used in this study. Table S3. Real-time PCR primers. Table S4. PCR primers. Table S5. siRNA sequences. (PDF 172 kb) [file 12943_2017_645_MOESM2_ESM.pdf]

Supplementary Table S1. Coordinates of the exons of human *BRAF* analyzed in this study.

Exon	GRCh38		GRCh37		length
	start	end	start	end	
**E1**	140,924,764	140,924,566	140,624,564	140,624,366	199
**E2**	140,850,212	140,850,111	140,550,012	140,549,911	102
**E3**	140,834,872	140,834,609	140,534,672	140,534,409	264
**E4**	140,808,995	140,808,892	140,508,795	140,508,692	104
**E5**	140,808,062	140,807,960	140,507,862	140,507,760	103
**E6**	140,801,560	140,801,412	140,501,360	140,501,212	149
**E7**	140,800,481	140,800,362	140,500,281	140,500,162	120
**E8**	140,794,467	140,794,308	140,494,267	140,494,108	160
**E9**	140,787,584	140,787,548	140,487,384	140,487,348	37
**E10**	140,783,157	140,783,021	140,482,957	140,482,821	137
**E11**	140,781,693	140,781,576	140,481,493	140,481,376	118
**E12**	140,778,075	140,777,991	140,477,875	140,477,791	85
**E13**	140,777,088	140,776,912	140,476,888	140,476,712	177
**E14**	140,754,233	140,754,187	140,454,033	140,453,987	47
**E15**	140,753,393	140,753,275	140,453,193	140,453,075	119
**E16**	140,749,418	140,749,287	140,449,218	140,449,087	132
**E17**	140,739,946	140,739,812	140,439,746	140,439,612	135
**E18.1**	140,734,770	140,734,012	140,434,570	140,433,812	759
**E18.2**	140,734,770	140,734,617	140,434,570	140,434,417	154
**E18.3**	140,734,770	140,734,521	140,434,570	140,434,321	250
**E18.4**	140,734,770	140,734,479	140,434,570	140,434,279	292
**E18.5**	140,734,770	140,734,597	140,434,570	140,434,397	174
**E19**	140,726,516	140,719,334	140,426,316	140,419,134	7183
**NE1**	140,749,052	140,748,932	140,448,852	140,448,732	121
**NE2**	140,747,447	140,747,366	140,447,247	140,447,166	82
**NE3**	140,716,036	140,715,951	140,415,836	140,415,751	86
**NE4**	140,749,052	140,748,995	140,448,852	140,448,795	58
**NE5**	140,808,316	140,808,237	140,508,116	140,508,037	80
**NE6**	140,834,872	140,834,061	140,534,672	140,533,861	812

Supplementary Table S2. List of the human tumor samples used in this study.

Cancer	# Sample	Sample Type				
		1	2	3	4	5
Breast Cancer	1250	1130			7	113
Colon Cancer	544	310	1		1	41
Head and Neck Cancer	551	505			2	44
Lung Adenocarcinoma	596	536	2			58
Lung Squamous Cell Carcinoma (SCC)	555	504				51
Melanoma	472	103			365	1
Thyroid Cancer	620	529			10	81
Acute Myeloid Leukemia (AML)	171			171		
Diffuse Large B-Cell Lymphoma (DLBCL)	48			48		
**Total number of samples**	**4807**					

1. primary solid tumor
2. recurrent solid tumor
3. primary blood-derived cancer
4. metastatic solid tumor
5. normal solid tissue

Supplementary Table S3. Real-time PCR primers.

Name	Sequence	Purpose
tot*BRAF* qRT-PCR F	CAGCACCTACACCTCAGCAG	real-time PCR amplification of all *BRAF* isoforms
tot*BRAF* qRT-PCR R	GATACAAGCTGGAGCCCTCA	
ref*BRAF* qRT-PCR F*	CTGTCCACTGAAACAAATGAGTG	real-time PCR amplification of *BRAF*-ref
ref*BRAF* qRT-PCR R*	CATATGTTCATTTATTTTCCTTTTGTT	
BRAF-X1 qRT-PCR F	AGTGCATCAGAACCCTCCTT	real-time PCR amplification of *BRAF*-X1
BRAF-X1 qRT-PCR R	CTTGAAGGCTGCAAATTCTC	
BRAF-X2 qRT-PCR F	AGAGACCACTCTTTCCCCAAGAGAA	real-time PCR amplification of *BRAF*-X2
BRAF-X2 qRT-PCR R	TGTTAACAAATTGTACGAACAC	
BRAF-E19-1 qRT-PCR F**	ATGGAATTTCCCATCCAACA	real-time PCR amplification of *BRAF*-X1 plus X2 (different sections of E19)
BRAF-E19-1 qRT-PCR R**	TGAAGAAACACTGGCAGCAG	
BRAF-E19-2 qRT-PCR F	TCCTAGCTTCTGGTTTGGTG	
BRAF-E19-2 qRT-PCR R	GATCTGAGGAGGATGTGCAG	
BRAF-E19-3 qRT-PCR F	CTTGATTGCACATGAAGCTG	
BRAF-E19-3 qRT-PCR R	ATGGCCAGGTCATAAAGGAT	
BRAF-E19-4 qRT-PCR F	TTATGGCGACTCACACACAC	
BRAF-E19-4 qRT-PCR R	CTCCACGAGAACCTTTTCAA	
fl*BRAF* qRT-PCR F	CCAGAGTGCTGTGCTGTTTA	real-time PCR amplification of full length *BRAF*
fl*BRAF* qRT-PCR R	CATTCTCCAACACTTCCACA	
BRAF-E2 qRT-PCR F	GGGAGCATAATCCACCATCA	real-time PCR amplification of exon 2-3 junction
BRAF-E3 qRT-PCR R	CGTTCCCCAGAGATTCCAA	
BRAF-E3 qRT-PCR F	CCCAACAAACAGAGGACAGTG	real-time PCR amplification of exon 3-4 junction
BRAF-E4a qRT-PCR R	GCACAGCACTCTGGGATTAG	
BRAF-NE6p qRT-PCR F	TCACCATTTTGCTGTTTCTTTG	real-time PCR amplification of NE6p exon
BRAF-NE6p qRT-PCR R	GGATGCCTCTATTTGCATGA	
Δ[3-10]*BRAF* qRT-PCR F	CAATATATCTGGAGAAAACACTTGG	real-time PCR amplification of Δ[3-10] *BRAF*
Δ[3-10]*BRAF* qRT-PCR R	TGAGGTGTAGGTGCTGTCAC	
GAPDH qRT-PCR F	CGCTCTCTGCTCCTCCTGTT	house keeping genes for real-time PCR
GAPDH qRT-PCR R	CCATGGTGTCTGAGCGATGT	
PBGD qRT-PCR F	TCCAAGCGGAGCCATGTCTG	
PBGD qRT-PCR R	AGAATCTTGTCCCCTGTGGTGGA	
SDHA qRT-PCR F	CCACTCGCTATTGCACACC	
SDHA qRT-PCR R	CACTCCCCATTCTCCATCA	

* real-time PCR primers used for the detection of the reference (canonical) 3'UTR of *BRAF* in Marranci et al., JID 2015

** real-time PCR primers used for the detection of the X1 3'UTR of *BRAF* in Marranci et al., JID 2015

Supplementary Table S4. PCR primers.

**Name**	**Sequence**	**Purpose**
BRAF-E19-1 F	ATGGAATTTCCCATCCAACA	PCR amplification of *BRAF*-X1 plus X2 (different sections of E19)
BRAF-E19-1 R	CTGGACCCAATGAGAAAGA	
BRAF-E19-2 F	GGCTGTGTTCCCTTTACCAA	
BRAF-E19-2 R	CAGTGACGCAGCCACATACT	
BRAF-E19-3 F	CATGAAGCTGGACTGCGTTA	
BRAF-E19-3 R	CTAATTGCCCCATGCATTTT	
BRAF-E19-4 F	TGACCGCTGGTTCTCATGTA	
BRAF-E19-4 R	AGAAGGGGACAGCACAGAGA	
BRAF-E7 F	AGGCGTCCTTAGCAGAGACT	PCR amplification of *BRAF*-X1 and X2 CDS
BRAF-E8 F	AAATCCATTCCAATTCCACA	
BRAF-E9-10 F	ATTTCGTGGTGATGGAGGAT	
BRAF-E19-1 qRT-PCR R	TGAAGAAACACTGGCAGCAG	
BRAF-E1/2 F*	CGGAGGAGGTGTGGAATATC	PCR amplification of *BRAF*-004 transcript variant
BRAF-E4b R	CCTCTCATCATCAGTGCTTTCTT	
BRAF-E1/2 F*	CGGAGGAGGTGTGGAATATC	PCR amplification of the CDS of Δ[3-10] *BRAF* transcript variants
ref*BRAF*-STOP R	TCAGTGGACAGGAAACGCACCATATC	
BRAF-X1-STOP R	CTACTTGAAGGCTGCAAATTCTC	
Mut X2 Lys>Arg F	GTTCGTACAATTTGTTAACATCAGAACACAGTTCTGTTCCTCAAATC	mutagenesis of Lysine 739 residue to Arg within BRAF-X2 C-term
Mut X2 Lys>Arg R	GATTTGAGGAACAGAACTGTGTTCTGATGTTAACAAATTGTACGAAC	

Supplementary Table S5. siRNA sequences.

**Name**	**Sequence**	**Purpose**
si-tot*BRAF*	AGAAUUGGAUCUGGAUCAUUU	knock-down of all *BRAF* isoforms
si-ref*BRAF*	UCCUGUCCACUGAAACAAA	knock-down of *BRAF*-ref
si-*BRAF*-E19-1	UGCUGCCAGUGUUUCUUCA	knock-down of *BRAF*-X1 plus X2
si-*BRAF*-E19-2	AGGGCAGUGUCUUACAAAA	knock-down of *BRAF*-X1 plus X2
si-*BRAF*-E19-3	AUGAUGGGCAGCAAUCAUGUAUU	knock-down of *BRAF*-X1 plus X2
si-fl*BRAF*	CUGUCAAACAUGUGGUUAU	knock-down of fl*BRAF*
si-Δ[3-10]*BRAF*	UGGAGAAAACACUUGGUAG	knock-down of Δ[3-10]*BRAF*
si-CT	CUCCGAACGUGUCACGUU	control siRNA
