# Supplementary material for: The landscape of BRAF transcript and protein variants in human cancer
Source: Mol Cancer. 2017 Apr 28;16:85. doi: 10.1186/s12943-017-0645-4 (PMC5410044; doi:10.1186/s12943-017-0645-4)
Supplement: Supplementary file 3 — Primers and siRNAs location along BRAF-ref, BRAF-X1, and BRAF-X2 mRNAs. (PDF 67 kb) [file 12943_2017_645_MOESM3_ESM.pdf]

Supplementary Notes
Primers and siRNAs location along *BRAF*-ref, *BRAF*-X1 and *BRAF*-X2 mRNAs.

***BRAF*-ref sequence starting from ATG**
ATGGCGGCGCTGAGCGGTGGCGGTGGTGGCGGCGCGGAGCCGGGCCAGGCTCTGTTCAACGGGGACATGGAG
CCCGAGGCCGGCGCCGGCGCCGGCGCCGCGGCCTCTTCGGCTGCGGACCCTGCCATTCCGGAGGAGGTGTGG
AATATCAAACAAATGATTAAGTTGACACAGGAACATATAGAGGCCCTATTGGACAAATTTGGTGGGGAGCAT
AATCCACCATCAATATATCTGGAGGCCTATGAAGAATACACCAGCAAGCTAGATGCACTCCAACAAAGAGAA
CAACAGTTATTGGAATCTCTGGGGAACGGAACTGATTTTTCTGTTTCTAGCTCTGCATCAATGGATACCGTT
ACATCTTCTTCCTCTTCTAGCCTTTCAGTGCTACCTTCATCTCTTTCAGTTTTTCAAAATCCCACAGATGTG
GCACGGAGCAACCCCAAGTCACCACAAAAACCTATCGTTAGAGTCTTCCTGCCCAACAAACAGAGGACAGTG
GTACCTGCAAGGTGTGGAGTTACAGTCCGAGACAGTCTAAAGAAAGCACTGATGATGAGAGGTCTAATCCCA
GAGTGCTGTGCTGTTTACAGAATTCAGGATGGAGAGAAGAAACCAATTGGTTGGGACACTGATATTTCCTGG
CTTACTGGAGAAGAATTGCATGTGGAAGTGTTGGAGAATGTTCCACTTACAACACACAACTTTGTACGAAAA
ACGTTTTTCACCTTAGCATTTTGTGACTTTTGTCGAAAGCTGCTTTTCCAGGGTTTCCGCTGTCAAACATGT
GGTTATAAATTTCACCAGCGTTGTAGTACAGAAGTTCCACTGATGTGTGTTAATTATGACCAACTTGATTTG
CTGTTTGTCTCCAAGTTCTTTGAACACCACCCAATACCACAGGAAG**AGGCGTCCTTAGCAGAGACT**GCCCTA
ACATCTGGATCATCCCCTTCCGCACCCGCCTCGGACTCTATTGGGCCCCAAATTCTCACCAGTCCGTCTCCT
TCA**AAATCCATTCCAATTCCACA**GCCCTTCCGACCAGCAGATGAAGATCATCGAAATCAATTTGGGCAACGA
GACCGATCCTCATCAGCTCCCAATGTGCATATAAACACAATAGAACCTGTCAATATTGATGACTTGATTAGA
GACCAAGG**ATTTCGTGGTGATGGAGGAT**CAACCACAGGTTTGTCTGCTACCCCCCCTGCCTCATTACCTGGC
TCACTAACTAACGTGAAAGCCTTACAGAAATCTCCAGGACCTCAGCGAGAAAGGAAGTCATCTTCATCCTCA
GAAGACAGGAATCGAATGAAAACACTTGGTAGACGGGACTCGAGTGATGATTGGGAGATTCCTGATGGGCAG
ATTACAGTGGGACAAAGAATTGGATCTGGATCATTTGGAACAGTCTACAAGGGAAAGTGGCATGGTGATGTG
GCAGTGAAAATGTTGAATGTGA**CAGCACCTACACCTCAGCAG**TTACAAGCCTTCAAAAATGAAGTAGGAGTA
CTCAGGAAAACACGACATGTGAATATCCTACTCTTCATGGGCTATTCCACAAAGCCACAACTGGCTATTGTT
ACCCAGTGGTG**TGAGGGCTCCAGCTTGTATC**ACCATCTCCATATCATTGAGACCAAATTTGAGATGATCAAA
CTTATAGATATTGCACGACAGACTGCACAGGGCATGGATTACTTACACGCCAAGTCAATCATCCACAGAGAC
CTCAAGAGTAATAATATATTTCTTCATGAAGACCTCACAGTAAAAATAGGTGATTTTGGTCTAGCTACAGTG
AAATCTCGATGGAGTGGGTCCCATCAGTTTGAACAGTTGTCTGGATCCATTTTGTGGATGGCACCAGAAGTC
ATCAGAATGCAAGATAAAAATCCATACAGCTTTCAGTCAGATGTATATGCATTTGGAATTGTTCTGTATGAA
TTGATGACTGGACAGTTACCTTATTCAAACATCAACAACAGGGACCAGATAATTTTTATGGTGGGACGAGGA
TACCTGTCTCCAGATCTCAGTAAGGTACGGAGTAACTGTCCAAAAGCCATGAAGAGATTAATGGCAGAGTGC
CTCAAAAAGAAAAGAGATGAGAGACCACTCTTTCCCCAA
ATTCTCGCCTCTATTGAGCTGCTGGCCCGCTCATTGCCAAAAATTCACCGCAGTGCATCAGAACCCTCCTTG
AATCGGGCTGGTTTCCAAACAGAGGATTTTAGTCTATATGCTTGTGCTTCTCCAAAAACACCCATCCAGGCA
GGGGGATATGGTGCGTTTC**CTGTCCACTGAAACAAATGAGTG**AGAGAGTTCAGGAGAGTAGC**AACAAAAGGA**
AAATAAATGAACATATGTTT…

START codon
Exon 1-17
Exon 18.3
STOP codon

si-tot*BRAF*
si-fl*BRAF*
si-ref*BRAF*

primers tot*BRAF* qRT-PCR F/R: 153bp
primers ref*BRAF* qRT-PCR F/R: 70bp
primers fl*BRAF* qRT-PCR F/R: 115bp

***BRAF*-X1 sequence starting from ATG**

ATGGCGGCGCTGAGCGGTGGCGGTGGTGGCGGCGCGGAGCCGGGCCAGGCTCTGTTCAACGGGGACATGGAG
CCCGAGGCCGGCGCCGGCGCCGGCGCCGCGGCCTCTTCGGCTGCGGACCCTGCCATTCCGGAGGAGGTGTGG
AATATCAAACAAATGATTAAGTTGACACAGGAACATATAGAGGCCCTATTGGACAAATTTGGTGGGGAGCAT
AATCCACCATCAATATATCTGGAGGCCTATGAAGAATACACCAGCAAGCTAGATGCACTCCAACAAAGAGAA
CAACAGTTATTGGAATCTCTGGGGAACGGAACTGATTTTTCTGTTTCTAGCTCTGCATCAATGGATACCGTT
ACATCTTCTTCCTCTTCTAGCCTTTCAGTGCTACCTTCATCTCTTTCAGTTTTTCAAAATCCCACAGATGTG
GCACGGAGCAACCCCAAGTCACCACAAAAACCTATCGTTAGAGTCTTCCTGCCCAACAAACAGAGGACAGTG
GTACCTGCAAGGTGTGGAGTTACAGTCCGAGACAGTCTAAAGAAAGCACTGATGATGAGAGGTCTAATCCCA
GAGTGCTGTGCTGTTTACAGAATTCAGGATGGAGAGAAGAAACCAATTGGTTGGGACACTGATATTTCCTGG
CTTACTGGAGAAGAATTGCATGTGGAAGTGTTGGAGAATGTTCCACTTACAACACACAACTTTGTACGAAAA
ACGTTTTTCACCTTAGCATTTTGTGACTTTTGTCGAAAGCTGCTTTTCCAGGGTTTCCGCTGTCAAACATGT
GGTTATAAATTTCACCAGCGTTGTAGTACAGAAGTTCCACTGATGTGTGTTAATTATGACCAACTTGATTTG
CTGTTTGTCTCCAAGTTCTTTGAACACCACCCAATACCACAGGAAG**AGGCGTCCTTAGCAGAGACT**GCCCTA
ACATCTGGATCATCCCCTTCCGCACCCGCCTCGGACTCTATTGGGCCCCAAATTCTCACCAGTCCGTCTCCT
TCA**AAATCCATTCCAATTCCACA**GCCCTTCCGACCAGCAGATGAAGATCATCGAAATCAATTTGGGCAACGA
GACCGATCCTCATCAGCTCCCAATGTGCATATAAACACAATAGAACCTGTCAATATTGATGACTTGATTAGA
GACCAAGG**ATTTCGTGGTGATGGAGGAT**CAACCACAGGTTTGTCTGCTACCCCCCCTGCCTCATTACCTGGC
TCACTAACTAACGTGAAAGCCTTACAGAAATCTCCAGGACCTCAGCGAGAAAGGAAGTCATCTTCATCCTCA
GAAGACAGGAATCGAATGAAAACACTTGGTAGACGGGACTCGAGTGATGATTGGGAGATTCCTGATGGGCAG
ATTACAGTGGGACAAAGAATTGGATCTGGATCATTTGGAACAGTCTACAAGGGAAAGTGGCATGGTGATGTG
GCAGTGAAAATGTTGAATGTGA**CAGCACCTACACCTCAGCAG**TTACAAGCCTTCAAAAATGAAGTAGGAGTA
CTCAGGAAAACACGACATGTGAATATCCTACTCTTCATGGGCTATTCCACAAAGCCACAACTGGCTATTGTT
ACCCAGTGGTG**TGAGGGCTCCAGCTTGTATC**ACCATCTCCATATCATTGAGACCAAATTTGAGATGATCAAA
CTTATAGATATTGCACGACAGACTGCACAGGGCATGGATTACTTACACGCCAAGTCAATCATCCACAGAGAC
CTCAAGAGTAATAATATATTTCTTCATGAAGACCTCACAGTAAAAATAGGTGATTTTGGTCTAGCTACAGTG
AAATCTCGATGGAGTGGGTCCCATCAGTTTGAACAGTTGTCTGGATCCATTTTGTGGATGGCACCAGAAGTC
ATCAGAATGCAAGATAAAAATCCATACAGCTTTCAGTCAGATGTATATGCATTTGGAATTGTTCTGTATGAA
TTGATGACTGGACAGTTACCTTATTCAAACATCAACAACAGGGACCAGATAATTTTTATGGTGGGACGAGGA
TACCTGTCTCCAGATCTCAGTAAGGTACGGAGTAACTGTCCAAAAGCCATGAAGAGATTAATGGCAGAGTGC
CTCAAAAAGAAAAGAGATGAGAGACCACTCTTTCCCCAA

ATTCTCGCCTCTATTGAGCTGCTGGCCCGCTCATTGCCAAAAATTCACCGCAGTGCATCAGAACCCTCCTTG
AATCGGGCTGGTTTCCAAACAGAGGATTTTAGTCTATATGCTTGTGCTTCTCCAAAAACACCCATCCAGGCA
GGGGGATATG

GAGAATTTGCAGCCTTCAAG**TAG**CCACCATCATGGCAGCATCTGCTCTTATTTCTTAAGTCTTGTGTTCGTA
CAATTTGTTAACATCAAAACACAGTTCTGTTCCTCAAATCTTTTTTAAAGATACAAAATTTCCAATGCATA
AGCTGATGTGGAACAGA**ATGGAATTTCCCATCCAACA**AAAGAGGAAAGAATGTTTTAGGAACCAGAATTCT**C**
TGCTGCCAGTGTTTCTTCAACAAAAATACCACGAGCATACAAGTCTGCCCAGTCCCAGGAAGAAAGAGGAGA
GACCCTGAATTCTGACCTTTTGATGGTCAGGCATGATGGAAAGAAACTGCTGCTACAGCTTGGGAGATTTGC
TATGGAAAGTCTGCCAGTCAACTTTGCCCTTCTAACCACCAGATCAATTTGTGGCTGATCATCTGATGGGGC
AGTTTCAATCACCAAGCATCGTTCTCTTTCCTGTTCTGGAATTTTGTTTTGGAGCTCTTTCCCCTAGTGACC
ACCAGTTAGTTTCTGAGGGATGGAACAAAAATGCAGCTTGCCCTTTCTATGTGGTGCGTGTTCAGGCCTTGA
CAGATTTTATCAAAAGGAAACTATTTTATTTAAATGGAGGCTGAGTGGTGAGTAGATGTGTCTTGGTATGGA
GGAAAAGGGCATGCTGCATCTTCTTCCTGACCTCCGGGGTCTCTGGCCTTTTGTTTCCTTGCTCACTGAGGG
GTCTGTCTAACCAAGCAGGCTAGATAGTGCTGGCACACATTGCCTTCTTTCTCATTGGGTCCAGCAATGAAG
ATAAGTGTTTGGGTTTTTTTTTTTTCCTCCACAATGTAGCAAATTCTCAGGAAATACAGTTTATATCTTCCT
CCTATGCTCTTCCAGTCACCAACTACTTATGCGGCTACTTTGTCCAGGGCACAAAATGCCGTGGCAGTATCT
AACTAAACCCCCACAAAACTGCTTAATAACAGTTTTGAATGTGAGAAATTTAGATAATTTAAATATAAGGTA
CAGGTTTTAATTTCTGAGTTTCTTCTTTTCTATTTTTATTAAAAAGAAAATAATTTTCAGATTTAATTGAAT
TGGAAAAAAACAATACTTCCCACCAGAATTATATATCCTGAAAATTGTATTTTTGTTATATAAACAACTTTT
AAGAAAGATCATTATCCTTTTCTCTACCTAAATATGAGGAGTCTTAGCATAATGACAAATATTTATAATTTT
TCAATTAATGGTACTTGCTGGATCCACACTAACATCTTTGCTAATAATCTCATTGTTTCTTCCAACTGATTC
CTAACACTATATCCCACATCTTCTTTCTAGTCTTTTATCTAGAATATGCAACCTAAAATAAAAATGGTGGCG
TCTCCATTCATTCTCCTTCTTCCTTTTTTCCCAAGCCTGGTCTTCAAAAGGTTGGGCAATTTGGCAGCTGAA
TTCCCAGACAGAGAATAGAGCAATTTTAGGGATATTAGGACTGAGGGAGGGTGTGGGAAAGCTGTCATCAGT
TGTTTTTATAGAAAGAACTGGCATTCATTAAGAACCTAAATCTTATCTTTGCACAAATGGAAAATATAACCT
AGTTATAGCTTCCTTTGGCCTTTATTAAAGGGTAATATCAATCACAGTCATAGCAAAGAAAGCGGATGTATT
AATGGCAAATTAATGGAAAACCTCCCTTATCAGGAATCTAGACTCAGAATTTAGGAACACAAATCAAATCAG

ACCAACCAAGCTATAGCCAAGGACTTGAAAGAAATTAAACAAGACCCAGAATAAATCAAGGAATTAGAAATT
GTTATTTAAAAATTTCAGATTGTAACTCCAGGCCCTGCTGTCTATATTGCAGCCACTAAAAGCTCACTACCA
TTAGATTTTTGCTAACATACATGTATTCAGAAGAAAGCCTATTGAAATTTTCATTGTCTTGTAAAAGGTTGT
CCTAGTAAAATGGAAAAGATCCTTAAGTTATTAATCAGTTTGAAAAGCAAATTTGTTTTTAAGTTTTACATC
AGCAGGGCAGTGTCTTACAAAATTCAGAAATTGCAAAGGTGGAAATAATTCACGCTGATTTGAAGAACATCT
TCTGTGCAATAATACTGCCTCTCTTGAAAAGCATTGGCTGTTTTTTCTTTTTAAATATATCTCTAGATGCTT
TTAAATGTGGCTGTGTTCCCTTTACCAAGATTGGCTTCAAGTTTCCGCAGGTAGAGAGACCTGGGCTTGAAC
AAGAGGATGTGTTTCATGTCCTGCTGAGGAGGTAGAACATGTGCAGCCTGGGTCCGGGACTGCCTCCGTGGG
GCAGGGGCAGGGGCGGTACCATTAGGGAGGAAGCTTAGCATTTCAGTTTCTTAAACAATATTCAGGGTGATA
CACTTTTTCTTCCCTTGCATTTTAGAATAGGCTGGTATCTCATTTGAACGGGGGAGCAGACTTGATCTCAAA
TGAAGCTGTGCCCAGGAGCCAGGCTTAGCATATTGAGATTTTTATAGATACCTTAAAAAATAAAATATTTAA
ACCTCTCTTTTCTTCCTTTTTCTATGAAATAGGTTTTTTCTCTAGTTTACAAATGACATGAAAATAGGTTTT
ATTTGTGTTTTATCTGCTTTATTTTTTGATGCTTAGACAACAGTTAGACTTACTGAGCTCCTAAAAAAACGA
GGAAGAAGTCCTTATTTGTGAAAAGCACTTTATGAGTAATTGTATAGACAGTATGTGGCTGCGTCACTGATC
ATCTTGTAAGGGTGTAACAGTCTTGTCTGTAAAGTGGCTGCAGTGCCTTCTGTAGTGTGTTTTATTTTTGGT
AGGGAGAGGTGAAGCCTTCTGAAAAATTTGAGAGCAACTACAGAGGATTGTTTGTAACTGTGTAGTATTCCT
GATGGACTTTTTTCATCGTTAGAGTCAAGGACCTAGACTTTTGCCACTGAAATAATATTGACCAAAAAAATA
GTTTATAAAAGGGATTTGTGAATAGAAAATTCAGTGTGATCATTTGTTGTTAATGTGCACCTTAAAAGAAGA
TTCTGTCTAGCTGTCAAATTCTGGTTCCCGAATATCTCACCCCTGATTGTATTTGAGATCTAGTAGGGCATA
CTGGGGCATTTTAGAAGATAAAATCCCATACAAATGATATATGCTATATTTATGTTGGTGTTGGAGAAGAAA
GAGCAGTATATAAAGAAATAATTCAAGACTGCAGCACTGTCAACCTGAAACTTTGTAAATATT**TCCTAGCTT**
CTGGTTTGGTGCGGTGACAGCACTTTCATCACAGGATGTTACCTTGTATTCACCAGGCGGAGTGCGAGCTG**C**
TGCACATCCTCCTCAGATCTCACCTGTCCCCACTGTACATCCACCCGCCAGCTGCTTGCAAACCTCATCTCT
AGCTTTAGTTCGAAACCACATTGCAGGGTTCAGGTGACCTCTACAAAAAACTACCTCTTCAGAATGAGGTAA
TGAATAGTTATTTATTTTAAAATATGAAAAGTCAGGAGCTCTAGAACATGACGATGATTTAAGATTTTAACT
TTTTTGTGTACTTGTATTTGAGCACTCTCATTTTGTCCTAAAGGGCATTATACATTTAAGCAGTAATACTGT
AAAAAAATGTGTTGCTCGGAATATCTGAATGTTGTTGAAAGTGGTGCCAGAACCGGTTTAGGGGTACGTTTC
AGAATCTTAACCTTGAGTCAATTGCATGAAATTAAATAGCTGTGGTATCACTTCACTAACAGTGATGTAATT
TTAATTTTCAGTAGGCTTGGCATGACAGTACATCCTCATAATGAGTTTGCTGCAGCTTTGTCACATGCACAG
GCATTCATAGAAAGACCACCCAGCTAAGAGGGTAGAATGATTACTCTTTTTGCAAGATTCTCTTCTTTGTCC
AAGTTGGCATTGTTAGTGCTAGGAATACCAGCACCTTGAGACGAGCAGATTCCAACCATTAGGCTATAAACA
CCATAGCCAGAGATGGAAGGTTTACTGTGAGTATGAACAGCAAATAGCTTACAGGTCATGAGTTGAAATGGT
GTAGGTGAGGCTCTAGAAAAATACCTTGACAATTTGCCAAATGATCTTACTGTGCCTTCATGATGCAATAAA
AAAGCTAACATTTTAGCAGAAATCAGTGATTTGTGAAGAGAGCAGCCACTCTGGTTTAACTCAGCTGTGTTA
ATAATTTTTAGAGTGCAATTTAGACTGCATAGGTAAATGCACTAAAGAGTTTATAGCCAAAATCACATTTAA
CAATGAGAAAACACACAGGTAAATTTTCAGTGAACAAAATTATTTTTTTAAAGCACATAATCCCTAGTATAG
TCAGATATATTTATCACATAGAGCAACTAGGTTGCAAATATAGTTCAGTGACATTTCTAGAGAAACTTTTTC
TACTCCCATAGGCTCTTCAAAGCATGGAACTTTTATACAACAGAAATGTTGACAGAAATTGCTGTAGTTTAG
GGTTGAAGTACTGTATGATGGGCAGCAATCATGTATTAACTTAGAAGGGGAAATTGAAATATAGGACCGAAT
TTGGTTTTATCAGTTTCCAGAGTACTGCTGCCAACCTAGACACTGATTTTTCAGAGTTTGAAATGTAAATTT
CTTCCCGGGA**CTTGATTGCACATGAAGCTG**GACTGCGTTAGTCATCCTGTCCCAAAGCGCTGTGGGGGCCAG
GGTGGAGGTCTCAAGGC**ATCCTTTATGACCTGGCCAT**TGGATGTAAAAGAAAACATATTCCATGCTGTGGTT
CTTGTATCTTGTTTCATTCCTCACCATTGAAAGAGAAAGTCCATGTATTGTCTCCAGCACATCCTTGAAATG
TTATACTGGGATGGATTACTGATGCCCATCGGTAGTTGAGCCCCAGAAGAGGGTAGTAGCATCTCTGCCTCA
GGTGATGATTTGTAGCTTGGCCAGAGGAGAGCGGAGTCACCAGTATATCTGTGGTCCATGTTGCTAGCTCTG
GTAAAATTAAAAATACTGGTAAGATGTTTGTTTTATTAGTACACTAGACAGTAAGCTCTGTTTTGTTGTTTT
CAAATAACCTATTTTCACTTTTGTTTGGGCAAAGACATTTAAATTGAAATTCAATTCTAATTTTTGTTAATT
GTGGAAAGGGTAATTAACAGTTCCTATCAGGTATTTTTAATGTGGAAAAGGACAGAAACCCAACTCCTAAAA
TCTTAAATTAAGGTAACAGTGCTTTAAAAAAAAAAAATGCATGGGGCAATTAGTCGGCAACTCAATGAGTGA
CTAAAGTACTTTTATTTAACATCCACAACTTCAACTGTTAAGTTTTATTAATTACTAAATCAGCTTTATTAA
AATGTTGACATTTATTTAGCTATTTTGAATAATTATAGTGACTTGACGAGTGTGTATGAGGACACAGCCAAT
GTAAGCCAGTGTATCCATTTTTTAGAGGTGCATTTTTTTTTAAAGAATTCTGTAGATAGAAGTGCTCTGAAA
ACAACTAAAATATGTTTATTCATGGTAGTATCAAAAAATGTTTGTACAAACCATCTGCTTCTCCCGGCCAGC
CGAGTTCATTCTCCAGCACCGTGACCGCTGGTTCTCATGTACAGCACATATGCGGGAGAGTTGGCAGAAAAT
TTGTGAAGAGATGCCGCAAAGGAAGGGTCTGTTGACGGGTGGGATTGGGGGTTTTGATGAAGTTGCTTAGTC
CTGGTTTTGTTTTGAAAATTACTGCGTTGCATTTTTGTGTTAAGTTTTTGAACCCACGTGTGTTTTGGTGGA
GTATGAGTTGGAAGTCACTGCAAACTAGCATAAACAACAAAGCTCACAGAGTAGGCACAGATGTAGAGAACA
GAGACCAAAATGGGGTGAGGTGGCAGTAAATCTAGGATAGGGAAAAATTAATGTGAGGGTGGGAAATAAACT

GTAATTACCTGAAATCAAATGTAAGAGTGCAATAAGTATGCTTTTTATTCTAAGCTGTGAACGGTTTTTTTA
AGAATCATTCCTTCCTAATACATTTGTGTATGTTCCATAGCTGATTAAAACCAGCTATATCAACATATAATG
CCTTTTTATTCATGTTAATGACCAACGTAAGTGGCTAGCCTTTATGTCTTATTTATCTTCATGTTATGTTAG
TTTACATACAGGGGTGTATGTCTCTGTGCTGTCCCCTTCTCCTGCCTTCATTTTAAAATGCATCCATGGGTC
CTCCGTGTTTCCTTTGGCCATGCCACATATATAGACTCAGTTTGGCCTTCATGATATCGCCTGATTTTTGAG
GACTGTATCACAGTGATATGTATTTGTGGTAATCTCATTTGTTGGTTGTACATCTGATCCTTTCCTCAACAT
GGCAATTGCTGCCTTTCCTAAGATAGGATCATACAACTGATCAGGGGATTGAATTTGATCATTCATCAACAT
GTGTCTCTGAATTTTATTCAGTAGTTGTCATTGCTCTTTGGTTTAGACCAAGAAAAAGGAAATCCCCCCTTT
TCATGTATTCCTTGGTTTGAGGACATGACTCCTGTAAGGGAGAGGAAAGGGAGATGCTTCCTGTTTGAACTG
CAGTGAATTCACGGTTCCTGTTTCACCACTCCAAACC**TTATGGCGACTCACACACAC**ATTCCTCTTTTCTGT
TACTGCCAAAGGTTCGGGTTTAGTACACTTCAGTTCCACTCAAGCA**TTGAAAAGGTTCTCGTGGAG**TCTGGG
GCGTGCCCAGTGAAAAGATGGGGACTTTTTAATTGTCCACAGACCTCTCTATACCTGCTTTGCAAAAATTAC
AATGGAGTAACTATTTTTAAAGCTTATTTTTCAATTCATAAAAAAGACATTTATTTTCAGTCAAATGGATGA
TGTCTCCCTCTTTTCCCCTATTCTCAATGTTTGCTTGAATCTTTTATTATTTTTTTTAATTCTCCCCCATAC
CCACTTCCTGATACTTTGGTTCTCTTTCCTGCTCAGGTCCCTTCATTTGTACTTTGGAGTTTTTCTCATGTA
AATTTGTATAACAGAAAATATTGTTCAGTTTGGATAGAAAGCATGGAGAATAAAAAAAGATAGCTGAAATTC
AGATTGAAGAAATTTATTTCTGTGTAAAGTTATTTAAAAACTGTATTATATAAAAGGCAAAAAAAGTTCTAT
GTACTTGATGTGAATATGCGAATACTGCTATAATAAAGATTGACTGCATGGAGAA

START codon
Exon 1-17
Exon 18.2
Exon 19
STOP codon

si-tot*BRAF*
si-fl*BRAF*
si-*BRAF*-E19-1/2/3

primers tot*BRAF* qRT-PCR F/R: 153bp
primers *BRAF*-X1 qRT-PCR F/R: 123bp
primers *BRAF*-E19-1/2/3/4 qRT-PCR, 4 F/R pairs: 74, 100, 99, 101bp
primers fl*BRAF* qRT-PCR F/R: 115bp

primers *BRAF*-E7, E8, E9-10
used with *BRAF*-E19-1 qRT-PCR R:
1606, 1505, 1356bp

primers *BRAF*-E19-1/2/3/4: 4 F/R pairs, 623, 565, 609, 595bp
BRAF-E19-1 F coincides with *BRAF*-E19-1 qRT-PCR F

BRAF-X2 sequence starting from ATG

ATGGCGGCGCTGAGCGGTGGCGGTGGTGGCGGCGCGGAGCCGGGCCAGGCTCTGTTCAACGGGGACATGGAG
CCCGAGGCCGGCGCCGGCGCCGGCGCCGCGGCCTCTTCGGCTGCGGACCCTGCCATTCCGGAGGAGGTGTGG
AATATCAAACAAATGATTAAGTTGACACAGGAACATATAGAGGCCCTATTGGACAAATTTGGTGGGGAGCAT
AATCCACCATCAATATATCTGGAGGCCTATGAAGAATACACCAGCAAGCTAGATGCACTCCAACAAAGAGAA
CAACAGTTATTGGAATCTCTGGGGAACGGAACTGATTTTTCTGTTTCTAGCTCTGCATCAATGGATACCGTT
ACATCTTCTTCCTCTTCTAGCCTTTCAGTGCTACCTTCATCTCTTTCAGTTTTTCAAAATCCCACAGATGTG
GCACGGAGCAACCCCAAGTCACCACAAAAACCTATCGTTAGAGTCTTCCTGCCCAACAAACAGAGGACAGTG
GTACCTGCAAGGTGTGGAGTTACAGTCCGAGACAGTCTAAAGAAAGCACTGATGATGAGAGGTCTAATCCCA
GAGTGCTGTGCTGTTTACAGAATTCAGGATGGAGAGAAGAAACCAATTGGTTGGGACACTGATATTTCCTGG
CTTACTGGAGAAGAATTGCATGTGGAAGTGTTGGAGAATGTTCCACTTACAACACACAACTTTGTACGAAAA
ACGTTTTTCACCTTAGCATTTTGTGACTTTTGTCGAAAGCTGCTTTTCCAGGGTTTCCGCTGTCAAACATGT
GGTTATAAATTTCACCAGCGTTGTAGTACAGAAGTTCCACTGATGTGTGTTAATTATGACCAACTTGATTTG
CTGTTTGTCTCCAAGTTCTTTGAACACCACCCAATACCACAGGAAG**AGGCGTCCTTAGCAGAGACT**GCCCTA
ACATCTGGATCATCCCCTTCCGCACCCGCCTCGGACTCTATTGGGCCCCAAATTCTCACCAGTCCGTCTCCT
TCA**AAATCCATTCCAATTCCACA**GCCCTTCCGACCAGCAGATGAAGATCATCGAAATCAATTTGGGCAACGA
GACCGATCCTCATCAGCTCCCAATGTGCATATAAACACAATAGAACCTGTCAATATTGATGACTTGATTAGA
GACCAAGG**ATTTCGTGGTGATGGAGGAT**CAACCACAGGTTTGTCTGCTACCCCCCCTGCCTCATTACCTGGC
TCACTAACTAACGTGAAAGCCTTACAGAAATCTCCAGGACCTCAGCGAGAAAGGAAGTCATCTTCATCCTCA
GAAGACAGGAATCGAATGAAAACACTTGGTAGACGGGACTCGAGTGATGATTGGGAGATTCCTGATGGGCAG
ATTACAGTGGGACAAAGAATTGGATCTGGATCATTTGGAACAGTCTACAAGGGAAAGTGGCATGGTGATGTG
GCAGTGAAAATGTTGAATGTGA**CAGCACCTACACCTCAGCAG**TTACAAGCCTTCAAAAATGAAGTAGGAGTA
CTCAGGAAAACACGACATGTGAATATCCTACTCTTCATGGGCTATTCCACAAAGCCACAACTGGCTATTGTT
ACCCAGTGGTG**TGAGGGCTCCAGCTTGTATC**ACCATCTCCATATCATTGAGACCAAATTTGAGATGATCAAA
CTTATAGATATTGCACGACAGACTGCACAGGGCATGGATTACTTACACGCCAAGTCAATCATCCACAGAGAC
CTCAAGAGTAATAATATATTTCTTCATGAAGACCTCACAGTAAAAATAGGTGATTTTGGTCTAGCTACAGTG
AAATCTCGATGGAGTGGGTCCCATCAGTTTGAACAGTTGTCTGGATCCATTTTGTGGATGGCACCAGAAGTC
ATCAGAATGCAAGATAAAAATCCATACAGCTTTCAGTCAGATGTATATGCATTTGGAATTGTTCTGTATGAA
TTGATGACTGGACAGTTACCTTATTCAAACATCAACAACAGGGACCAGATAATTTTTATGGTGGGACGAGGA
TACCTGTCTCCAGATCTCAGTAAGGTACGGAGTAACTGTCCAAAAGCCATGAAGAGATTAATGGCAGAGTGC
CTCAAAAAGAAAAGAGATGAGAGACCACTCTTTCCCCAA
GAGAATTTGCAGCCTTCAAGTAGCCACCATCATGGCAGCATCTGCTCTTATTTCTTAAGTCTTGTGTTCGTA
CAATTTGTTAACATCAAAACACAGTTCTGTTCCTCAAATCTTTTTTTAAAGATACAAAATTTCCAATGCATA
AGC**TGA**TGTGGAACAGA**ATGGAATTTCCCATCCAACA**AAAGAGGAAAGAATGTTTTAGGAACCAGAATTCT**C**
TGCTGCCAGTGTTTCTTCAACAAAAATACCACGAGCATACAAGTCTGCCCAGTCCCAGGAAGAAAGAGGAGA
GACCCTGAATTCTGACCTTTTGATGGTCAGGCATGATGGAAAGAAACTGCTGCTACAGCTTGGGAGATTTGC
TATGGAAAGTCTGCCAGTCAACTTTGCCCTTCTAACCACCAGATCAATTTGTGGCTGATCATCTGATGGGGC
AGTTTCAATCACCAAGCATCGTTCTCTTTCCTGTTCTGGAATTTTGTTTTGGAGCTCTTTCCCCTAGTGACC
ACCAGTTAGTTTCTGAGGGATGGAACAAAAATGCAGCTTGCCCTTTCTATGTGGTGCGTGTTCAGGCCTTGA
CAGATTTTATCAAAAGGAAACTATTTTATTTAAATGGAGGCTGAGTGGTGAGTAGATGTGTCTTGGTATGGA
GGAAAAGGGCATGCTGCATCTTCTTCCTGACCTCCGGGGTCTCTGGCCTTTTGTTTCCTTGCTCACTGAGGG
GTCTGTCTAACCAAGCAGGCTAGATAGTGCTGGCACACATTGCCTTCTTTCTCATTGGGTCCAGCAATGAAG
ATAAGTGTTTGGGTTTTTTTTTTTTCCTCCACAATGTAGCAAATTCTCAGGAAATACAGTTTATATCTTCCT
CCTATGCTCTTCCAGTCACCAACTACTTATGCGGCTACTTTGTCCAGGGCACAAAATGCCGTGGCAGTATCT
AACTAAACCCCCACAAAACTGCTTAATAACAGTTTTGAATGTGAGAAATTTAGATAATTTAAATATAAGGTA
CAGGTTTTAATTTCTGAGTTTCTTCTTTTCTATTTTTATTAAAAAGAAAATAATTTTCAGATTTAATTGAAT
TGGAAAAAAACAATACTTCCCACCAGAATTATATATCCTGAAAATTGTATTTTTGTTATATAAACAACTTTT
AAGAAAGATCATTATCCTTTTCTCTACCTAAATATGAGGAGTCTTAGCATAATGACAAATATTTATAATTTT
TCAATTAATGGTACTTGCTGGATCCACACTAACATCTTTGCTAATAATCTCATTGTTTCTTCCAACTGATTC
CTAACACTATATCCCACATCTTCTTTCTAGTCTTTTATCTAGAATATGCAACCTAAAATAAAAATGGTGGCG
TCTCCATTCATTCTCCTTCTTCCTTTTTTCCCAAGCCTGGTCTTCAAAAGGTTGGGCAATTTGGCAGCTGAA
TTCCCAGACAGAGAATAGAGCAATTTTAGGGATATTAGGACTGAGGGAGGGTGTGGGAAAGCTGTCATCAGT
TGTTTTTATAGAAAGAACTGGCATTCATTAAGAACCTAAATCTTATCTTTGCACAAATGGAAAATATAACCT
AGTTATAGCTTCCTTTGGCCTTTATTAAAGGGTAATATCAATCACAGTCATAGCAAAGAAAGCGGATGTATT
AATGGCAAATTAATGGAAAACCTCCCTTATCAGGAATCTAGACTCAGAATTTAGGAACACAAATCAAATCAG
ACCAACCAAGCTATAGCCAAGGACTTGAAAGAAATTAAACAAGACCCAGAATAAATCAAGGAATTAGAAATT
GTTATTTAAAAATTTCAGATTGTAACTCCAGGCCCTGCTGTCTATATTGCAGCCACTAAAAGCTCACTACCA
TTAGATTTTTGCTAACATACATGTATTCAGAAGAAAGCCTATTGAAATTTTCATTGTCTTGTAAAAGGTTGT

CCTAGTAAAATGGAAAAGATCCTTAAGTTATTAATCAGTTTGAAAAGCAAATTTGTTTTTAAGTTTTACATC
AGCAGGGCAGTGTCTTACAAAATTCAGAAATTGCAAAGGTGGAAATAATTCACGCTGATTTGAAGAACATCT
TCTGTGCAATAATACTGCCTCTCTTGAAAAGCATTGGCTGTTTTTTCTTTTTAAATATATCTCTAGATGCTT
TTAAATGTGGCTGTGTTCCCTTTACCAAGATTGGCTTCAAGTTTCCGCAGGTAGAGAGACCTGGGCTTGAAC
AAGAGGATGTGTTTCATGTCCTGCTGAGGAGGTAGAACATGTGCAGCCTGGGTCCGGGACTGCCTCCGTGGG
GCAGGGGCAGGGGCGGTACCATTAGGGAGGAAGCTTAGCATTTCAGTTTCTTAAACAATATTCAGGGTGATA
CACTTTTTCTTCCCTTGCATTTTAGAATAGGCTGGTATCTCATTTGAACGGGGGAGCAGACTTGATCTCAAA
TGAAGCTGTGCCCAGGAGCCAGGCTTAGCATATTGAGATTTTTATAGATACCTTAAAAAATAAAATATTTAA
ACCTCTCTTTTCTTCCTTTTTCTATGAAATAGGTTTTTTCTCTAGTTTACAAATGACATGAAAATAGGTTTT
ATTTGTGTTTTATCTGCTTTATTTTTTGATGCTTAGACAACAGTTAGACTTACTGAGCTCCTAAAAAAACGA
GGAAGAAGTCCTTATTTGTGAAAAGCACTTTATGAGTAATTGTATAGACAGTATGTGGCTGCGTCACTGATC
ATCTTGTAAGGGTGTAACAGTCTTGTCTGTAAAGTGGCTGCAGTGCCTTCTGTAGTGTGTTTTATTTTTGGT
AGGGAGAGGTGAAGCCTTCTGAAAAATTTGAGAGCAACTACAGAGGATTGTTTGTAACTGTGTAGTATTCCT
GATGGACTTTTTTCATCGTTAGAGTCAAGGACCTAGACTTTTGCCACTGAAATAATATTGACCAAAAAAATA
GTTTATAAAAGGGATTTGTGAATAGAAAATTCAGTGTGATCATTTGTTGTTAATGTGCACCTTAAAAGAAGA
TTCTGTCTAGCTGTCAAATTCTGGTTCCCGAATATCTCACCCCTGATTGTATTTGAGATCTAGTAGGGCATA
CTGGGGCATTTTAGAAGATAAAATCCCATACAAATGATATATGCTATATTTATGTTGGTGTTGGAGAAGAAA
GAGCAGTATATAAAGAAATAATTCAAGACTGCAGCACTGTCAACCTGAAACTTTGTAAATATT**TCCTAGCTT**
CTGGTTTGGTGCGGTGACAGCACTTTCATCACAGGATGTTACCTTGTATTCACCAGGCGGAGTGCGAGCTG**C**
TGCACATCCTCCTCAGATCTCACCTGTCCCCACTGTACATCCACCCGCCAGCTGCTTGCAAACCTCATCTCT
AGCTTTAGTTCGAAACCACATTGCAGGGTTCAGGTGACCTCTACAAAAAACTACCTCTTCAGAATGAGGTAA
TGAATAGTTATTTATTTTAAAATATGAAAAGTCAGGAGCTCTAGAACATGACGATGATTTAAGATTTTAACT
TTTTTGTGTACTTGTATTTGAGCACTCTCATTTTGTCCTAAAGGGCATTATACATTTAAGCAGTAATACTGT
AAAAAAATGTGTTGCTCGGAATATCTGAATGTTGTTGAAAGTGGTGCCAGAACCGGTTTAGGGGTACGTTTC
AGAATCTTAACCTTGAGTCAATTGCATGAAATTAAATAGCTGTGGTATCACTTCACTAACAGTGATGTAATT
TTAATTTTCAGTAGGCTTGGCATGACAGTACATCCTCATAATGAGTTTGCTGCAGCTTTGTCACATGCACAG
GCATTCATAGAAAGACCACCCAGCTAAGAGGGTAGAATGATTACTCTTTTTGCAAGATTCTCTTCTTTGTCC
AAGTTGGCATTGTTAGTGCTAGGAATACCAGCACCTTGAGACGAGCAGATTCCAACCATTAGGCTATAAACA
CCATAGCCAGAGATGGAAGGTTTACTGTGAGTATGAACAGCAAATAGCTTACAGGTCATGAGTTGAAATGGT
GTAGGTGAGGCTCTAGAAAAATACCTTGACAATTTGCCAAATGATCTTACTGTGCCTTCATGATGCAATAAA
AAAGCTAACATTTTAGCAGAAATCAGTGATTTGTGAAGAGAGCAGCCACTCTGGTTTAACTCAGCTGTGTTA
ATAATTTTTAGAGTGCAATTTAGACTGCATAGGTAAATGCACTAAAGAGTTTATAGCCAAAATCACATTTAA
CAATGAGAAAACACACAGGTAAATTTTCAGTGAACAAAATTATTTTTTTAAAGCACATAATCCCTAGTATAG
TCAGATATATTTATCACATAGAGCAACTAGGTTGCAAATATAGTTCAGTGACATTTCTAGAGAAACTTTTTC
TACTCCCATAGGCTCTTCAAAGCATGGAACTTTTATACAACAGAAATGTTGACAGAAATTGCTGTAGTTTAG
GGTTGAAGTACTGTATGATGGGCAGCAATCATGTATTAACTTAGAAGGGGAAATTGAAATATAGGACCGAAT
TTGGTTTTATCAGTTTCCAGAGTACTGCTGCCAACCTAGACACTGATTTTTCAGAGTTTGAAATGTAAATTT
CTTCCCGGA**CTTGATTGCACATGAAGCTG**GACTGCGTTAGTCATCCTGTCCCAAAGCGCTGTGGGGGCCAG
GGTGGAGGTCTCAAGGC**ATCCTTTATGACCTGGCCAT**TGGATGTAAAAGAAAACATATTCCATGCTGTGGTT
CTTGTATCTTGTTTCATTCCTCACCATTGAAAGAGAAAGTCCATGTATTGTCTCCAGCACATCCTTGAAATG
TTATACTGGGATGGATTACTGATGCCCATCGGTAGTTGAGCCCCAGAAGAGGGTAGTAGCATCTCTGCCTCA
GGTGATGATTTGTAGCTTGGCCAGAGGAGAGCGGAGTCACCAGTATATCTGTGGTCCATGTTGCTAGCTCTG
GTAAAATTAAAAATACTGGTAAGATGTTTGTTTTATTAGTACACTAGACAGTAAGCTCTGTTTTGTTGTTTT
CAAATAACCTATTTTCACTTTTGTTTGGGCAAAGACATTTAAATTGAAATTCAATTCTAATTTTTGTTAATT
GTGGAAAGGGTAATTAACAGTTCCTATCAGGTATTTTTAATGTGGAAAAGGACAGAAACCCAACTCCTAAAA
TCTTAAATTAAGGTAACAGTGCTTTAAAAAAAAAAAATGCATGGGGCAATTAGTCGGCAACTCAATGAGTGA
CTAAAGTACTTTTATTTAACATCCACAACTTCAACTGTTAAGTTTTATTAATTACTAAATCAGCTTTATTAA
AATGTTGACATTTATTTAGCTATTTTGAATAATTATAGTGACTTGACGAGTGTGTATGAGGACACAGCCAAT
GTAAGCCAGTGTATCCATTTTTTAGAGGTGCATTTTTTTTTAAAGAATTCTGTAGATAGAAGTGCTCTGAAA
ACAACTAAAATATGTTTATTCATGGTAGTATCAAAAAAATGTTTGTACAAACCATCTGCTTCTCCCGGCCAGC
CGAGTTCATTCTCCAGCACCGTGACCGCTGGTTCTCATGTACAGCACATATGCGGGAGAGTTGGCAGAAAAT
TTGTGAAGAGATGCCGCAAAGGAAGGGTCTGTTGACGGGTGGGATTGGGGGTTTTGATGAAGTTGCTTAGTC
CTGGTTTTGTTTTGAAAATTACTGCGTTGCATTTTTGTGTTAAGTTTTTGAACCCACGTGTGTTTTGGTGGA
GTATGAGTTGGAAGTCACTGCAAACTAGCATAAACAACAAAGCTCACAGAGTAGGCACAGATGTAGAGAACA
GAGACCAAAATGGGGTGAGGTGGCAGTAAATCTAGGATAGGGAAAAATTAATGTGAGGGTGGGAAATAAACT
GTAATTACCTGAAATCAAATGTAAGAGTGCAATAAGTATGCTTTTTATTCTAAGCTGTGAACGGTTTTTTTA
AGAATCATTCCTTCCTAATACATTTGTGTATGTTCCATAGCTGATTAAAACCAGCTATATCAACATATAATG
CCTTTTTATTCATGTTAATGACCAACGTAAGTGGCTAGCCTTTATGTCTTATTTATCTTCATGTTATGTTAG

TTTACATACAGGGGTGTATGTCTCTGTGCTGTCCCCTTCTCCTGCCTTCATTTTAAAATGCATCCATGGGTC
CTCCGTGTTTCCTTTGGCCATGCCACATATATAGACTCAGTTTGGCCTTCATGATATCGCCTGATTTTTGAG
GACTGTATCACAGTGATATGTATTTGTGGTAATCTCATTTGTTGGTTGTACATCTGATCCTTTCCTCAACAT
GGCAATTGCTGCCTTTCCTAAGATAGGATCATACAACTGATCAGGGGATTGAATTTGATCATTCATCAACAT
GTGTCTCTGAATTTTATTCAGTAGTTGTCATTGCTCTTTGGTTTAGACCAAGAAAAAGGAAATCCCCCCTTT
TCATGTATTCCTTGGTTTGAGGACATGACTCCTGTAAGGGAGAGGAAAGGGAGATGCTTCCTGTTTGAACTG
CAGTGAATTCACGGTTCCTGTTTCACCACTCCAAACC**TTATGGCGACTCACACACAC**ATTCCTCTTTTCTGT
TACTGCCAAAGGTTCGGGTTTAGTACACTTCAGTTCCACTCAAGCA**TTGAAAAGGTTCTCGTGGAG**TCTGGG
GCGTGCCCAGTGAAAAGATGGGGACTTTTTAATTGTCCACAGACCTCTCTATACCTGCTTTGCAAAAATTAC
AATGGAGTAACTATTTTTAAAGCTTATTTTTCAATTCATAAAAAAGACATTTATTTTCAGTCAAATGGATGA
TGTCTCCCTCTTTTCCCCTATTCTCAATGTTTGCTTGAATCTTTTATTATTTTTTTTAATTCTCCCCCATAC
CCACTTCCTGATACTTTGGTTCTCTTTCCTGCTCAGGTCCCTTCATTTGTACTTTGGAGTTTTTCTCATGTA
AATTTGTATAACAGAAAATATTGTTCAGTTTGGATAGAAAGCATGGAGAATAAAAAAAGATAGCTGAAATTC
AGATTGAAGAAATTTATTTCTGTGTAAAGTTATTTAAAAACTGTATTATATAAAAGGCAAAAAAAGTTCTAT
GTACTTGATGTGAATATGCGAATACTGCTATAATAAAGATTGACTGCATGGAGAA

START codon
Exon 17
Exon 19
STOP codon

si-tot*BRAF*
si-fl*BRAF*
si-*BRAF*-E19-1/2/3

primers tot*BRAF* qRT-PCR F/R: 153bp
primers *BRAF*-X2 qRT-PCR F/R: 105bp
primers *BRAF*-E19-1/2/3/4 qRT-PCR, 4 F/R pairs: 74, 100, 99, 101bp
primers fl*BRAF* qRT-PCR F/R: 115bp

primers *BRAF*-E7, E8, E9-10
used with *BRAF*-E19-1 qRT-PCR R:
1452, 1351, 1202bp

primers *BRAF*-E19-1/2/3/4: 4 F/R pairs, 623, 565, 609, 595bp
BRAF-E19-1 F coincides with *BRAF*-E19-1 qRT-PCR F
